# Supplementary material for: An Afferent Neuropeptide System Transmits Mechanosensory Signals Triggering Sensitization and Arousal in C. elegans
Source: Neuron. 2018 Sep 19;99(6):1233–1246.e6. doi: 10.1016/j.neuron.2018.08.003 (PMC6162336; doi:10.1016/j.neuron.2018.08.003)
Supplement: Document S1. Figures S1–S7 and Tables S1 and S2 [file mmc1.pdf]

Neuron, Volume *99*

Supplemental Information

An Afferent Neuropeptide System Transmits Mechanosensory Signals Triggering Sensitization and Arousal in *C. elegans*

Yee Lian Chew, Yoshinori Tanizawa, Yongmin Cho, Buyun Zhao, Alex J. Yu, Evan L. Ardiel, Ithai Rabinowitch, Jihong Bai, Catharine H. Rankin, Hang Lu, Isabel Beets, and William R. Schafer

Figure S1: Related to Figure 1 Chew *et al.*

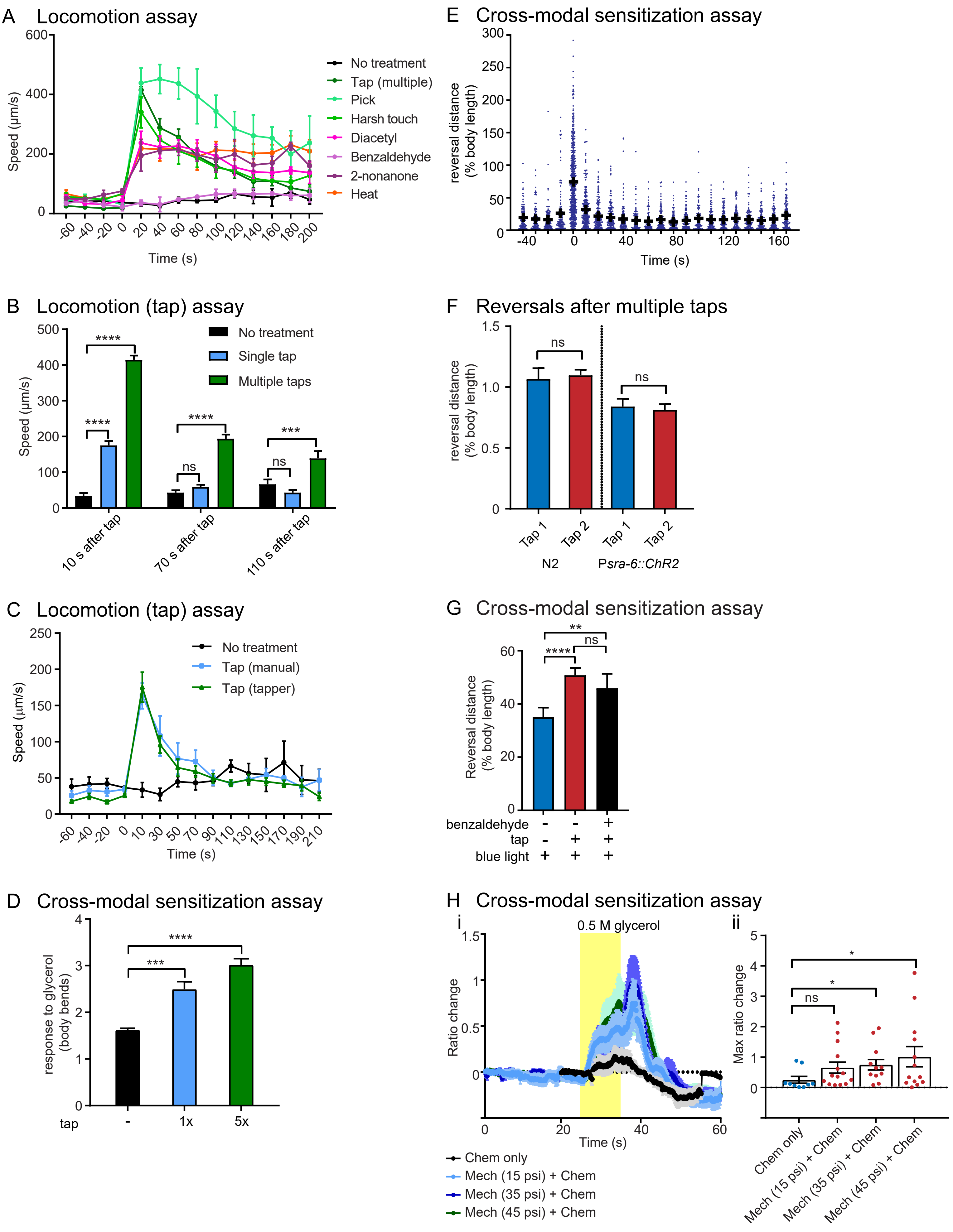

Figure S1, related to Figure 1: *C. elegans* displays locomotor and sensory arousal in response to aversive stimuli. A) Wild-type animals show an increased locomotion speed following the onset of various stimuli excluding benzaldehyde. Here, diacetyl is delivered at an aversive concentration (Yoshida et al., 2012). B) The locomotor response to tap stimulation in wild-type animals is dose dependent as animals exposed to multiple taps show a larger response that lasts longer than animals given a single tap. One-way ANOVA, Sidak's post-test: ns = not significant, ***<0.001, ****<0.0001. C) Speed trace for wild-type animals exposed to mechanical stimuli from manual tapping or automated tapper (see Experimental Procedures). For panels A-C, error bars indicate mean ± SEM, 5–10 animals were assayed in at least 5 trials for each condition. D-H: Cross-modal sensitization assay: D) Wild-type animals show increased reversal responses (expressed as number of body bends) to glycerol following a pre-arousing tap stimulus. One-way ANOVA, Sidak's post-test: ***<0.001, ****<0.0001. n=20 per condition, 4 trials. E) Controls exposed to tap (t=0) alone do not show increased reversal distance 20 s after tap. Animals received a 1x tap stimulus, following the protocol used for ASH sensitization experiments. "+" indicates the mean values for reversal distance. n>4 trials, each point represents an individual animal. F) Multiple taps do not appear to sensitize the reversal response in N2 and P*sra-6::ChR2* transgenic animals. n=5 trials. G) An attractive cue (benzaldehyde) does not affect sensitization in response to optogenetic activation of ASH after pre-exposure to a tap stimulus. Two-way ANOVA, Fisher's post-test: ns = not significant, **<0.01, ****<0.0001. n>15. For all panels, error bars indicate mean ± SEM. H) ASH neurons show a sensitized response to glycerol after exposure to a range of mechanosensory stimuli modulated by changing the pressure of the pneumatic valves (see Experimental procedures). Only the 15 psi condition did not show a statistically significant difference compared with controls exposed to glycerol alone. Note that previous work indicates that stimuli at pressures less than 40 psi are approximately equivalent to a tap stimulus, and pressures higher than 40 psi to harsh touch (Cho et al., 2018). Mean traces are shown in (i) and quantification of maximal responses is shown in (ii). For ease of comparison, the average trace during glycerol perfusion for non-pre-aroused controls is temporally aligned with that of other groups. For F-H, error bars indicate mean ± SEM.

Figure S2: Related to Figure 1 and 2

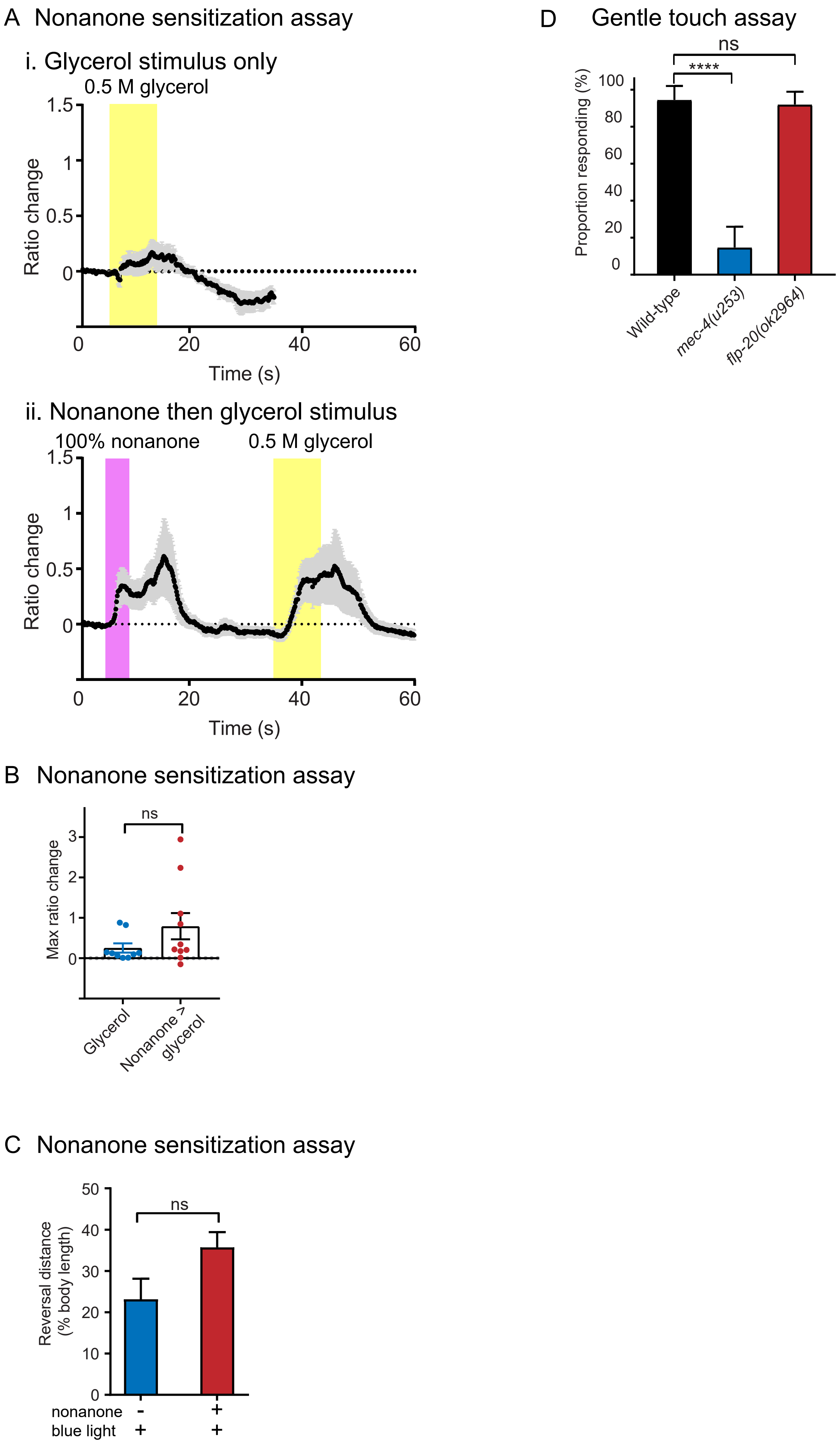

Figure S2, related to Figure 1 and 2: Aversive odorants do not sensitize ASH responses and FLP-20 is dispensable for response to gentle touch. A-C) Nonanone sensitization assay: Panel A shows the average traces of ASH calcium activity after exposure to glycerol either (i) alone or (ii) following a 5 s nonanone pulse (n = 9-10), panel B shows the quantification of maximal responses (p-value = 0.1465), and panel C shows the reversal distance for *ASH::ChR2* animals in response to optogenetic activation of ASH with or without pre-exposure to nonanone. Unpaired t-test: ns = not significant. n > 7. D) Touch assay for *flp-20(ok2964)* mutants compared with wild-type and *mec-4(u253)* controls showing the proportion of animals responding to alternating anterior and posterior body touch with an eyelash hair. One-way ANOVA, Sidak's post-test: ns = not significant, ****<0.0001. n=20. For all panels, error bars indicate mean ± SEM

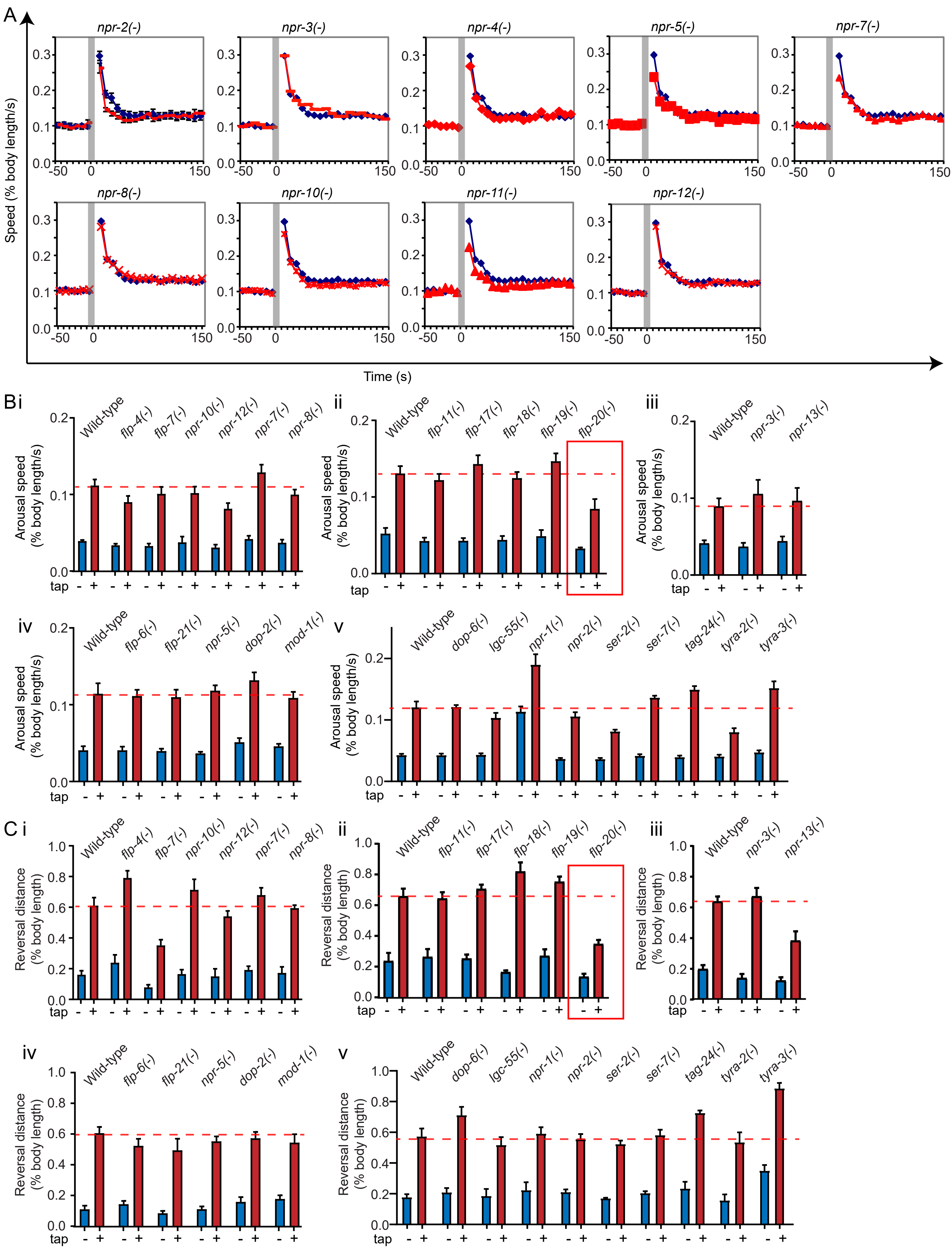
A
npr-2(-)
npr-3(-)
npr-4(-)
npr-5(-)
npr-7(-)
npr-8(-)
npr-10(-)
npr-11(-)
npr-12(-)
Speed (% body length/s)
Time (s)
B i
ii
iii
iv
v
Arousal speed (% body length/s)
Wild-type
flp-4(-)
flp-7(-)
npr-10(-)
npr-12(-)
npr-7(-)
npr-8(-)
flp-11(-)
flp-17(-)
flp-18(-)
flp-19(-)
flp-20(-)
npr-3(-)
npr-13(-)
flp-6(-)
flp-21(-)
npr-5(-)
dop-2(-)
mod-1(-)
dop-6(-)
lgc-55(-)
npr-1(-)
npr-2(-)
ser-2(-)
ser-7(-)
tag-24(-)
tyra-2(-)
tyra-3(-)
tap
C i
Reversal distance (% body length)

Figure S3, related to Figure 2: Candidate screen for mutant strains that show defects in arousal. A) Speed traces from a subset of the candidate mutant strains (red line) tested for locomotion speed in response to tap (onset of tap is shown by the vertical grey bar) compared to controls (blue line). Due to high variation in the baseline locomotion speed of these mutants, speed is normalized to the baseline speed before tap in all traces shown. n> 4 trials. B) Locomotion (tap) assay: quantified speed (%body length/s) before and after mechanical (tap) stimulation for the candidate mutant strains tested. C) Cross-modal sensitization assay: reversal distance (%body length) in response to optogenetic stimulation of ASH with blue light with or without a pre-arousing tap stimulus. All mutant strains were crossed into transgenic lines containing the *sra-6::ChR2* or *ASH::ChR2* transgene (see **Table S1** strain list). Experiments performed with different controls are shown in separate graphs. Dashed line indicates the values for controls in each experiment. *flp-20* mutants (red boxes) showed a reliable and clear difference in both locomotor and sensory sensitization compared with controls. n> 5 trials.

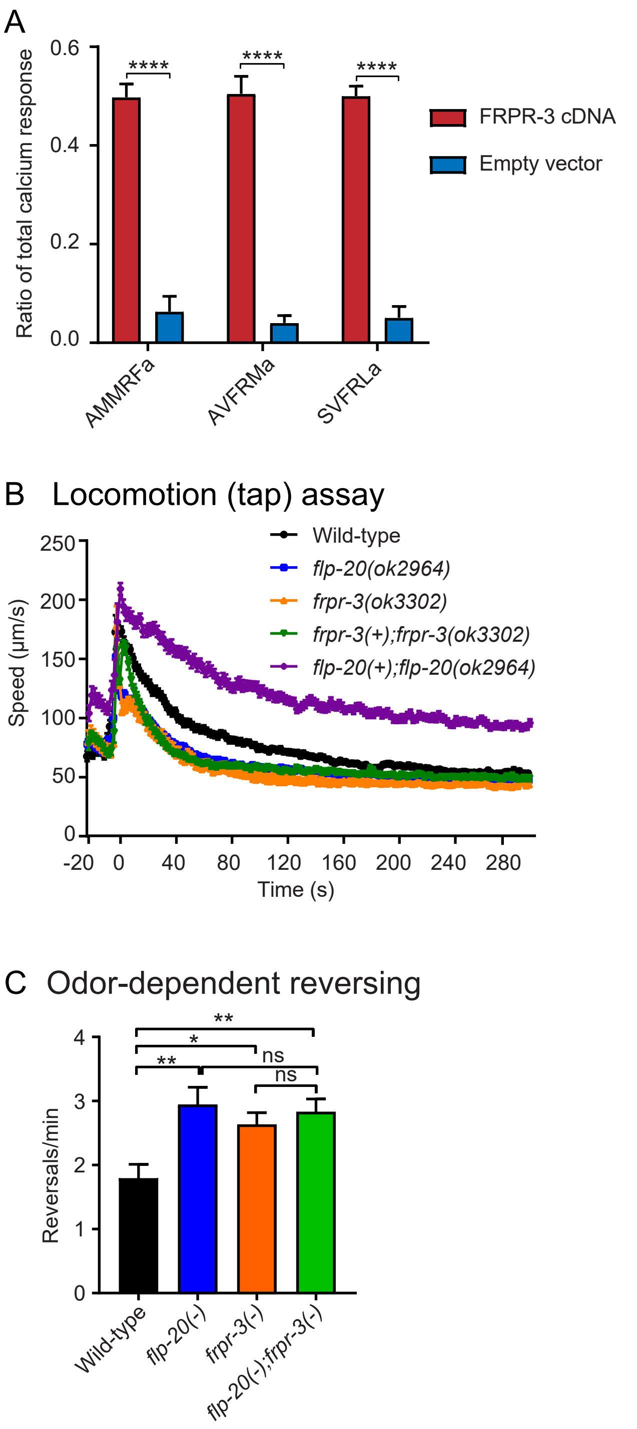
A
Ratio of total calcium response
0.6
0.4
0.2
0.0
****
****
****
FRPR-3 cDNA
Empty vector
AMMRFa
AVFRMa
SVFRLa
B Locomotion (tap) assay
Speed (µm/s)
250
200
150
100
50
0
-20 0 40 80 120 160 200 240 280
Time (s)
Wild-type
flp-20(ok2964)
frpr-3(ok3302)
frpr-3(+);frpr-3(ok3302)
flp-20(+);flp-20(ok2964)
C Odor-dependent reversing
Reversals/min
4
3
2
1
0
**
*
**
ns
ns
Wild-type
flp-20(-)
frpr-3(-)
flp-20(-);frpr-3(-)

Figure S4, related to Figure 4 and 5: FRPR-3 is required for locomotor arousal and for FLP-20-dependent responses to attractive chemosensory cues. A) Activation of FRPR-3 by FLP-20 peptides does not require $G\alpha_{16}$. Expression of *frpr-3* cDNA in cells expressing aequorin but lacking $G\alpha_{16}$ showed robust calcium transients in response to FLP-20 peptides compared to controls (empty pcDNA3.1(+) vector). Calcium responses are displayed as a ratio of total responses. n = 6. Unpaired t-test: ****<0.0001. B) Speed traces for wild-type, *flp-20* and *frpr-3* mutant animals together with transgenic animals re-expressing *flp-20* or *frpr-3* using the endogenous promoter for these genes. The tap stimulus is applied at time = 0. n>200 for at least three trials. C) Off-food reversals for *flp-20* and *frpr-3* single mutants and *flp-20;frpr-3* double mutant animals. One-way ANOVA, Sidak's post-test: ns = not significant, *<0.05, **<0.01. For all panels, error bars indicate mean ± SEM

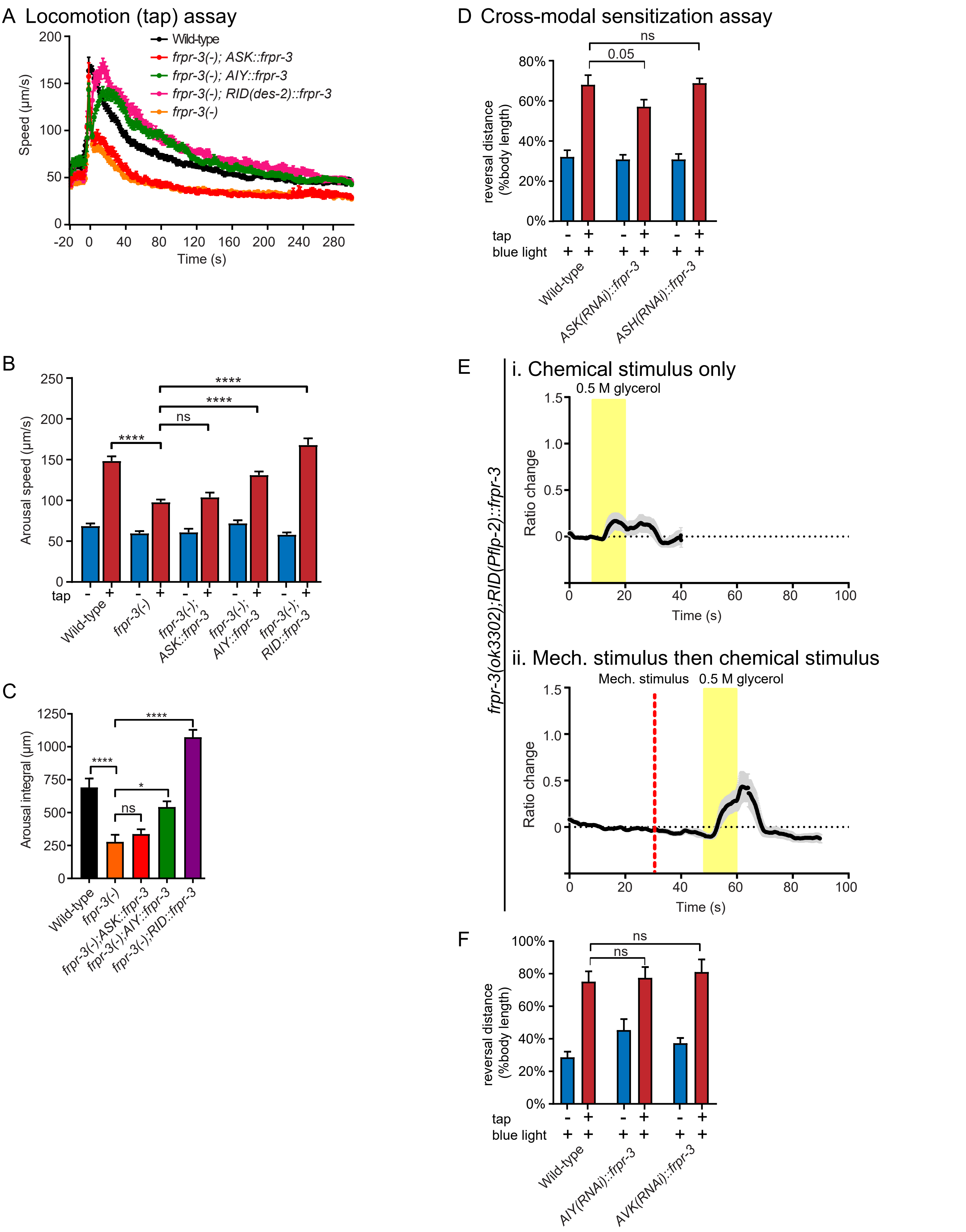
A Locomotion (tap) assay
Speed (µm/s)
Time (s)
Wild-type
frpr-3(-); ASK::frpr-3
frpr-3(-); AIY::frpr-3
frpr-3(-); RID(des-2)::frpr-3
frpr-3(-)
B
Arousal speed (µm/s)
tap
Wild-type
frpr-3(-)
frpr-3(-); ASK::frpr-3
frpr-3(-); AIY::frpr-3
frpr-3(-); RID::frpr-3
ns
****
C
Arousal integral (µm)
Wild-type
frpr-3(-)
frpr-3(-);ASK::frpr-3
frpr-3(-);AIY::frpr-3
frpr-3(-);RID::frpr-3
D Cross-modal sensitization assay
reversal distance (%body length)
tap
blue light
Wild-type
ASK(RNAi)::frpr-3
ASH(RNAi)::frpr-3
0.05
E
frpr-3(ok3302);RID(Pflp-2)::frpr-3
i. Chemical stimulus only
0.5 M glycerol
Ratio change
Time (s)
ii. Mech. stimulus then chemical stimulus
Mech. stimulus
F
AIY(RNAi)::frpr-3
AVK(RNAi)::frpr-3

Figure S5, related to Figure 6: FRPR-3 is required in RID and AIY for locomotor arousal and in RID for sensory sensitization. A-C) Locomotion arousal assays for wild-type and *frpr-3* mutant animals together with transgenic animals re-expressing *frpr-3* using the promoters driving expression in AIY (P*ttx-3*), ASK (P*sra-9*) or RID (P*des-2*). Panel A shows the speed traces for genotypes tested, with the tap stimulus applied at time = 0. Quantification of B) arousal speed and C) arousal integral after tap. For panels A-C, n> 200 for at least 2 trials. Error bars indicate mean ± SEM. One-way ANOVA, Sidak's post-test: ns = not significant, *<0.05, ****<0.0001. D-F) Cross-modal sensitization assays: D) Reversal responses for wild-type, *ASK(RNAi)::frpr-3* and *ASH(RNAi)::frpr-3* animals following blue light stimulation, with or without a pre-arousing tap. E) Mean traces of ASH calcium activity in *frpr-3(ok3302);RID(Pflp-2)::frpr-3* transgenic animals measured with GCaMP3 after exposure to glycerol either (i) alone or (ii) following a mechanical stimulus applied to the body of the animal. n=14-19. F) Reversal responses for wild-type, *AIY(RNAi)::frpr-3* and *AVK(RNAi)::frpr-3* animals following blue light stimulation, with or without a pre-arousing tap. For panels D,F: Two-way ANOVA, Fisher's post-test: ns = not significant. n>6 trials. Error bars indicate mean ± SEM.

A Micrographs for intersectional promoter strategy

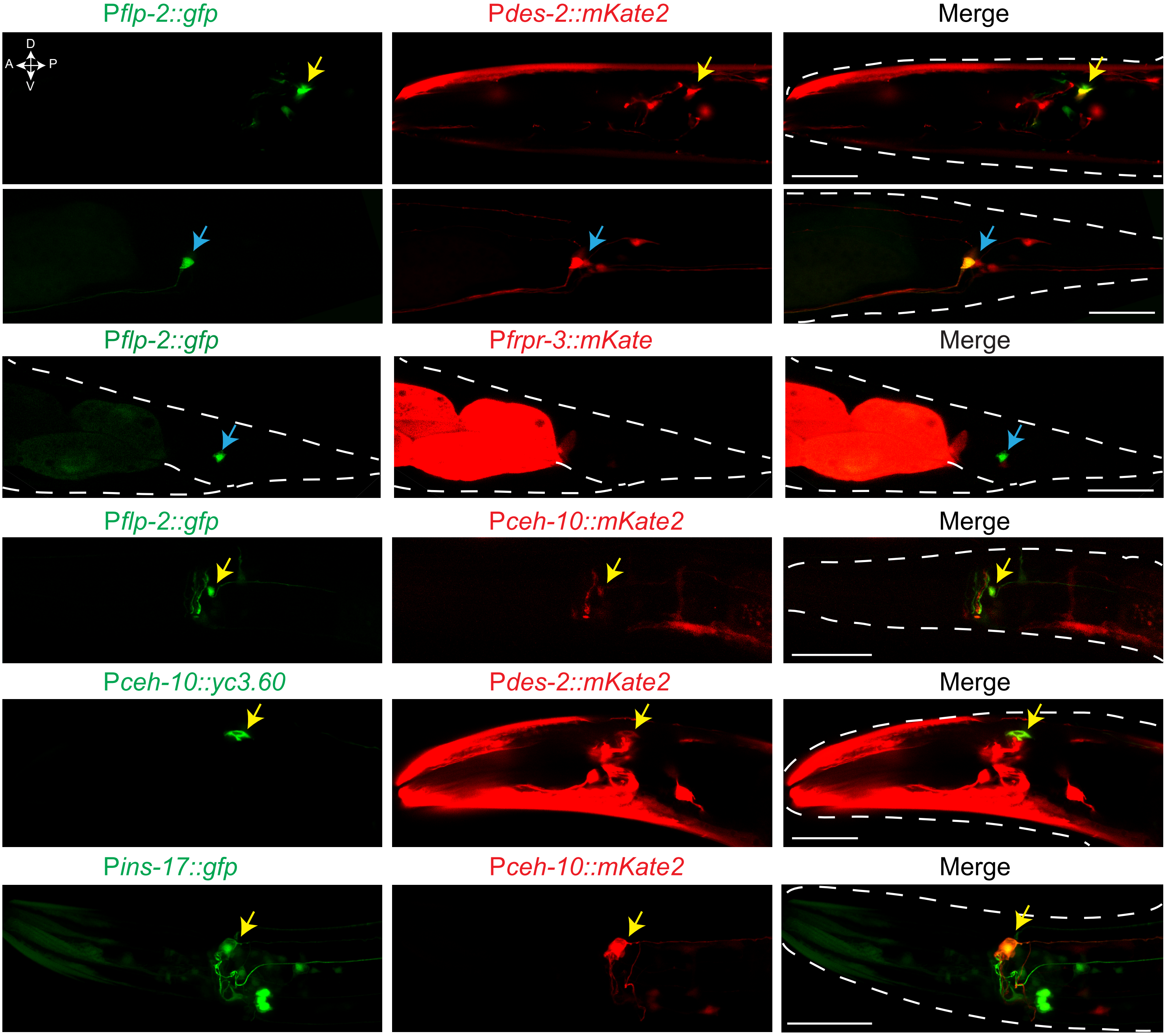

B Cross-modal sensitization assay

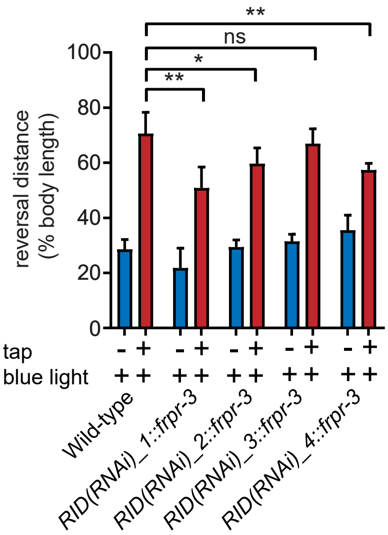

Figure S6, related to Figure 6: FRPR-3 is required in RID for ASH sensory sensitization. A) Micrographs showing the overlap between promoters used for the intersectional strategy for expression in RID. RID is indicated by the yellow arrow. P*flp-2::gfp*/P*des-2::mKate2* also overlap in an unidentified tail neuron (blue arrow) that does not colocalise with P*frpr-3::mKate2*. Scale bar = 10 µm. B) Cross-modal sensitization assay: Reversal responses following blue light stimulation for transgenic lines expressing antisense/sense RNAi constructs to knockdown *frpr-3* using four intersectional promoter combinations overlapping in RID, with or without a pre-arousing tap. The promoter combinations are: #1: P*flp-2/*P*des-2*; #2: P*flp-2/*P*ceh-10*; #3: P*des-2*/P*ceh-10*; #4: P*ins-17/*P*ceh-10*. See strain list **Table S1** for details. Error bars indicate mean ± SEM. Two-way ANOVA, Fisher's post-test: ns = not significant, *<0.05, **<0.01. n>4 trials.

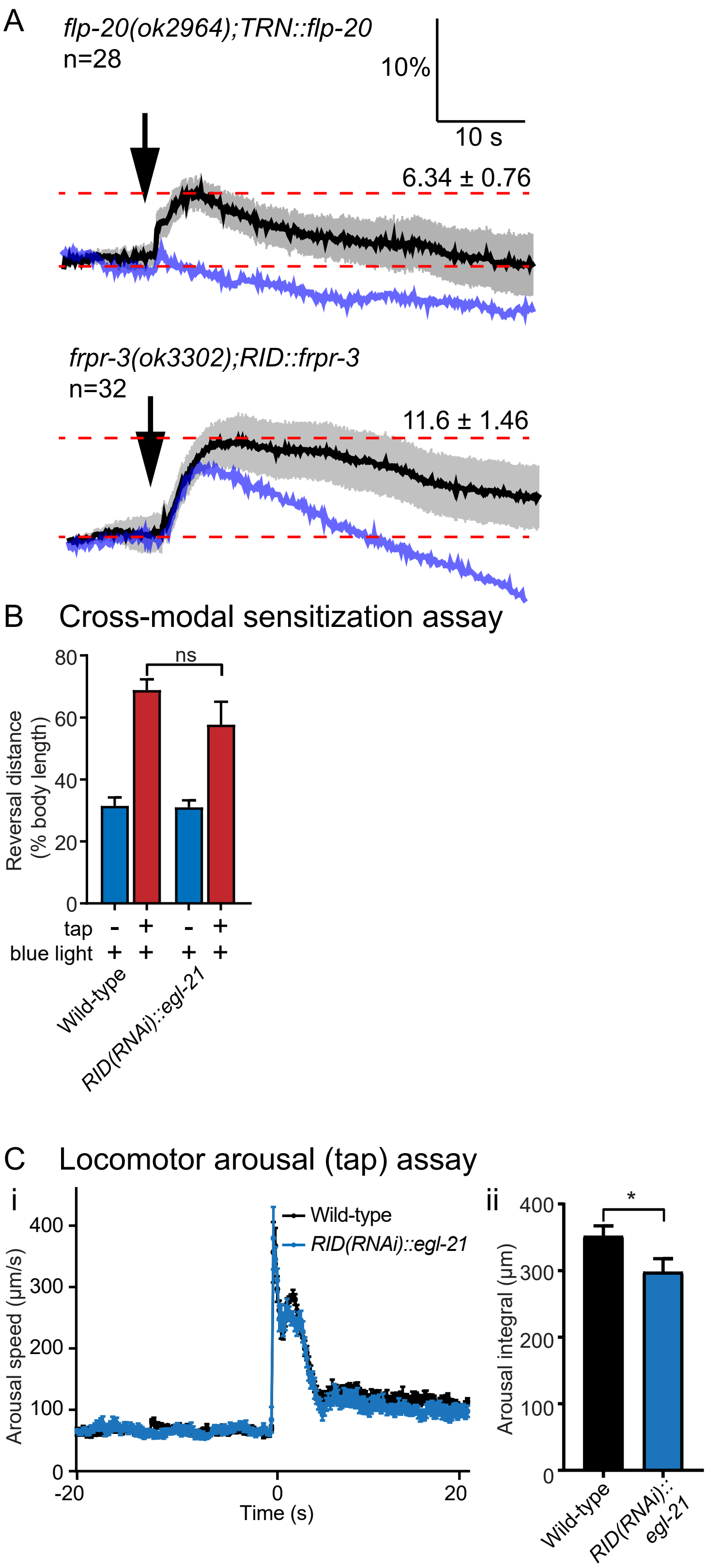
A
flp-20(ok2964);TRN::flp-20
n=28
10%
10 s
6.34 ± 0.76
frpr-3(ok3302);RID::frpr-3
n=32
11.6 ± 1.46
B Cross-modal sensitization assay
Reversal distance (% body length)
80
60
40
20
0
ns
tap - + - +
blue light + + + +
Wild-type
RID(RNAi)::egl-21
C Locomotor arousal (tap) assay
i
Arousal speed (μm/s)
400
300
200
100
0
-20
0
20
Time (s)
Wild-type
RID(RNAi)::egl-21
ii
Arousal integral (μm)
400
300
200
100
0
*
Wild-type
RID(RNAi):: egl-21

Figure S7, related to Figure 6: RID modulates arousal in response to mechanosensory stimulation via FLP-20/FRPR-3 and by signaling to downstream sensory targets partially via neuropeptides. A) Mean traces for transgenic lines re-expressing *flp-20* in the TRNs (using P*mec-4*) (*flp-20(ok2964);TRN::flp-20*) and *frpr-3* in RID (using P*ceh-10*) (*frpr-3(ok3302);RID::frpr-3*). For comparison, the mean traces (in blue) for the respective mutant strains are overlaid. The maximum ratio change for YFP/CFP comparing post-stimulus intensities with baseline intensities is shown as mean ± SEM for each trace. The number of worms tested is shown, with each worm stimulated 1-3 times. Scale shows % ratio change on the y-axis and time (s) on the x-axis. B) Cross-modal sensitization assay: Reversal responses for wild-type and *RID(RNAi)::egl-21* transgenic animals following blue light stimulation, with or without a pre-arousing tap. Two-way ANOVA, Fisher's post-test: ns = not significant. n = 6. C) i) Speed traces and ii) arousal integral for *RID(RNAi)::egl-21* and control animals after a 1x tap stimulus at t=0. Unpaired t-test: *<0.05. n = 6.

Table S1: Strain list, related to STAR Methods. For transgenic lines, the number following the transgene refers to the concentration injected in ng/µL.

Strain number	Genotype information	Notes	Experiment
AQ4045	*ljEx1107[Psra-6::GCaMP3::SL2-tagRFP(50);unc-122::RFP(50)]*		ASH calcium imaging (microfluidics)
AQ4076	*flp-20(ok2964);ljEx1107*		ASH calcium imaging (microfluidics)
AQ4077	*frpr-3(ok3302);ljEx1107*		ASH calcium imaging (microfluidics)
AQ4168	*frpr-3(ok3302);ljEx1149[Pdes-2::frpr-3cDNA::gpd-2 3' UTR(pYLC219)(25);unc-122::gfp(50)];ljEx1107*	*RID(+)::frpr-3*	ASH calcium imaging (microfluidics)
AQ4169	*frpr-3(ok3302);ljEx1150[Pflp-2::frpr-3cDNA::gpd-2 3' UTR(pYLC220)(25);unc-122::gfp(50)];ljEx1107*	*RID(+)::frpr-3*	ASH calcium imaging (microfluidics)
AQ4173	*flp-20(ok2964);ljEx1094[Pmec-4::flp-20 gDNA + 3' UTR::SL2-mKate2 (50);unc-122::gfp(50)];ljEx1107*	*TRN::flp-20*	ASH calcium imaging (microfluidics)
AQ2052	*lite-1(ce314);ljIs105[sra-6::ChR2::yfp, unc-122::gfp]*		Cross-modal sensitization assay
AQ2235	*lite-1(ce314); ljIs114[Pgpa-13::FLPase, Psra-6::FTF::ChR2::YFP] X*		Cross-modal sensitization assay
AQ2755	*lite-1(ce314); ljIs124[Pgpa-13::FLPase, Psra-6::FTF::ChR2::YFP]not X*		Cross-modal sensitization assay
VG266	*frpr-3(gk240031) (backcrossed 3x); lite-1(ce314); ljIs114*		Cross-modal sensitization assay
AQ2786	*flp-20 (ok2964) lite-1(ce314) X; ljIs124*		Cross-modal sensitization assay
AQ3941	*lite-1(ce314) flp-20(ok2964)X; ljIs124; Ex[Pmec-4::flp-20 cDNA)(20);unc-122::gfp(20)] line-1*	*TRN::flp-20*	Cross-modal sensitization assay
AQ3940	*lite-1(ce314) flp-20(ok2964)X; ljIs124; Ex[Pflp-20::flp-20 cDNA (20), ccGFP (20)] line-1*	*flp-20::flp-20*	Cross-modal sensitization assay
AQ4246	*lite-1(ce314) flp-20(ok2964)X; ljIs124; Ex[Pflp-20::flp-20 cDNA (20), ccGFP (20)] line-2*	*flp-20::flp-20*	Cross-modal sensitization assay
AQ4247	*lite-1(ce314) flp-20(ok2964)X; ljIs124; Ex[Pflp-20::flp-20 cDNA (20), ccGFP (20)] line-3*	*flp-20::flp-20*	Cross-modal sensitization assay
AQ4248	*lite-1(ce314) flp-20(ok2964)X; ljIs124; Ex[Pmec-4::flp-20 cDNA)(20);unc-122::gfp(20)] line-2*	*TRN::flp-20*	Cross-modal sensitization assay
AQ4249	*lite-1(ce314) flp-20(ok2964)X; ljIs124; Ex[Pmec-4::flp-20 cDNA)(20);unc-122::gfp(20)] line-3*	*TRN::flp-20*	Cross-modal sensitization assay
AQ4250	*lite-1(ce314) flp-20(ok2964)X; ljIs124; Ex[Pgcy-5::flp-20(10); Pgcy-7::flp-20(10);unc-122::gfp(20)] line-1*	*ASE::flp-20*	Cross-modal sensitization assay
AQ4251	*lite-1(ce314) flp-20(ok2964)X; ljIs124; Ex[Pgcy-5::flp-20(10); Pgcy-7::flp-20(10);unc-122::gfp(20)] line-2*	*ASE::flp-20*	Cross-modal sensitization assay
AQ4252	*lite-1(ce314) flp-20(ok2964)X; ljIs124; Ex[Pgcy-5::flp-20(10); Pgcy-7::flp-20(10);unc-122::gfp(20)] line-3*	*ASE::flp-20*	Cross-modal sensitization assay
AQ4260	*lite-1(ce314);ljIs124;ljEx1187[Psra-6::frpr-3 antisense RNAi(50);Psra-6::frpr-3 sense RNAi(50);ccGFP(30)]*	*ASH(RNAi)::frpr-3*	Cross-modal sensitization assay
AQ4262	*lite-1(ce314);ljIs124;ljEx1189[Psra-9::frpr-3 antisense RNAi(55);Psra-9::frpr-3 sense RNAi(55);ccGFP(30)]*	*ASK(RNAi)::frpr-3*	Cross-modal sensitization assay
AQ4264	*lite-1(ce314);ljIs124;ljEx1191[Pflp-2::frpr-3 antisense RNAi(50);Pdes-2::frpr-3 sense RNAi(50);ccGFP(30)]*	*RID(RNAi)_1::frpr-3*	Cross-modal sensitization assay
AQ4320	*lite-1(ce314);ljIs124;ljEx1214[Pdes-2::egl-21 antisense RNAi(50);Pflp-2::egl-21 sense RNAi(50);ccGFP(40)]*	*RID(RNAi)::egl-21*	Cross-modal sensitization assay
AQ4346	*lite-1(ce314);ljIs124;ljEx1228[Pflp-2::frpr-3 antisense RNAi(50);Pceh-10(3.6)::frpr-3 sense RNAi(50);ccGFP(50)]*	*RID(RNAi)_2::frpr-3*	Cross-modal sensitization assay
AQ4355	*lite-1(ce314);ljIs124;ljEx1229[Pdes-2::frpr-3 antisense RNAi(50);Pceh-10(3.6)::frpr-3 sense RNAi(50);ccGFP(50)]*	*RID(RNAi)_3::frpr-3*	Cross-modal sensitization assay
AQ4356	*lite-1(ce314);ljIs124;ljEx1230[Pins-17(2kb)::frpr-3 antisense RNAi(50);Pceh-10(3.6)::frpr-3 sense RNAi(50);ccGFP(50)]*	*RID(RNAi)_4::frpr-3*	Cross-modal sensitization assay
AQ4360	*lite-1(ce314);ljIs124;ljEx1234[Pflp-2::unc-31 antisense RNAi(50);Pdes-2::unc-31 sense RNAi(50);ccGFP(50))]*	*RID(RNAi)::unc-31*	Cross-modal sensitization assay
AQ4364	*lite-1(ce314);ljIs124;ljEx1238[Pttx-3::frpr-3 antisense RNAi(50);Pttx-3::frpr-3 sense RNAi(50);unc-122::gfp(50)]*	*AIY(RNAi)::frpr-3*	Cross-modal sensitization assay
AQ4365	*lite-1(ce314);ljIs124;ljEx1239[Pflp-1::frpr-3 antisense RNAi(50);Pflp-1::frpr-3 sense RNAi(50);unc-122::gfp(50)]*	*AVK(RNAi)::frpr-3*	Cross-modal sensitization assay
AQ4390	*lite-1(ce314);ljIs124;hpIs626(Pceh10::Chrimson::GFP::ZF; Pttx-3::ZIF-1::SL2::RFP; Pgpa-14::ZIF-1::SL2::RFP);hpEx3808(Parr-1-ZIF-1::SL2::RFP;Pmyo-3::rfp)*	AQ2755 x ZM9315	Cross-modal sensitization assay
AQ4023	*ljEx1093[Pflp-20::flp-20 gDNA + 3' UTR::SL2-mKate2(50);unc-122::gfp(50)]*		Expression pattern for *flp-20*
AQ4006	*ljEx1090[Pfrpr-3::frpr-3 gDNA::SL2-mKate(25); unc-122::gfp(50)]*		Expression pattern for *frpr-3*
TU253	*mec-4(u253) X*		Gentle touch
AQ4019	*frpr-3(ok3302);ljEx1090*	*frpr-3::frpr-3*	Locomotion (tap) arousal assay
AQ4035	*flp-20(ok2964);ljEx1094*	*TRN::flp-20*	Locomotion (tap) arousal assay
AQ4037	*flp-20(ok2964);ljEx1093*	*flp-20::flp-20*	Locomotion (tap) arousal assay
AQ4054	*frpr-3(ok3302);flp-20(ok2964)*		Locomotion (tap) arousal assay
AQ4072	*flp-20(ok2964);frpr-3(ok3302);ljEx1093*		Locomotion (tap) arousal assay
AQ4078	*frpr-3(ok3302);ljEx1108[Pflp-1::frpr-3 gDNA + UTR(10);unc-122::GFP(50)]*	*AVK::frpr-3*	Locomotion (tap) arousal assay
AQ4104	*frpr-3(ok3302);ljEx1135[Pflp-2::frpr-3::SL2-mKate2 (50);unc-122::gfp(50)]*	*RID(+)::frpr-3*	Locomotion (tap) arousal assay
AQ4105	*frpr-3(ok3302);ljEx1136[Pdes-2::frpr-3::SL2-mKate2 (50);unc-122::gfp(50)]*	*RID(+)::frpr-3*	Locomotion (tap) arousal assay
AQ4179	*frpr-3(ok3302);ljEx1167[Pceh-10(3.6)::frpr-3::SL2-mKate2(pYLC232)(50);unc-122::gfp(50)]*	*RID(+)::frpr-3*	Locomotion (tap) arousal assay
AQ4087	*frpr-3(ok3302);ljEx1123[Psra-9::frpr-3 gDNA + 3' UTR::SL2-mKate2(50);ccGFP(50)]*	*ASK::frpr-3*	Locomotion (tap) arousal assay
AQ4103	*frpr-3(ok3302);ljEx1134[Pttx-3::frpr-3::SL2-mKate2 (50)(pYLC190);ccGFP(50)]*	*AIY::frpr-3*	Locomotion (tap) arousal assay
AQ3832	*frpr-3(ok3302) V* backcrossed 6x		Locomotion (tap)
AQ4000	*flp-20(ok2964) X* backcrossed 6x		Locomotion (tap), gentle touch
AQ4396	*ljEx1246[Pdes-2::mKate2::gpd-2 3' UTR(25);ccGFP(50)];ljEx1247[Pflp-2:gfp::gpd-2 3' UTR(25);ccRFP(50)]*		Micrographs for RID intersectional promoter
AQ4397	*ljEx1246[Pdes-2::mKate2::gpd-2 3' UTR(25);ccGFP(50)];ljEx1165[Pceh-10(3.6)::YC3.60::gpd-2 3' UTR(25);ccRFP(50)]*		Micrographs for RID intersectional promoter
AQ4398	*unc-119(ed3) III; wwEx73(ins-17p::gfp + unc-119(+));ljEx1167[Pceh-10(3.6)::frpr-3::SL2-mKate2(50);ccGFP(50)]*	*ins-17::gfp* from HT1734	Micrographs for RID intersectional promoter
AQ4399	*ljEx1167[Pceh-10(3.6)::frpr-3::SL2-mKate2(50);ccGFP(50)];ljEx1247[Pflp-2:gfp::gpd-2 3' UTR(50);ccRFP(50)]*		Micrographs for RID intersectional promoter
PT505	*flp-20(pk1596) X*		Off food reversal assay
VC2565	*frpr-3(ok3302) V*		Off food reversal assay
BJH387	*flp-20(pk1596) X; frpr-3(ok3302) V*		Off food reversal assay
AQ4144	*ljEx1165[Pceh-10(3.6)::YC3.60::gpd-2 3' UTR(50);unc-122::rfp(50)]*		RID calcium imaging (glued protocol)
AQ4187	*frpr-3(ok3302);ljEx1165*		RID calcium imaging (glued protocol)
**Strain number**	**Genotype information**	**Notes**	**Experiment**

AQ4188	*flp-20(ok2964);ljEx1165*		RID calcium imaging (glued protocol)
AQ4210	*flp-20(ok2964);ljEx1094;ljEx1165*	*TRN::flp-20*	RID calcium imaging (glued protocol)
AQ4211	*frpr-3(ok3302);ljEx1167;ljEx1165*	*RID(+)::frpr-3*	RID calcium imaging (glued protocol)
AQ2766	*lite-1(ce314);ljIs114 X (ASH::ChR2);dop-2 (vs105)V*		Candidate screen
AQ2767	*lite-1(ce314);ljIs114 X (ASH::ChR2);npr-3(tm1583)*		Candidate screen
AQ2768	*lite-1(ce314);ljIs114 X (ASH::ChR2);npr-5 (ok1583)V*		Candidate screen
AQ2769	*lite-1(ce314);ljIs114 X (ASH::ChR2);npr-13 (tm1504)V*		Candidate screen
AQ2770	*lite-1(ce314);ljIs114 X (ASH::ChR2);flp-6 (ok3056) V*		Candidate screen
AQ2772	*lite-1(ce314);ljIs114 X (ASH::ChR2);flp-21 (ok889)V*		Candidate screen
AQ2773	*lite-1(ce314);ljIs114 X (ASH::ChR2);mod-1 (ok103)V*		Candidate screen
AQ2779	*lite-1(ce314);ljIs114 X (ASH::ChR2);flp-4 II*		Candidate screen
AQ2781	*lite-1(ce314);ljIs124 non-X (ASH::ChR2); tyra-3 (ok325)X*		Candidate screen
AQ2782	*lite-1(ce314);ljIs124 non-X (ASH::ChR2); npr-1 (ad609)X*		Candidate screen
AQ2783	*lite-1(ce314);ljIs124 non-X (ASH::ChR2); flp-7 (ok2625)X*		Candidate screen
AQ2785	*lite-1(ce314);ljIs124 non-X (ASH::ChR2); flp-18 (dp99)X*		Candidate screen
AQ2786	*lite-1(ce314);ljIs124 non-X (ASH::ChR2); flp-20 (ok2964)X*		Candidate screen
AQ4405	*ljIs105(Psra-6::ChR2); ser-7 (tm1325)X*		Candidate screen
AQ4406	*ljIs105(Psra-6::ChR2);tag-24 (ok371)X*		Candidate screen
AQ4407	*ljIs105(Psra-6::ChR2);ser-2 (pk1357)X*		Candidate screen
AQ4408	*ljIs105(Psra-6::ChR2);tyra-2 (tm1846)X*		Candidate screen
AQ4409	*ljIs105(Psra-6::ChR2);tyra-3 (ok325)X*		Candidate screen
AQ4410	*ljIs105(Psra-6::ChR2);lgc-55 (tm2913)V*		Candidate screen
AQ4411	*ljIs105(Psra-6::ChR2);dop-6/C24A8.1 (ok2090)X*		Candidate screen
AQ4412	*ljIs105(Psra-6::ChR2);npr-1 (ad609)X*		Candidate screen
AQ4413	*ljIs105(Psra-6::ChR2);npr-2 (ok419)IV*		Candidate screen
AQ4414	*ljIs105(Psra-6::ChR2);npr-7 (ok527)X*		Candidate screen
AQ4415	*ljIs105(Psra-6::ChR2);npr-8 (tm1553)X*		Candidate screen
AQ4416	*ljIs105(Psra-6::ChR2);npr-10 (tm1568)X*		Candidate screen
AQ4417	*ljIs105(Psra-6::ChR2);npr-11 (ok594)X*		Candidate screen
AQ4418	*ljIs105(Psra-6::ChR2);npr-12 (tm1498)IV*		Candidate screen
AQ4419	*ljIs105(Psra-6::ChR2);flp-11 (tm2706)X*		Candidate screen
AQ4420	*ljIs105(Psra-6::ChR2);flp-17 (ok3587)IV*		Candidate screen
AQ4421	*ljIs105(Psra-6::ChR2);flp-18 (dp99)X*		Candidate screen
AQ4422	*ljIs105(Psra-6::ChR2);flp-19 (ok2460)X*		Candidate screen

Table S2: Reporter lines used for identification of *flp-20* and *frpr-3*- expressing cells, related to STAR Methods.

Gene	Cell	Promoter	Reporter line
flp-20	TRNs	*mec-4*	AQ906 *bzIs17[Pmec-4, yc2.12+lin-15]*
	PVC	*nmr-1*	AQ2307 *ljEx226[Pnmr-1::YC3.60]*
	LUA	*inx-11*	ZW291 *zwEx111[Pinx-11:GFP + lin-15(+)]*
	ASE	*gcy-7*	AQ4095 *ljEx1129[Pgcy-7::gfp]*
frpr-3	AVK	*flp-1*	NY2097 *ynIs97 [Pflp-1::GFP]*
	AIY	*ttx-3*	AQ3040 *ljEx521[Pttx-3::YC3.60]*
	RID	*ceh-10*	LE332 *lqIs10[Pceh-10::GFP + lin-15(+)]*
	ASK	*sra-9*	AQ3093 *ljEx543[Psra-9::YC3.60]*
