# Supplementary material for: An Afferent Neuropeptide System Transmits Mechanosensory Signals Triggering Sensitization and Arousal in C. elegans
Source: Neuron. 2018 Sep 19;99(6):1233–1246.e6. doi: 10.1016/j.neuron.2018.08.003 (PMC6162336; doi:10.1016/j.neuron.2018.08.003)
Supplement: Document S2. Article plus Supplemental Information [file mmc2.pdf]

Neuron

Article

An Afferent Neuropeptide System Transmits Mechanosensory Signals Triggering Sensitization and Arousal in *C. elegans*

Highlights

- A novel arousal paradigm in *C. elegans* requires a cascade of neuropeptide signaling
- Mechanosensory stimulation leads to long-lasting motor and sensory sensitization
- Arousal requires FLP-20 peptides released by touch neurons and receptor FRPR-3
- FRPR-3 activity then confers behavioral state information via the interneuron RID

Authors

Yee Lian Chew, Yoshinori Tanizawa, Yongmin Cho, ..., Hang Lu, Isabel Beets, William R. Schafer

Correspondence

wschafer@mrc-lmb.cam.ac.uk

In Brief

Arousal is an important conserved behavioral state where animals show increased sensory responsiveness and locomotor hyperactivity. Chew et al. identify a neuromodulatory pathway that enables mechanosensory neurons to promote both sensory and locomotor arousal via activation of a neuroendocrine center.

Chew et al., 2018, Neuron *99*, 1233–1246
September 19, 2018 © 2018 MRC Laboratory of Molecular Biology.
Published by Elsevier Inc.
https://doi.org/10.1016/j.neuron.2018.08.003

CellPress

CellPress

An Afferent Neuropeptide System Transmits Mechanosensory Signals Triggering Sensitization and Arousal in *C. elegans*

Yee Lian Chew,[1] Yoshinori Tanizawa,[1] Yongmin Cho,[2] Buyun Zhao,[1] Alex J. Yu,[3] Evan L. Ardiel,[3] Ithai Rabinowitch,[7,8] Jihong Bai,[7] Catharine H. Rankin,[3,4] Hang Lu,[2] Isabel Beets,[5,6] and William R. Schafer[1,9,*]
[1]Neurobiology Division, MRC Laboratory of Molecular Biology, Cambridge, Cambridgeshire, CB2 0QH, UK
[2]School of Chemical and Biomolecular Engineering, Georgia Institute of Technology, Atlanta, GA 30332-0100, USA
[3]Djavad Mowafaghian Centre for Brain Health, University of British Columbia, Vancouver, BC V6T, Canada
[4]Department of Psychology, University of British Columbia, Vancouver, BC V6T 1Z4, Canada
[5]Cell Biology Division, MRC Laboratory of Molecular Biology, Cambridge, Cambridgeshire, CB2 0QH, UK
[6]Department of Biology, Division of Animal Physiology and Neurobiology, KU Leuven, B-3000, Leuven, Belgium
[7]Division of Basic Sciences, Fred Hutchinson Cancer Research Center, Seattle, WA 98109, USA
[8]Department of Medical Neurobiology, Faculty of Medicine, Hebrew University of Jerusalem, Jerusalem 91120, Israel
[9]Lead Contact
*Correspondence: wschafer@mrc-lmb.cam.ac.uk
https://doi.org/10.1016/j.neuron.2018.08.003

SUMMARY

Sensitization is a simple form of behavioral plasticity by which an initial stimulus, often signaling danger, leads to increased responsiveness to subsequent stimuli. Cross-modal sensitization is an important feature of arousal in many organisms, yet its molecular and neural mechanisms are incompletely understood. Here we show that in *C. elegans*, aversive mechanical stimuli lead to both enhanced locomotor activity and sensitization of aversive chemosensory pathways. Both locomotor arousal and cross-modal sensitization depend on the release of FLP-20 neuropeptides from primary mechanosensory neurons and on their receptor FRPR-3. Surprisingly, the critical site of action of FRPR-3 for both sensory and locomotor arousal is RID, a single neuroendocrine cell specialized for the release of neuropeptides that responds to mechanical stimuli in a FLP-20-dependent manner. Thus, FLP-20 peptides function as an afferent arousal signal that conveys mechanosensory information to central neurons that modulate arousal and other behavioral states.

INTRODUCTION

Sensitization is a simple form of behavioral plasticity by which exposure to an initial stimulus leads to an enhanced response to a second stimulus. Sensitization is critical for survival, as it allows adjustments to sensory responsiveness in a changing environment, and like other simple forms of plasticity it may serve as a building block for more complex forms of learning and memory. Sensitization has been observed in a variety of organisms from invertebrates to humans (Carew et al., 1971; Hubbard et al., 2011; Rankin et al., 1990), indicating the importance and widespread conservation of this behavior. Both monoamine and peptide neuromodulators have been implicated in driving this form of plasticity (see, for example, Barbas et al., 2003; Im et al., 2015). However, many questions remain concerning the molecular and neural mechanisms of sensitization and how it contributes to behavioral states, in particular to arousal.

Arousal designates a change in behavioral state that enhances vigilance and the ability to respond to appetitive (e.g., food/mates) or aversive (i.e., danger) stimuli. Hallmarks of arousal include sensitization in the form of enhanced sensory acuity, increased motor activity, and greater reactivity to external stimuli. Arousal is a highly conserved, possibly universal feature of animal nervous systems (Horstick et al., 2016; Pfaff et al., 2008) and is thought to provide a crucial mechanism through which animals can respond appropriately to their environment. Arousal can be ‘‘endogenously generated’’; for example, the sleep-wake cycle leads to periods of hyperactive motion and increased neural activity alternating with quiescent periods with lower or altered patterns of neural activity (Chiu et al., 2016; Iannacone et al., 2017; Lebestky et al., 2009; Turek et al., 2016). Alternatively, ‘‘exogenously generated’’ or environmentally triggered arousal, in which increased attention and responsiveness are evoked by environmental danger signals (Woods et al., 2014; Yokogawa et al., 2012), is less well understood. Both forms of arousal are found even in simpler organisms. For example, in flies, environmentally triggered arousal appears to involve neuromodulator signaling via tachykinin-related neuropeptides (Asahina et al., 2014) or dopamine, which also modulates sleep-like behavior (Lebestky et al., 2009). Although studies in invertebrates have recently provided important insights into arousal behaviors (e.g., Choi et al., 2015; Laurent et al., 2015; Mahler et al., 2014), in no organism is there a complete picture of how individual neuromodulators act within defined neural circuits to modify behavioral states.

This is an open access article under the CC BY license (http://creativecommons.org/licenses/by/4.0/).

The nematode *C. elegans* provides an excellent system to study the molecular mechanism of sensitization and its contribution to behavioral states such as arousal. *C. elegans* is genetically tractable, is amenable to interrogation of the nervous system at the cellular and whole-organism level, and has a largely complete neuronal connectome. In addition to this "wired" connectome of synapses and gap junctions (White et al., 1986), *C. elegans* makes use of a remarkably complex array of neuromodulators (Hobert, 2013; Walker et al., 2017), most of which are known or thought to function extrasynaptically (Bentley et al., 2016). This "wireless" connectome of neuromodulatory signaling interactions is involved in the regulation of diverse behaviors (Bargmann, 2012). For example, the G-protein-coupled receptor (GPCR) NPR-1 and its ligands FLP-18 and FLP-21 have been implicated in both environmentally triggered arousal (Gray et al., 2004) and sleep/wake transitions (Choi et al., 2013) in *C. elegans*. Likewise, repeated optogenetic stimulation of the ASH neurons triggers increased forward locomotion, a response that requires the neuropeptide receptor *pdfr-1* (Ardiel et al., 2017). In each of these cases, neuromodulators appear to modify behavioral outputs in response to changes in the environment, a role that appears generally conserved among animals (Bargmann, 2012; Marder, 2012; Taghert and Nitabach, 2012; Wester and McBain, 2014). Probing the cellular and molecular effects of neuromodulators on arousal in *C. elegans* may therefore help elucidate general principles through which neuromodulators interact with neural circuits to control complex behavioral states.

Here we describe a new paradigm for arousal in *C. elegans*, evoked in response to mechanosensory stimulation. We find that in addition to an acute escape reflex, an aversive mechanical stimulus leads to increased locomotor activity as well as sensitization of the major nociceptive neuron ASH, an arousal state that persists for 1–2 min. Both locomotor arousal and sensory facilitation require the release of FLP-20 neuropeptides from primary mechanosensory neurons, which act through the same Gα_q-coupled receptor, FRPR-3. These effects involve FRPR-3-dependent activation of the neuroendocrine cell RID; thus, arousal appears to involve a chain of extrasynaptic signaling events acting in parallel to the circuitry of the wired connectome.

RESULTS

C. elegans Displays Both Locomotor and Sensory Arousal in Response to Aversive Stimuli

In many animals, behavioral arousal—including enhanced locomotor activity and facilitation of sensory pathways—often occurs following exposure to aversive stimuli. Indeed, we observed that non-localized mechanosensory stimulation (1× or 5× rapidly applied taps to the animal's Petri dish) led to a prolonged (up to 120 s) increase in forward locomotion speed (Figure 1A). To determine if this was a general effect of exposure to aversive stimuli, we applied various different stimuli, including heat, harsh touch, lifting with a platinum wire pick, and odorants, to wild-type animals and recorded their speed before and after stimulus application (Figure S1A). As was the case for tap stimulation, animals showed a robust and persistent increase in locomotion speed in response to all the aversive stimuli, but not to benzaldehyde, which is an attractive stimulus (Figure S1A). Importantly, the effect of mechanosensory stimulation on locomotion was dose dependent, as increasing the number of taps led to a higher amplitude and longer-lasting period of locomotor arousal (Figures 1A and S1B).

To determine if aroused animals also displayed increased sensory responsiveness, we investigated if behavioral responses to other aversive stimuli were enhanced during mechanically evoked arousal. We expressed the optogenetic actuator channelrhodopsin-2 (ChR2) cell-specifically in the ASH polymodal nociceptor neurons and tested whether the application of an arousing tap stimulus would lead to an enhanced escape response to ASH activation. Indeed, we found that animals receiving a tap stimulus prior to optogenetic activation of ASH exhibited a significantly higher reversal response compared with control animals that did not receive a pre-arousing stimulus (Figures 1B and 1C). Escape responses to repellents sensed by ASH, for example glycerol (Hilliard et al., 2005), were likewise enhanced by prior experience of an arousing mechanical stimulus (Figure S1D). Control animals in which ASH was not optogenetically activated showed reversal responses after tap that were not substantially different from unaroused animals (Figure S1E), indicating that it was the aversive chemosensory pathway, rather than the reverse locomotor pathway per se, that was sensitized. As expected based on previous work (Rose and Rankin, 2001), tap stimulation did not significantly enhance the response to a second tap, indicating that sensitization of the ASH pathway is specific and cross-modal (Figure S1F). Additionally, the presence of benzaldehyde, an appetitive stimulus, did not prevent enhancement of ASH responses by tap, although the magnitude of the enhancement was slightly though not significantly reduced (Figure S1G). Similar to the effects on locomotor arousal, the dosage of the pre-arousing stimulus affected the duration of the sensory sensitization response, as multiple (5×) taps led to longer-lasting enhancement of ASH responses than a single tap (Figure 1B). Together, these results show that mechanosensory arousal not only increases locomotor activity but also sensitizes ASH-dependent sensory pathways linked to avoidance and escape behavior.

In principle, the enhanced escape behavior we observed in mechanically aroused animals could be the result of an increase in sensory neuron activity, or alternatively could result from downstream effects in the neural circuitry. To distinguish these possibilities, we directly measured ASH sensory responses in aroused and unaroused animals. We used genetically encoded calcium indicators to measure ASH calcium levels as a proxy for neuron activity after exposure to the osmotic stressor glycerol alone, or following mechanical stimulation of the anterior body. This experiment was performed using a custom microfluidics chip capable of providing both mechanical stimulation to the worm's anterior body using a pneumatic valve system and chemosensory stimulation to the head of the animal using off-chip solenoid valves (Cho et al., 2017, 2018). Using this system, we found that animals pre-aroused with the mechanical stimulus demonstrated a significantly higher ASH calcium response to

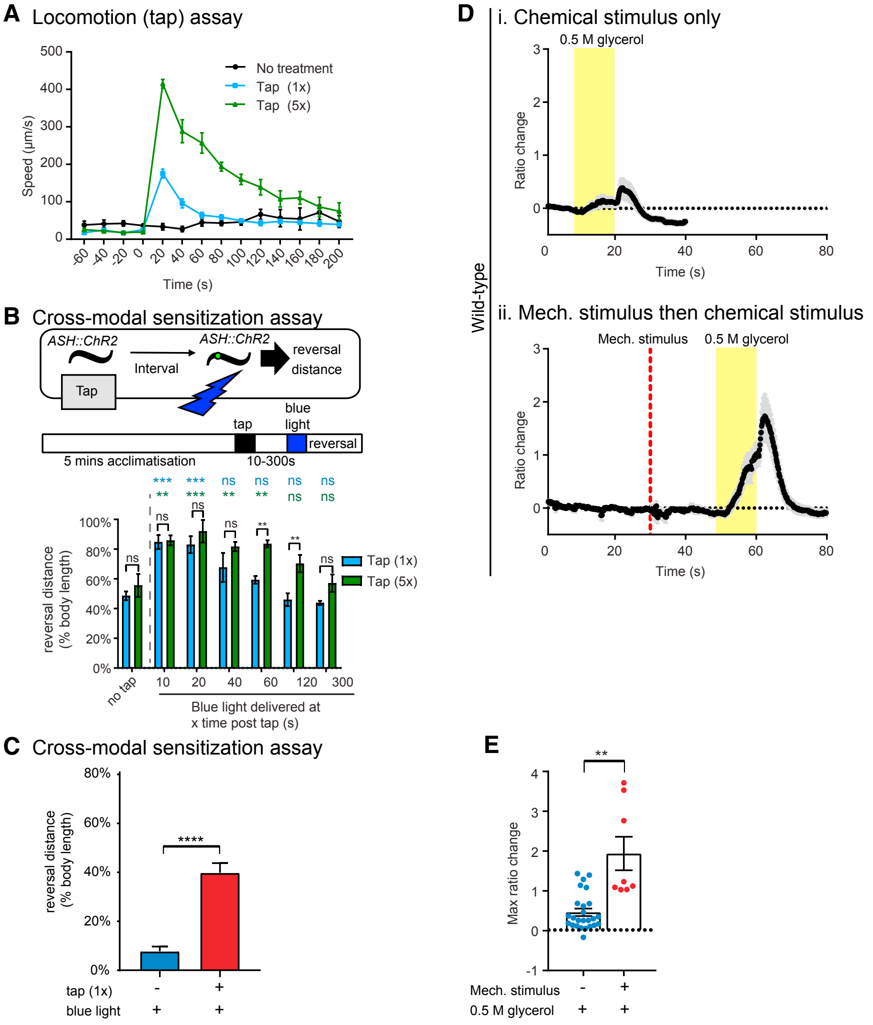

Figure 1. *C. elegans* Enters an Aroused Behavioral State in Response to Aversive Stimuli

(A) Wild-type animals show an increased locomotion speed following the onset of mechanosensory (tap) stimuli. The acute effects of tap (<10 s) are largely due to the initial escape response comprised of backward locomotion (Rankin et al., 1990). Five to ten animals were assayed in at least five trials for each condition.

(B) Reversal distance for pre-aroused animals (1×tap or 5×tap) at various intervals prior to activation of ASH, compared with non-pre-aroused controls. Increased dosage of the pre-arousing stimulus extends the duration of the sensory sensitization effect. p values in black refer to comparisons between 1×tap and 5×tap. p values in blue or green refer to comparisons between 1×tap and no tap (blue) or 5×tap and no tap (green) for each duration tested. n > 4 trials. Two-way ANOVA, Fisher's post-test: ns, not significant; $^{**}p < 0.01$, $^{***}p < 0.001$.

(C) Reversal distance for aroused or unaroused *ASH::ChR2* animals in response to optogenetic activation of ASH 20 s after the pre-arousing tap stimulus. This interval was used for all subsequent experiments. n = 5 trials. Unpaired t test, $^{****}p < 0.0001$.

(D) Mean traces of ASH calcium activity in response to glycerol either (i) alone or (ii) following a mechanical stimulus applied to the body of the animal.

(E) Quantification of calcium activity in the ASH neuron. n = 8–25. Welch's t test, $^{**}p < 0.01$.

For all panels, error bars indicate mean ± SEM. See also Figures S1 and S2.

glycerol compared with animals that did not receive the pre-arousing stimulus (Figures 1D, 1E, and S1H). Together with the finding that arousal enhances the behavioral response to optogenetic ASH stimulation, this result implies that mechanosensory stimulation increases the excitability of the ASH sensory neurons themselves, an effect we refer to as sensory facilitation. Interestingly, prior exposure to the aversive odorant nonanone, which can evoke locomotor arousal (Figure S1A), did not significantly enhance ASH responses to glycerol (Figures S2A and S2B), nor did it enhance behavioral responses to optogenetic activation of ASH (Figure S2C). Thus, ASH chemosensory responses appear to be enhanced specifically and cross-modally by body touch.

The Neuropeptide FLP-20 Is Required for Locomotor and Sensory Arousal

We hypothesized that neuromodulators may be required for entry into the aroused behavioral state in *C. elegans* given the requirement for neuropeptides and neurotransmitters for arousal in other organisms. To identify *C. elegans* neuromodulators that are required for arousal, we performed a candidate screen for mutants that failed to increase their locomotion speed or exhibit sensory facilitation in response to an aversive tap stimulus (Figure S3). We used an automated multiple worm tracker (Ramot et al., 2008) to measure locomotion speed of many (>50–100) animals simultaneously in the minutes following mechanosensory stimulation. Using this method, we found that, compared with wild-type, animals carrying a deletion in the neuropeptide precursor gene *flp-20* showed a reduced magnitude of locomotor speed increase in response to tap (Figures 2A–2C and S3B). However, *flp-20* mutants are able to sense mechanical stimulation, as they exhibited robust reversal behavior in response to gentle touch (which, like tap, is also sensed by the touch receptor neurons [TRNs]) that was not significantly different from wild-type (Figure S2D). Thus, *flp-20* appears to be specifically required for locomotor arousal evoked by mechanosensory stimulation. We next tested if sensory facilitation also requires FLP-20. In the behavioral assay, we observed that *flp-20(ok2964)* deletion mutants, in contrast to control animals, failed to show robust mechanosensory enhancement of the reversal response to optogenetic activation of ASH (Figures 2D and S3C). We also investigated if the increased ASH calcium responses to glycerol following pre-arousing mechanical stimulation required

CellPress

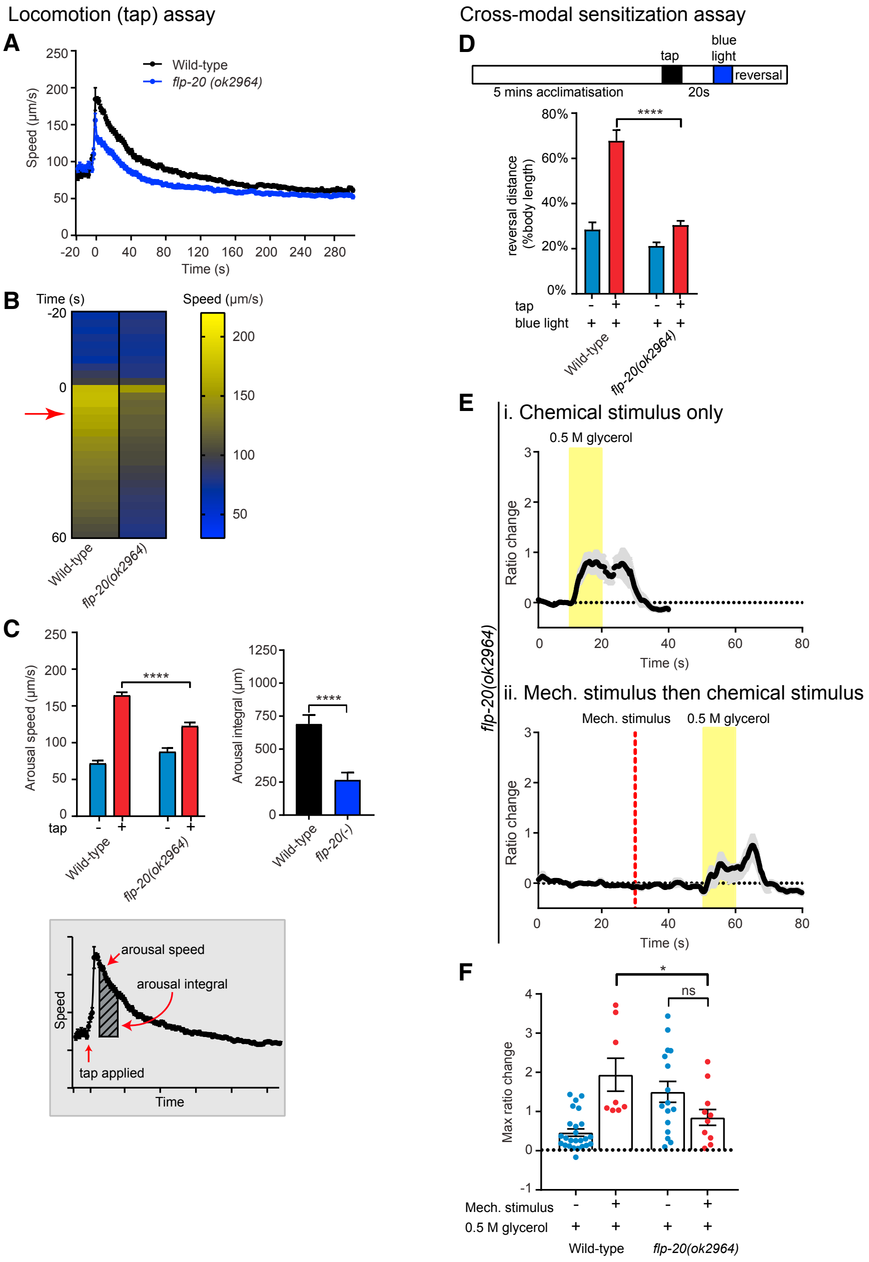

Figure 2. The Neuropeptide FLP-20 Is Required for Locomotor and Sensory Arousal

(A–C) Locomotion arousal assays: (A) The speed of *flp-20* mutant animals compared with wild-type, following a tap stimulus, as shown in the speed trace indicating speed (μm/s) for 5 min. Tap stimulus is applied at t = 0. (B) shows a heatmap indicating the mean speed for all replicates. Only the first 60 s post-tap is shown. The red arrow indicates the time point at which the arousal speed is determined. (C) shows the quantification of arousal speed and the arousal integral. The diagram below shows how the arousal speed and arousal integral are calculated (see STAR Methods for details). Error bars indicate mean ± SEM. Unpaired t test: ****$p < 0.0001$. For (A)–(C), $n > 200$ for at least three trials.

(D–F) Cross-modal sensitization assays: (D) Reversal responses to blue light stimulation for wild-type and *flp-20* mutants expressing the *ASH::ChR2* transgene, with or without a pre-arousing tap. n = 5 trials. Unpaired t test: ****$p < 0.0001$. (E) Mean traces of ASH calcium activity in *flp-20* mutant animals measured with GCaMP3 in response to 0.5 M glycerol either (i) alone or (ii) following a mechanical stimulus applied to the body of the animal. n = 12–16. (F) Quantification of calcium activity in the ASH neuron of wild-type and *flp-20* mutant animals. Welch's t test: ns, not significant; *$p < 0.05$. For (D)–(F), error bars indicate mean ± SEM. See also Figures S2 and S3.

FLP-20. Again we found that *flp-20* mutant animals showed no significant enhancement of ASH chemosensory responses to glycerol by a prior mechanical arousing stimulus (Figures 2E and 2F). Taken together, these findings indicate that FLP-20 is required for both locomotor arousal and cross-modal sensitization following mechanical stimulation.

To determine where FLP-20 peptides are required for arousal, we assayed for phenotypic rescue under cell-type-specific promoters. We first generated a transgenic line expressing mKate2 under the control of the *flp-20* promoter (P*flp-20::flp-20 gDNA + 3′ UTR::SL2-mKate2*) to determine the expression pattern of *flp-20*. Consistent with previous findings (Kim and Li, 2004), we found that *flp-20* is expressed in the TRNs, along with a few other neurons (ASE, LUA, and PVC) (Figure 3A). Since the TRNs are known to be activated in response to tap stimuli, we next tested if re-expression of FLP-20 in the TRNs (using P*mec-4*) was able to rescue the defects observed in *flp-20* mutants. Indeed, this *TRN::flp-20* transgene was able to rescue locomotor arousal in *flp-20(ok2964)* mutant animals (Figures 3B–3D). Likewise, we observed that TRN-specific expression of *flp-20* rescued the cross-modal sensitization phenotype when measured either by behavioral response to optogenetic ASH activation (Figure 3E) or by calcium imaging of ASH chemosensory responses (Figures 3F and 3G). These results indicate that FLP-20 functions in the TRNs to promote multiple arousal pathways and imply that FLP-20 peptides are released directly from the TRNs following mechanical stimulation.

FRPR-3 Is a Receptor for FLP-20 Peptides that Mediates Arousal

To understand the neural mechanism by which *flp-20* triggers arousal, we first sought to identify the receptor for FLP-20 peptides. We expressed 79 candidate neuropeptide receptors from the *C. elegans* genome in mammalian cells expressing aequorin

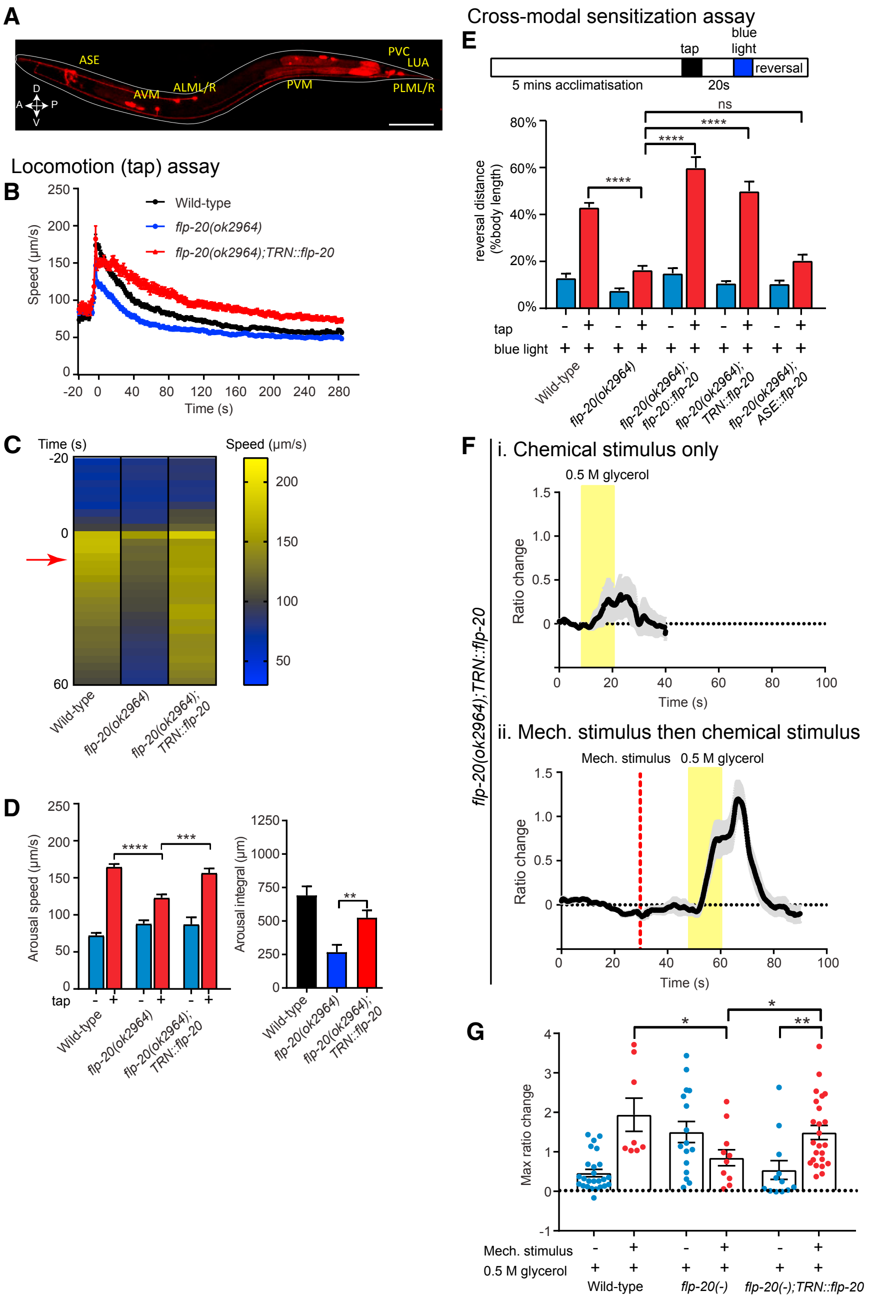

Figure 3. FLP-20 Neuropeptide Release from Touch Neurons Is Required for Arousal

(A) A reporter line expressing the transgene *Pflp-20::flp-20 genomic DNA::SL2-mKate2* shows expression in the gentle touch neurons (TRNs: ALM, AVM, PLM, PVM) as well as ASE, LUA and PVC. Scale bar indicates 100 μm.

(B–D) Locomotion arousal assays: (B) Expression of FLP-20 in the TRNs (P*mec-4*) in a *flp-20* mutant rescues the defect in locomotor arousal observed in *flp-20* mutant animals. (B) shows the speed trace for wild-type, *flp-20* mutants, and the TRN rescue line. The tap stimulus is applied at t = 0. (C) shows a heatmap indicating the mean speed for all replicates of each genotype tested. The red arrow indicates the time point at which the arousal speed is determined. (D) shows the quantification of arousal speed and the arousal integral after tap. n > 200 for at least three trials. Error bars indicate mean ± SEM. One-way ANOVA, Sidak's post-test: ns, not significant; **$p < 0.01$, ***$p < 0.001$, ****$p < 0.0001$.

(E–G) Cross-modal sensitization assays: (E) Reversal responses following blue light stimulation, with or without a pre-arousing tap, for wild-type, *flp-20* mutants, and rescue lines expressing FLP-20 using its own promoter (*flp-20::flp-20)* or using a TRN-specific promoter *mec-4* (*TRN::flp-20*). A control transgenic line re-expressing *flp-20* in ASE neurons (P*gcy-5*/P*gcy-7*) did not show significant rescue compared with *flp-20* mutants. One-way ANOVA, Sidak's post-test: ns, not significant, ****$p < 0.0001$. n = 3–6 trials. (F) Mean traces of ASH calcium activity in transgenic animals expressing *flp-20* in the TRNs after exposure to 0.5 M glycerol either (i) alone or (ii) following a mechanical stimulus applied to the body of the animal. n = 12–23. (G) Quantification of calcium activity in the ASH neuron for wild-type, *flp-20(ok2964)*, and *flp-20(ok2964);TRN::flp-20* animals. Welch's t test, *$p < 0.05$, **$p < 0.01$. For (E)–(G), error bars indicate mean ± SEM.

and the promiscuous G-protein Gα_{16}, and assayed each for calcium responses to the peptides encoded by *flp-20*. We found that all three peptides encoded by the *flp-20* gene (AMMRFa, AVFRMa, SVFRLa) were able to activate one of these receptors, the FMRFamide-like peptide GPCR FRPR-3, with EC_{50} values in the low nanomolar range (Figure 4A). Interestingly, when *frpr-3* was expressed in cells expressing aequorin but lacking Gα_{16}, each of the FLP-20 peptides still robustly evoked calcium transients, suggesting that FRPR-3 acts through a calcium-mobilizing second-messenger pathway such as Gα_q/PLCβ (Figure S4A). Phylogenetic analysis indicates that FRPR-3 belongs to the RFamide neuropeptide receptor family and is evolutionarily related to the *Drosophila* FMRFa receptor (Dmel\FMRFaR) (Elphick and Mirabeau, 2014).

We next investigated whether the arousal phenotypes of *flp-20* depend on *frpr-3*. We obtained a deletion mutant of *frpr-3* and assayed it for locomotor and sensory arousal following mechanosensory stimulation. Similar to *flp-20(ok2964)* mutants, *frpr-3(ok3302)* mutant animals failed to significantly increase their speed in response to a tap stimulus (Figures 4B and 4C). Double-mutant animals containing both *flp-20* and *frpr-3* deletion alleles were arousal defective to a level similar to that of single mutants, suggesting that FRPR-3 and FLP-20 peptides act in the same pathway *in vivo* (Figure 4C). We were able to rescue locomotor arousal in *frpr-3* and *flp-20* mutants by re-expressing *frpr-3* and *flp-20*, respectively, under the control of the endogenous promoters for these genes (Figures 4D–4F and S4B). We also found that the rescuing effect

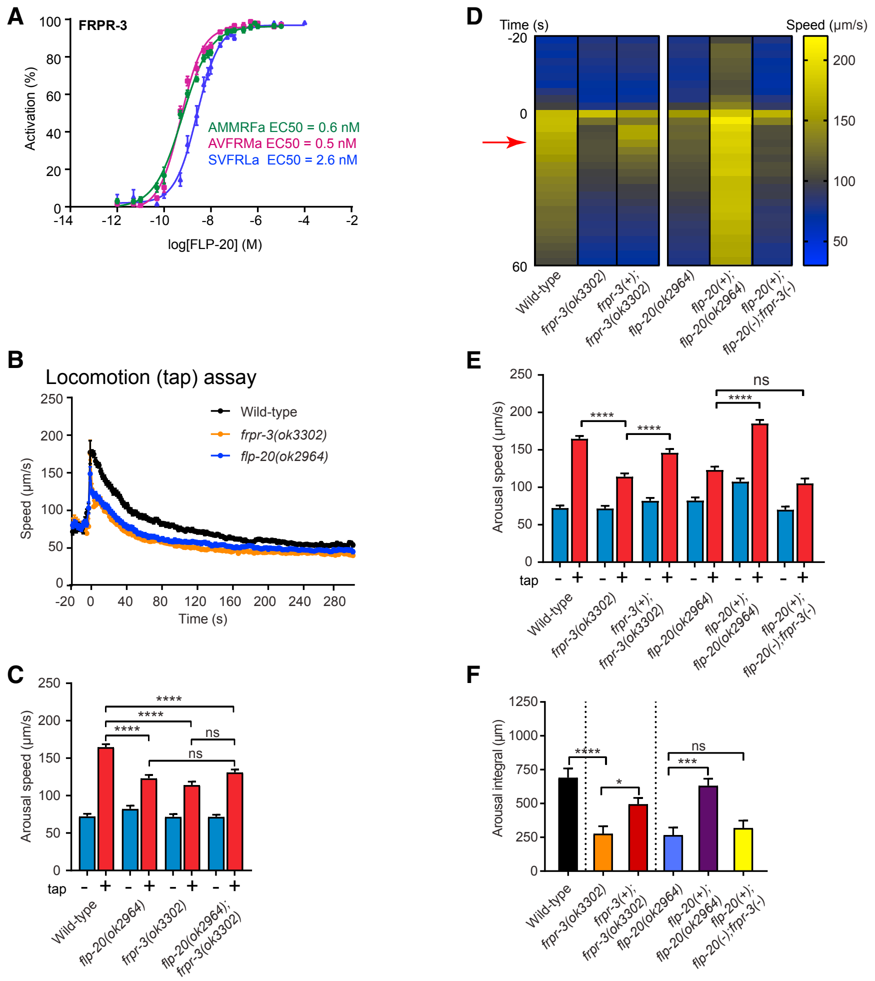

Figure 4. The FLP-20 Receptor FRPR-3 Is Required for Locomotor Arousal

(A) Dose-response curve showing that peptides encoded by the FLP-20 precursor activate the RFamide receptor FRPR-3 *in vitro*. The corresponding EC_{50} values for each peptide are as indicated. Line represents non-linear regression fit of a variable slope line using 4 parameters. n = 6–8 trials.

(B–F) Locomotion arousal assays: (B) *frpr-3* deletion mutants show a smaller speed increase compared with wild-type following a tap stimulus, as shown in the speed trace. The tap stimulus is applied at t = 0. (C) Quantification of arousal speed indicating that *flp-20* and *frpr-3* single and double mutants are defective in locomotor arousal after tap. (D and E) The defect in tap arousal observed in single *frpr-3* and *flp-20* deletion mutant animals can be rescued by expression of FRPR-3 (*frpr-3(+)*) and FLP-20 (*flp-20(+)*) gDNA, respectively, under the control of the endogenous promoter for each gene. Double mutants of *flp-20* and *frpr-3* expressing the *flp-20(+)* transgene show decreased locomotor arousal after tap. (D) shows a heatmap indicating the mean speed for all replicates of each genotype tested. The red arrow indicates the time point at which the arousal speed is determined. (E) shows the quantification of arousal speed for all genotypes. (F) shows the quantification of the arousal integral. One-way ANOVA, Sidak's post-test: ns, not significant; *p < 0.05, ***p < 0.001, ****p < 0.0001. Error bars indicate mean ± SEM. For (B)–(F), n > 200 for at least three trials. See also Figure S4.

of the *flp-20(+)* transgene was dependent on FRPR-3, as *flp-20(+);flp-20(ok2964)* mutant animals also containing the *frpr-3(ok3302)* allele failed to increase their speed in response to tap (Figures 4D–4F). Together, these findings demonstrate that the effect of FLP-20 on arousal is FRPR-3 dependent, supporting the hypothesis that FRPR-3 is the receptor for FLP-20 peptides *in vivo* that mediates their effects on locomotor arousal.

We next tested if FRPR-3 was required to mediate other effects of FLP-20 peptides on behavior. Similar to our observations in *flp-20* mutant animals, we found that *frpr-3* mutants show a significantly reduced enhancement of the behavioral response to optogenetic ASH activation by an arousing tap stimulus (Figure 5A). Consistent with this effect on behavior, animals lacking *frpr-3* also did not show increased ASH neuron activity when stimulated with glycerol following a pre-arousing mechanical stimulus to the body (Figures 5B and 5C). In a previous study, we also showed that FLP-20 modulates responses to appetitive olfactory cues, leading to altered chemotaxis and an increased reversal rate off food in *flp-20* mutants (Rabinowitch et al., 2016a). This suggested that tonic FLP-20 signaling suppresses attractive responses. We observed that *frpr-3* mutants and *flp-20;frpr-3* double mutants exhibited this phenotype as well (Figure S4C), consistent with FRPR-3 acting as the receptor for FLP-20 peptides in this behavior. Thus, FRPR-3 also appears to be the receptor that mediates other effects of FLP-20 peptides.

FRPR-3 Acts in the RID Neuron for Locomotor Arousal and Cross-modal Sensitization

We next investigated which FRPR-3-expressing cell(s) is required for arousal. We generated a transgenic line expressing mKate2 under the control of the *frpr-3* promoter (P*frpr-3::frpr-3 genomic DNA + 3′ UTR::SL2-mKate2*) to determine the expression pattern of this receptor. We found that FRPR-3 is expressed mainly in a few head neurons, including RID, ASK, AIY, and AVK, consistent with previous expression data (Turek et al., 2016) (Figure 6A). We were intrigued by FRPR-3 expression in RID, as this interneuron was recently shown to be required for sustaining the forward motor state (Lim et al., 2016). As of yet, no RID-specific promoter has been identified. To determine if FRPR-3 in RID is required for tap arousal, we tested if expressing a transgene for *frpr-3* using three different promoters driving expression in RID as well as other neurons could rescue the defect in locomotor arousal observed in *frpr-3* mutant animals. Indeed, we found that expression of FRPR-3 using either the *flp-2* (Kim and Li, 2004), *des-2* (Van Buskirk and Sternberg, 2010), or *ceh-10*

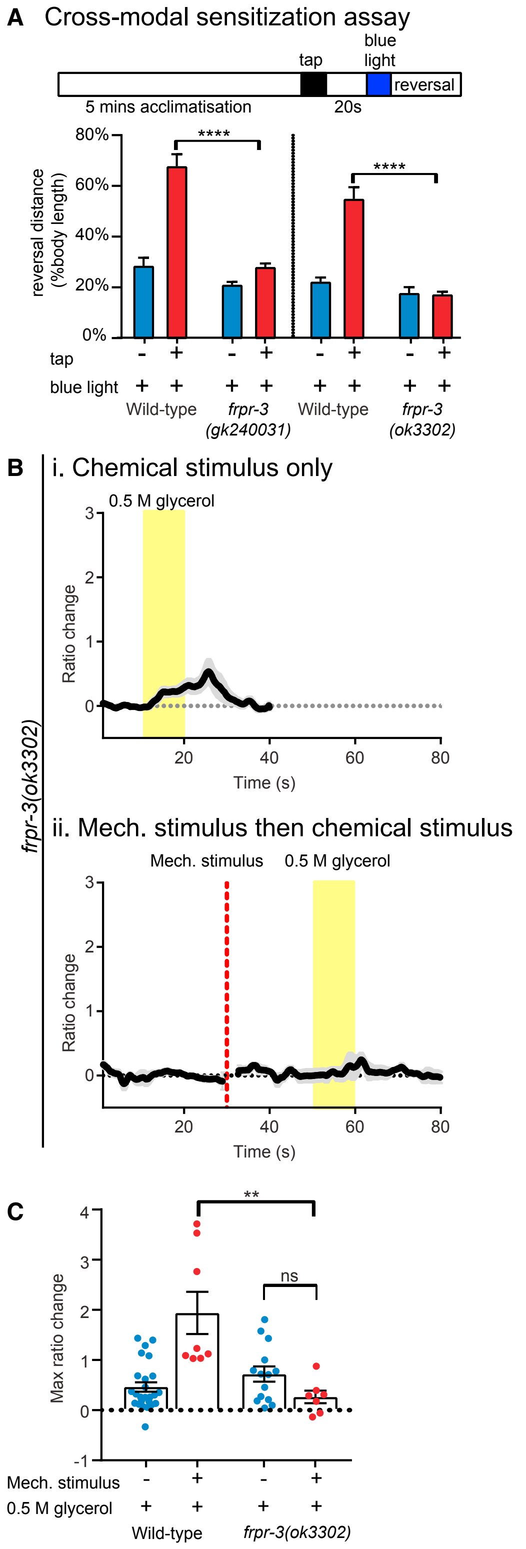

Figure 5. FRPR-3 Is Required for Sensory Sensitization

(A) Reversal responses for wild-type and *frpr-3* mutants expressing the *ASH::ChR2* transgene following blue light stimulation, with or without a pre-arousing tap. n > 8 trials. Unpaired t test, ****$p < 0.0001$.

(B) Mean traces of ASH calcium activity in *frpr-3* mutant animals in response to 0.5 M glycerol either (i) alone or (ii) following a mechanical stimulus applied to the body of the animal, indicating a defect in sensory arousal in these mutant strains. n = 5–15.

(C) Quantification of calcium activity in the ASH neuron of wild-type and *frpr-3* mutant animals. Welch's t test: ns, not significant; **$p < 0.01$. Error bars indicate mean ± SEM. See also Figure S4.

promoter (Altun-Gultekin et al., 2001; Lim et al., 2016) all showed significantly increased speed induced by tap compared with *frpr-3* mutant animals (Figures 6B–6D and S5A). We also found that re-expression of *frpr-3* in AIY (using P*ttx-3*) was able to significantly rescue the defect in locomotor arousal observed in *frpr-3* mutants (Figures S5A–S5C). In contrast, a transgene expressing *frpr-3* in the AVK (using P*flp-1*) or ASK neurons (using P*sra-9*) failed to rescue the locomotor arousal defect (Figures 6B–6D and S5A–S5C).

We also investigated where *frpr-3* was required for cross-modal sensitization. To address this question, we knocked down *frpr-3* expression in different *frpr-3*-expressing neurons using cell-specific RNAi. We knocked down expression in RID by expressing antisense and sense sequences of *frpr-3* with a promoter combination (P*flp-2* and P*des-2*) that overlap in RID. We found that knockdown of *frpr-3* in RID using this intersectional promoter strategy led to a significant decrease in ASH-driven reversal responses following a pre-arousing tap stimulus (Figures 6E and S6B). We also tested knockdown of *frpr-3* using other promoter combinations that appear to solely overlap in RID (Figures S6A and S6B). Knockdown of *frpr-3* in two of these three additional lines showed a significant decrease in ASH sensitization compared with controls (Figure S6B). In contrast, knockdown of *frpr-3* in ASK, which is connected by gap junctions to ASH, or in ASH itself, had no significant effect on sensory sensitization (Figure S5D). Consistent with these behavioral results, we found that transgenic re-expression of FRPR-3 in RID using both the *des-2* and *flp-2* promoters (Figures 6F, 6G, and S5E) conferred significant rescue of the ASH sensitization phenotype as measured by calcium imaging of ASH chemosensory responses. Interestingly, although AIY expression of *frpr-3* appears to modulate locomotor arousal (Figures S5A–S5C), knockdown of *frpr-3* in AIY had no effect on cross-modal sensitization (Figure S5F). Additionally, knockdown of *frpr-3* in AVK, in which *frpr-3* is strongly expressed (Figure 6A), had no effect on ASH sensory responses (Figure S5F). Altogether, our data are consistent with FRPR-3 mediating the effects of FLP-20 peptides on both locomotor and sensory arousal through its effects on the RID neuron.

FLP-20 and FRPR-3 Facilitate Enhanced RID Activity in Response to Mechanical Stimuli

To further investigate the role of the RID neuron in tap-mediated arousal, we recorded neuronal activity in RID in response to mechanical stimulation. We applied a computer-controlled mechanosensory stimulus to the posterior half of the body to activate the TRNs and simultaneously measured RID calcium responses using the sensor Cameleon (YC3.60). In wild-type animals, there was a robust increase in calcium transients in RID in response to

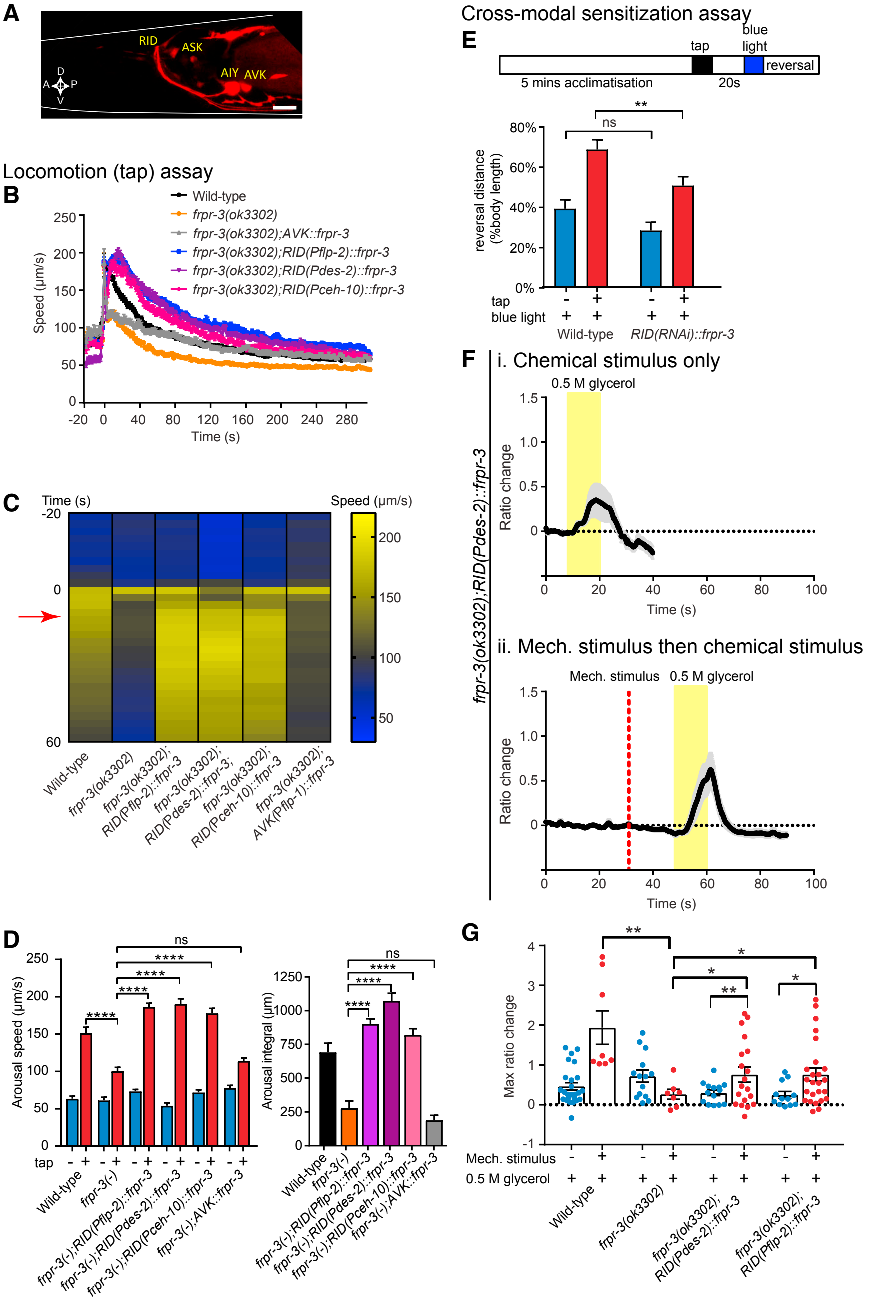

Figure 6. FRPR-3 Acts in the RID Neuron for Locomotor Arousal and Sensory Sensitization

(A) A reporter line expressing the transgene *Pfrpr-3::frpr-3 genomic DNA::SL2-mKate2* shows expression mostly in head neurons including RID, ASK, AIY, and AVK. Scale bar indicates 10 μm.

(B–D) Locomotion arousal assays: Re-expression of *frpr-3* in RID (and other neurons) using the *flp-2*, *des-2*, and *ceh-10* promoters rescued the defect in tap arousal in *frpr-3* mutants, whereas transgenic re-expression of *frpr-3* in AVK using the *flp-1* promoter failed to show significant rescue. (B) shows the speed trace for all genotypes. The tap stimulus is applied at t = 0. (C) shows a heatmap indicating the mean speed for all replicates of each genotype tested. The tap stimulus is applied at t = 0. The red arrow indicates the time point at which the arousal speed is determined. (D) shows the quantification of arousal speed and the arousal integral after tap. n > 200 for at least three trials. For (B) and (D), error bars indicate mean ± SEM. One-way ANOVA, Sidak's post-test: ns, not significant; ****$p < 0.0001$.

(E–G) Cross-modal sensitization assays: (E) Reversal responses for wild-type and *RID (RNAi)::frpr-3* animals following blue light stimulation, with or without a pre-arousing tap. RNAi transgenes were expressed using P*flp-2* and P*des-2*. Two-way ANOVA, Fisher's post-test: ns, not significant; **$p < 0.01$. n > 6 trials. (F) Mean traces of ASH calcium activity in *frpr-3(ok3302);RID(Pdes-2)::frpr-3* transgenic animals in response to glycerol either (i) alone or (ii) following a mechanical stimulus applied to the body of the animal. n = 12–25. (G) Quantification of calcium activity in the ASH neuron for wild-type, *frpr-3(ok3302)*, *frpr-3(ok3302);RID(Pdes-2)::frpr-3*, and *frpr-3(ok3302);RID(Pflp-2)::frpr-3* transgenic animals. For (E)–(G), error bars indicate mean ± SEM. Welch's t test, *$p < 0.05$, **$p < 0.01$. See also Figures S5 and S6.

mechanical stimulation (Figure 7A). In contrast, this response was significantly diminished in *frpr-3* mutants and was almost completely abolished in *flp-20* mutant animals (Figures 7A and 7B). We found that wild-type animals showed positive responses as determined by the observation of calcium transients in 79.% ± 1.0% (mean ± SEM) of stimulations, whereas *frpr-3* and *flp-20* mutants showed 38.2% ± 4.3% and 11.2% ± 1.8% positive responses, respectively (Figure 7C). Consistent with the requirement for FRPR-3 in RID for locomotor arousal behavior, we could rescue the defective RID neural activity in *frpr-3* mutants by re-expressing *frpr-3* in RID and in *flp-20* mutants by re-expressing *flp-20* in the TRNs (Figures 7B, 7C, and S7A). Cell-specific transgenic re-expression of these genes could also rescue the proportion of neurons responding to mechanical stimulation to 83.1% ± 4.8% in *frpr-3(ok3302);RID::frpr-3* animals and 76.4% ± 5.8% in *flp-20 (ok2964);TRN::flp-20* transgenic animals (Figure 7C). These data are consistent with FLP-20 peptides released from the TRNs being required for arousal by activating FRPR-3 receptors in RID.

Is activation of RID sufficient for arousal? A previous study demonstrated that optogenetic activation of RID using Chrimson was sufficient to potentiate the speed of forward locomotion (Lim et al., 2016), thus mimicking the effect of locomotor arousal. To determine whether RID activation is also sufficient to facilitate ASH-mediated escape behavior, we crossed the *RID::Chrimson* transgenic line from the (Lim et al., 2016) study with our

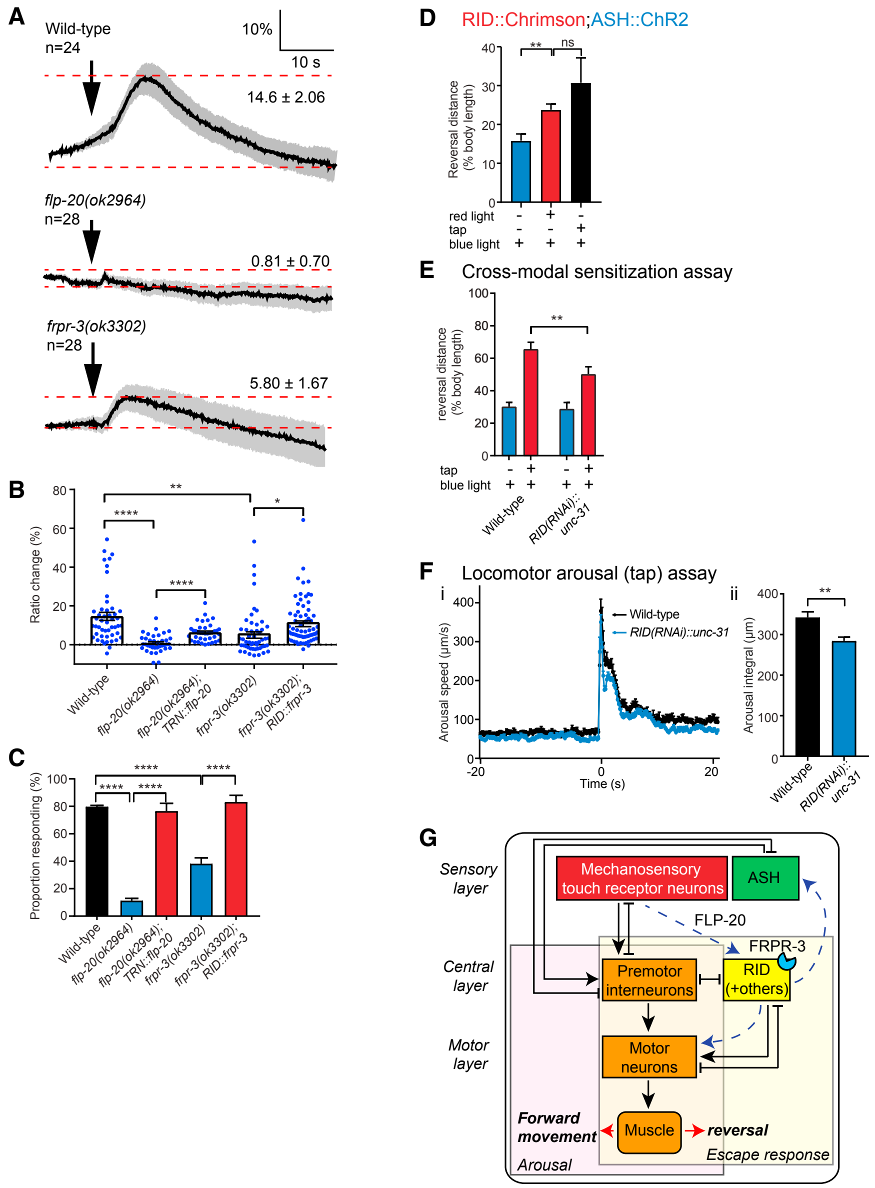

Figure 7. FLP-20/FRPR-3 Facilitates Enhancement of RID Activity in Response to Mechanical Stimuli

(A) Mean calcium traces for wild-type and *flp-20* or *frpr-3* mutant animals in RID following mechanosensory stimulation. The maximum ratio change is shown as mean ± SEM for each trace. The number of worms tested is shown, with each worm stimulated one to three times. Scale shows % ratio change on the y axis and time (s) on the x axis.

(B) Quantification of calcium activity in the ASH neuron plotted as the maximum ratio of YFP/CFP as a percentage of the baseline YFP/CFP ratio. Welch's t test: *p < 0.05, **p < 0.01, ****p < 0.0001. n (traces) = 38–65.

(C) Proportion of trials showing a positive response to mechanical stimulation based on the observation of calcium transients in RID. One-way ANOVA, Fisher's post-test: ****p < 0.0001.

(D) Optogenetic activation of RID with red light prior to activation of ASH using blue light leads to an increased reversal distance compared with animals stimulated with blue light alone. As a positive control, animals of the same genotype were provided with a tap stimulus prior to ASH activation. n = 5 trials.

(E) Reversal responses for wild-type, *RID(RNAi)::unc-31* and animals following blue light stimulation, with or without a pre-arousing tap. Two-way ANOVA, Fisher's post-test: **p < 0.01.

(F) (i) Speed traces and (ii) arousal integral for *RID(RNAi)::unc-31* and control animals after a 1x tap stimulus at t = 0. For (E) and (F), RNAi transgenes were expressed using P *flp-2* and P*des-2*. Unpaired t test: **p < 0.01. n > 6 trials. Error bars indicate mean ± SEM.

(G) In response to mechanical stimulation, FLP-20 is released from the mechanosensory TRNs and drives behavioral arousal via its receptor FRPR-3 acting primarily in RID. FLP-20/FRPR-3 signaling sensitizes the ASH neuron, at least partially via neuropeptides. FLP-20-dependent activation of FRPR-3 in RID enhances locomotion through peptide neuromodulation and perhaps synaptic and/or gap-junction signaling. Mechanosensory neurons also trigger escape behavior through wired connections to premotor interneurons. See also Figure S7.

ASH::ChR2 transgenic line and tested whether prior optogenetic activation of RID could enhance the response to ASH activation. We indeed observed significant sensitization of ASH-mediated escape behavior in this assay, suggesting that increased RID activity could account for both aspects of FLP-20-mediated mechanosensory arousal (Figure 7D).

RID is a specialized neuroendocrine cell (Lim et al., 2016) that contains no known classical neurotransmitters or monoamines (Pereira et al., 2015); however, it expresses a number of neuropeptides (Janssen et al., 2009; Kim and Li, 2004; Lim et al., 2016) as well as making gap junctions with a number of interneurons (White et al., 1986). We therefore hypothesized that RID activity might promote ASH sensitization and locomotor arousal through release of peptide neuromodulators. As a first step to investigate this possibility, we used RNAi to selectively knock down *unc-31*, a CAPS protein specifically required for dense core vesicle release (Speese et al., 2007), in the RID neuron. We observed that these *RID(RNAi)::unc-31* animals indeed showed significant defects in both ASH sensitization (Figure 7E) and locomotor arousal (Figure 7F) in response to mechanosensory stimulation. Interestingly, RID-specific knockdown of *egl-21*, which encodes an enzyme required for maturation of many *C. elegans* neuropeptides (Jacob and Kaplan, 2003), did not significantly affect ASH sensitization (Figure S7B) but resulted in a small yet significant reduction in locomotor arousal (Figure S7C). Thus, RID may use EGL-21-independent neuropeptides (Husson et al., 2007; Van Bael et al., 2018) to modulate peripheral sensory targets during arousal whereas EGL-21-processed neuropeptides may play a larger role in signaling to downstream motor circuits.

DISCUSSION

We have described a novel paradigm for behavioral arousal in *C. elegans* and have characterized key aspects of its neural and molecular mechanism. Mechanosensory stimulation leads to an increase in locomotor speed that persists for minutes, as well as cross-modal sensitization of nociceptor neurons on a similar timescale. Both locomotor and sensory arousal behaviors require FLP-20 neuropeptides, released directly from primary mechanosensory neurons, and their receptor FRPR-3. FRPR-3 in turn modulates the activity of the neuroendocrine cell RID, which becomes activated in response to mechanical stimulation in a FLP-20 and FRPR-3-dependent manner (Figure 7G). RID activation is sufficient for both locomotor arousal and cross-modal sensitization, and both processes depend at least in part on dense core vesicle release from RID. Thus, tap-evoked arousal may involve a cascade of neuropeptide signaling, with FLP-20 conveying mechanosensory information from sensory neurons to RID, and efferent neuromodulators conveying behavioral state information from RID to peripheral sensory and motor circuits.

The cellular mechanisms underlying sensitization have been studied for several decades, beginning with work in *Aplysia* (Carew et al., 1971) and more recently in genetically-tractable organisms such as *C. elegans* (Ardiel et al., 2017; Chen and Chalfie, 2014; Choi et al., 2015), but important questions remain. First, does sensitization involve effects on the sensory organ itself, or in the integration and gating of sensory signals? Studies in *Drosophila* suggest that some of the changes in sensory sensitivity during normally quiescent periods may be due to the activity of "clock" neurons that regulate circadian rhythms rather than direct effects on sensory neurons (van Swinderen and Andretic, 2003). Our findings suggest that, in the case of FLP-20 and FRPR-3, changes in responsiveness to an aversive chemical following arousal result from increased sensitivity of the sensory neuron ASH itself and not in downstream components of the circuit. Second, during arousal, is sensitization restricted to particular sensory modalities or is there a generalized state of increased sensory responsiveness? In zebrafish, increasing orexin/hypocretin levels specifically affect responsiveness to dark-flash visual stimuli, but not to acoustic or thermal stimuli (Woods et al., 2014). In another study, arousal induced by water-flow leads to elevated locomotion and increased sensitivity to visual motion but not light-flash or acoustic stimuli (Yokogawa et al., 2012). These studies suggest that arousal in response to a particular stimulus results in increased responsiveness to only particular "goal-directed" sensory modalities. This also appears to be the case for FLP-20-dependent modulation (see below).

Although this study is the first to describe a cellular mechanism for arousal in response to an acute noxious stimulus, the influence of a stressful environment (for example, starvation) on sensitization and/or locomotor arousal has been described in other contexts. For example, the neurotransmitters dopamine (Ezcurra et al., 2011; Ezcurra et al., 2016) and serotonin (Chao et al., 2004; Harris et al., 2009) act through multiple receptors to enhance ASH chemical avoidance responses in the presence of food. This is analogous to observations in mammals where satiety or hunger also alters the sensory responsiveness to olfactory cues (Aime et al., 2007; Ramaekers et al., 2016). Wild *C. elegans* strains also show increased locomotor activity in the presence of high oxygen (Laurent et al., 2015); although the trigger for this arousal is not well understood, a neuropeptide signaling pathway involving the NPR-1 receptor and the FLP-18 and FLP-21 peptides has been shown to negatively regulate this response (Gray et al., 2004; Rogers et al., 2003). Heightened locomotor activity in unfavorable conditions has also been observed in zebrafish (Curtright et al., 2015; Prober et al., 2008) and flies (Lebestky et al., 2009).

For both locomotor arousal and cross-modal sensitization, a critical cellular site of FLP-20/FRPR-3 signaling is the RID neuron (Figures 6 and 7). RID becomes activated in response to mechanosensory stimuli in a FLP-20 and FRPR-3 dependent manner (Figure 7), and cell-specific rescue and/or knockdown experiments identify RID as a focus of both the locomotor arousal and sensitization phenotypes of *frpr-3*. We also found that optogenetically activating RID is sufficient for cross-modal sensitization of ASH (Figure 7D). Interestingly, although loss of *flp-20* completely eliminated RID responses to body touch, detectable RID touch responses remained in the *frpr-3* mutant (Figures 7A and 7B); thus, some FLP-20-mediated effects on RID may be independent of FRPR-3. In our study, FRPR-3 was the only high-potency receptor identified, but the possibility remains that FLP-20 peptides activate other, as-yet-unidentified receptors. Moreover, we cannot rule out the possibility that other cells may represent biologically relevant targets of FLP-20 signaling during arousal. In particular, we found that *frpr-3* expression in AIY affected locomotor arousal (Figures S5A–S5C), though not cross-modal sensitization of ASH (Figure S5F). AIY has previously been shown to promote increased locomotion speed via excitatory synapses with RIB (Li et al., 2014), as well as through gap junctions with RIM (Gray et al., 2005). Interestingly, RIM controls locomotion at least in part through release of tyramine (Alkema et al., 2005; Donnelly et al., 2013; Pirri et al., 2009), and our initial candidate screen identified the tyramine receptors SER-2 and TYRA-2 as potential modulators of tap-evoked locomotor arousal (Figure S3B), but not cross-modal sensitization (Figure S3C).

RID is one of a small number of *C. elegans* neurons that express no classical neurotransmitter or monoamine (Pereira et al., 2015), implying that they may be specialized for neuroendocrine function. While most *C. elegans* neurons contain modest numbers of dense core vesicles, generally peripheral to chemical synapses, RID signals predominantly through large varicosities that contain almost exclusively dense core vesicles (Lim et al., 2016). Both transgenic reporter and expression profiling studies reveal expression of multiple neuropeptide precursor genes in RID (Janssen et al., 2009; Kim and Li, 2004; Lim et al., 2016), including several previously linked to locomotor arousal (Chen et al., 2016). RID makes a few chemical and electrical synapses with motorneurons and body muscle, but much of its effect on locomotion appears to be through neuroendocrine actions on the somatic neuromusculature (Lim et al., 2016; White et al., 1986). Likewise, RID and ASH neurons are not physically connected via synapses or gap junctions, suggesting that communication between RID and ASH could occur most directly via extrasynaptic neuromodulation. We have shown here that *unc-31* knockdown in RID significantly reduces ASH sensitization in

response to tap (Figure 7E), indicating that cross-modal sensitization involves the release of neuromodulators from dense core vesicles in RID. Perhaps surprisingly, RID knockdown of *egl-21*, which encodes an enzyme required for processing many *C. elegans* neuropeptides (Jacob and Kaplan, 2003), did not significantly affect ASH sensitization (Figure S7B), although it did affect locomotor arousal (Figure S7C). Mass spectrometry analysis of *egl-21* mutants indicates that a significant number of *C. elegans* neuropeptides do not require EGL-21 for their processing (Husson et al., 2007). These peptides may be processed by two other putative carboxypeptidases, *cpd-1* and *cpd-2*, which have not been comprehensively characterized (Jacob and Kaplan, 2003; Li and Kim, 2014).

While RID-released peptides seem to promote arousal in a variety of contexts, FLP-20 peptides released from the TRNs appear to specifically encode mechanosensory information. We have shown here that strong acute activation of the TRNs leads to transient FLP-20/FRPR-3-dependent modulation of RID, which in turn promotes locomotor arousal and sensitization of aversive chemosensation. In contrast, our previous work showed that tonic FLP-20 signaling from the TRNs inhibits the acuity of attractive olfactory pathways via suppression of the interneuron AIY's responses to the odorant benzaldehyde. Consequently, in touch-insensitive mutants where this tonic mechanosensory activity was lost, olfactory acuity was sensitized due to a loss of FLP-20 signaling (Rabinowitch et al., 2016a). We showed in this study that this effect was also dependent on FRPR-3 (Figure S4C). Similarly, intermediate-term memory of tap habituation following massed training has been shown to require FLP-20 peptides released from the TRNs (Li et al., 2013). This indicates that FLP-20 signaling acts to inhibit or stabilize the inhibition of mechanosensory responsiveness. Thus, the role of FLP-20 in modulating different sensory modalities appears to depend on context; for aversive chemosensation, FLP-20 peptides enhance sensitivity during acute touch-evoked arousal, whereas for appetitive chemosensation or gentle body touch itself, they diminish sensitivity in response to tonic or prolonged mechanosensory activity. FLP-20 peptides therefore do not function as generalized arousal signals, leading to a general increase in sensory responsiveness, but rather provide mechanosensory information to central neurons on a neuromodulatory timescale. Hence, FLP-20 peptides can be considered afferent neuromodulators that convey sensory information rather than efferent modulators that control peripheral targets.

We have shown that arousal following touch stimulation may involve a sequence of extrasynaptic neuromodulatory signals beginning with a FLP-20/FRPR-3-mediated signal conveying mechanosensory activity to RID. A signal involving multiple arousal neuropeptides released from RID has previously been shown to promote enhanced locomotor activity (Chen et al., 2016; Lim et al., 2016), consistent with data from our study showing that RID peptides are required for both locomotor and sensory arousal (Figures 7E and 7F). This wireless pathway complements the reflexive escape response involving wired synapses and gap junctions between mechanosensory neurons, premotor interneurons and motorneurons leading to reversals (Chalfie et al., 1985) (Figure 7G). In contrast to the reversal response that is triggered instantaneously and persists for several seconds, the arousal response results in prolonged motor hyperactivity over a timescale of minutes. Moreover, while the arousal response depends on the release of FLP-20 peptides from the touch receptor neurons, reversals depend on coupling of sensory neurons to interneurons (Chalfie et al., 1985; Maricq et al., 1995) via chemical synapses, which are glutamatergic in the TRNs (Serrano-Saiz et al., 2013), and gap junctions. In addition, the glutamate transporter *eat-4* is required for normal responses to repeated taps (Rankin and Wicks, 2000), and the glutamate receptor *glr-1* is required for long-term memory for habituation to tap responses (Rose et al., 2003). Thus, co-transmission of glutamate and FLP-20 peptides by the mechanosensory TRNs may provide a means for the animal to alter locomotor activity in multiple ways and on multiple timescales. Another interesting example of co-transmission in *C. elegans* is the release of glutamate and NLP-1 neuropeptides from the sensory neuron AWC, where each neuromodulator has distinct effects on local search and responsiveness toward olfactory cues (Chalasani et al., 2010). Likewise, mouse neurons that release the arousal peptide orexin/hypocretin also release glutamate, which may serve to deliver complementary signals associated with firing of these neurons to downstream effectors of arousal (Schone and Burdakov, 2017). Thus, cotransmission represents a mechanism by which the animal can stabilize and consolidate important behavioral states, or respond to sensory cues of varying magnitudes and on different timescales (reviewed in Burnstock, 2004; Ma et al., 2017; Marder, 2012).

The nervous system of *C. elegans*, although relatively compact, shows surprising complexity within the connectome and in its repertoire of behavioral outputs (Walker et al., 2017). Its complement of neuropeptides and receptors is diverse and shows conservation with many other animals, including humans (Jekely, 2013; Mirabeau and Joly, 2013). Neuromodulators may tune signaling through the wired circuitry by promoting particular connections over others and could also increase the potential for plasticity (Bargmann, 2012; Marder, 2012). Future work could exploit this system as a model to understand the principles through which neuropeptide signaling networks interact with the synaptic connectome to control behavioral states.

STAR★METHODS

Detailed methods are provided in the online version of this paper and include the following:

- KEY RESOURCES TABLE
- CONTACT FOR REAGENT AND RESOURCE SHARING
- EXPERIMENTAL MODEL AND SUBJECT DETAILS
  - ○ Animals
  - ○ Microbe strains
- METHOD DETAILS
  - ○ Molecular Biology
  - ○ Behavioral assays
  - ○ Calcium imaging
  - ○ *In vitro* GPCR activation assays
  - ○ Confocal microscopy
- QUANTIFICATION AND STATISTICAL ANALYSIS
- DATA AND SOFTWARE AVAILABILITY

SUPPLEMENTAL INFORMATION

Supplemental Information includes seven figures and two tables and can be found with this article at https://doi.org/10.1016/j.neuron.2018.08.003.

A video abstract is available at http://dx.doi.org/10.1016/j.neuron.2018.08.003#mmc3.

ACKNOWLEDGMENTS

The authors gratefully acknowledge Denise Walker for critical reading of the manuscript, and members of the Schafer, de Bono, and Taylor labs (MRC LMB) and Young-Jai You (Nagoya University) for helpful discussions. We sincerely thank Inja Radman for some DNA constructs. Some strains were provided by the CGC, which is funded by the National Institutes of Health (NIH P40 OD010440). We are grateful to Dr. Mei Zhen for the RID::Chrimson line ZM9315. This work was funded by grants from the Medical Research Council (MC-A023-5PB91) and the Wellcome Trust (WT103784MA) to W.R.S., from the NIH (R21DC015652) to W.R.S. and H.L., from the NIH (R01NS096581 and R01GM088333) to H.L., from the Natural Sciences and Engineering Research Council of Canada (NSERC grant #122216-2013) to C.H.R., and from the NIH (R21DC016258) to J.B. Y.L.C. is funded by an EMBO Long-term Fellowship (ALTF 403-2016). A.J.Y. is funded by a NSERC Canada Graduate Scholarship-Master's (CGS-M). E.L.A. is funded by a NSERC CGS. I.B. is funded by an EMBO Long-term Fellowship (ALTF 387-2015) and is a fellow of the Research Foundation - Flanders (FWO).

AUTHOR CONTRIBUTIONS

Y.L.C. conceived and performed experiments, analyzed the data, and wrote the manuscript. Y.T. conceived and performed experiments and analyzed the data. Y.C., B.Z., E.L.A., A.J.Y., and I.R. performed experiments and analyzed the data. J.B., C.R., H.L., and I.B. secured funding and provided supervision. W.R.S. secured funding, conceived experiments, analyzed the data, provided supervision, and wrote the manuscript.

DECLARATION OF INTERESTS

The authors declare no competing interests.

Received: February 6, 2018
Revised: June 22, 2018
Accepted: August 2, 2018
Published: August 23, 2018

SUPPORTING CITATIONS

The following reference appears in the Figure S1 legend in the Supplemental Information: Yoshida et al. (2012).

REFERENCES

Aime, P., Duchamp-Viret, P., Chaput, M.A., Savigner, A., Mahfouz, M., and Julliard, A.K. (2007). Fasting increases and satiation decreases olfactory detection for a neutral odor in rats. Behav. Brain Res. *179*, 258–264.

Alkema, M.J., Hunter-Ensor, M., Ringstad, N., and Horvitz, H.R. (2005). Tyramine Functions independently of octopamine in the *Caenorhabditis elegans* nervous system. Neuron *46*, 247–260.

Altun-Gultekin, Z., Andachi, Y., Tsalik, E.L., Pilgrim, D., Kohara, Y., and Hobert, O. (2001). A regulatory cascade of three homeobox genes, *ceh-10*, *ttx-3* and *ceh-23*, controls cell fate specification of a defined interneuron class in *C. elegans*. Development *128*, 1951–1969.

Ardiel, E.L., Giles, A.C., Yu, A.J., Lindsay, T.H., Lockery, S.R., and Rankin, C.H. (2016). Dopamine receptor DOP-4 modulates habituation to repetitive photoactivation of a *C. elegans* polymodal nociceptor. Learn. Mem. *23*, 495–503.

Ardiel, E.L., Yu, A.J., Giles, A.C., and Rankin, C.H. (2017). Habituation as an adaptive shift in response strategy mediated by neuropeptides. NPJ Science of Learning *2*, 9.

Asahina, K., Watanabe, K., Duistermars, B.J., Hoopfer, E., Gonzalez, C.R., Eyjolfsdottir, E.A., Perona, P., and Anderson, D.J. (2014). Tachykinin-expressing neurons control male-specific aggressive arousal in *Drosophila*. Cell *156*, 221–235.

Barbas, D., DesGroseillers, L., Castellucci, V.F., Carew, T.J., and Marinesco, S. (2003). Multiple serotonergic mechanisms contributing to sensitization in *Aplysia*: evidence of diverse serotonin receptor subtypes. Learn. Mem. *10*, 373–386.

Bargmann, C.I. (2012). Beyond the connectome: how neuromodulators shape neural circuits. BioEssays *34*, 458–465.

Beets, I., Janssen, T., Meelkop, E., Temmerman, L., Suetens, N., Rademakers, S., Jansen, G., and Schoofs, L. (2012). Vasopressin/oxytocin-related signaling regulates gustatory associative learning in *C. elegans*. Science *338*, 543–545.

Bentley, B., Branicky, R., Barnes, C.L., Chew, Y.L., Yemini, E., Bullmore, E.T., Vértes, P.E., and Schafer, W.R. (2016). The Multilayer Connectome of *Caenorhabditis elegans*. PLoS Comput. Biol. *12*, e1005283.

Burnstock, G. (2004). Cotransmission. Curr. Opin. Pharmacol. *4*, 47–52.

Carew, T.J., Castellucci, V.F., and Kandel, E.R. (1971). An analysis of dishabituation and sensitization of the gill-withdrawal reflex in *Aplysia*. Int. J. Neurosci. *2*, 79–98.

Chalasani, S.H., Kato, S., Albrecht, D.R., Nakagawa, T., Abbott, L.F., and Bargmann, C.I. (2010). Neuropeptide feedback modifies odor-evoked dynamics in *Caenorhabditis elegans* olfactory neurons. Nat. Neurosci. *13*, 615–621.

Chalfie, M., Sulston, J.E., White, J.G., Southgate, E., Thomson, J.N., and Brenner, S. (1985). The neural circuit for touch sensitivity in *Caenorhabditis elegans*. J. Neurosci. *5*, 956–964.

Chalfie, M., Hart, A.C., Rankin, C.H., and Goodman, M.B. (2014). Assaying mechanosensation. WormBook.

Chao, M.Y., Komatsu, H., Fukuto, H.S., Dionne, H.M., and Hart, A.C. (2004). Feeding status and serotonin rapidly and reversibly modulate a *Caenorhabditis elegans* chemosensory circuit. Proc. Natl. Acad. Sci. USA *101*, 15512–15517.

Chen, X., and Chalfie, M. (2014). Modulation of *C. elegans* touch sensitivity is integrated at multiple levels. J. Neurosci. *34*, 6522–6536.

Chen, D., Taylor, K.P., Hall, Q., and Kaplan, J.M. (2016). The neuropeptides FLP-2 and PDF-1 act in concert to arouse *Caenorhabditis elegans* locomotion. Genetics *204*, 1151–1159.

Chiu, C.N., Rihel, J., Lee, D.A., Singh, C., Mosser, E.A., Chen, S., Sapin, V., Pham, U., Engle, J., Niles, B.J., et al. (2016). A Zebrafish genetic screen identifies neuromedin U as a regulator of sleep/wake states. Neuron *89*, 842–856.

Cho, Y., Porto, D., Hwang, H., Grundy, L., Schafer, W.R., and Lu, H. (2017). Automated and controlled mechanical stimulation and functional imaging *in vivo* in *C. elegans*. Lab on a Chip *17*, 2609–2618.

Cho, Y., Oakland, D.N., Lee, S.A., Schafer, W.R., and Lu, H. (2018). On-chip functional neuroimaging with mechanical stimulation in *Caenorhabditis elegans* larvae for studying development and neural circuits. Lab Chip *18*, 601–609.

Choi, S., Chatzigeorgiou, M., Taylor, K.P., Schafer, W.R., and Kaplan, J.M. (2013). Analysis of NPR-1 reveals a circuit mechanism for behavioral quiescence in *C. elegans*. Neuron *78*, 869–880.

Choi, S., Taylor, K.P., Chatzigeorgiou, M., Hu, Z., Schafer, W.R., and Kaplan, J.M. (2015). Sensory neurons arouse *C. elegans* locomotion via both glutamate and neuropeptide release. PLoS Genet. *11*, e1005359.

Curtright, A., Rosser, M., Goh, S., Keown, B., Wagner, E., Sharifi, J., Raible, D.W., and Dhaka, A. (2015). Modeling nociception in zebrafish: a way forward for unbiased analgesic discovery. PLoS ONE *10*, e0116766.

Donnelly, J.L., Clark, C.M., Leifer, A.M., Pirri, J.K., Haburcak, M., Francis, M.M., Samuel, A.D., and Alkema, M.J. (2013). Monoaminergic orchestration of motor programs in a complex *C. elegans* behavior. PLoS Biol. *11*, e1001529.

Elphick, M.R., and Mirabeau, O. (2014). The Evolution and Variety of RFamide-Type Neuropeptides: Insights from Deuterostomian Invertebrates. Front. Endocrinol. (Lausanne) *5*, 93.

Ezcurra, M., Tanizawa, Y., Swoboda, P., and Schafer, W.R. (2011). Food sensitizes *C. elegans* avoidance behaviours through acute dopamine signalling. EMBO J. *30*, 1110–1122.

Ezcurra, M., Walker, D.S., Beets, I., Swoboda, P., and Schafer, W.R. (2016). Neuropeptidergic Signaling and Active Feeding State Inhibit Nociception in *Caenorhabditis elegans*. J. Neurosci. *36*, 3157–3169.

Gray, J.M., Karow, D.S., Lu, H., Chang, A.J., Chang, J.S., Ellis, R.E., Marletta, M.A., and Bargmann, C.I. (2004). Oxygen sensation and social feeding mediated by a *C. elegans* guanylate cyclase homologue. Nature *430*, 317–322.

Gray, J.M., Hill, J.J., and Bargmann, C.I. (2005). A circuit for navigation in *Caenorhabditis elegans*. Proc. Natl. Acad. Sci. USA *102*, 3184–3191.

Harris, G.P., Hapiak, V.M., Wragg, R.T., Miller, S.B., Hughes, L.J., Hobson, R.J., Steven, R., Bamber, B., and Komuniecki, R.W. (2009). Three distinct amine receptors operating at different levels within the locomotory circuit are each essential for the serotonergic modulation of chemosensation in *Caenorhabditis elegans*. J. Neurosci. *29*, 1446–1456.

Hilliard, M.A., Apicella, A.J., Kerr, R., Suzuki, H., Bazzicalupo, P., and Schafer, W.R. (2005). *In vivo* imaging of *C. elegans* ASH neurons: cellular response and adaptation to chemical repellents. EMBO J. *24*, 63–72.

Hobert, O. (2013). The neuronal genome of *Caenorhabditis elegans*. In WormBook, E. Jorgensen, ed. (WormBook).

Horstick, E.J., Mueller, T., and Burgess, H.A. (2016). Motivated state control in larval zebrafish: behavioral paradigms and anatomical substrates. J. Neurogenet. *30*, 122–132.

Hubbard, C.S., Ornitz, E., Gaspar, J.X., Smith, S., Amin, J., Labus, J.S., Kilpatrick, L.A., Rhudy, J.L., Mayer, E.A., and Naliboff, B.D. (2011). Modulation of nociceptive and acoustic startle responses to an unpredictable threat in men and women. Pain *152*, 1632–1640.

Husson, S.J., Janssen, T., Baggerman, G., Bogert, B., Kahn-Kirby, A.H., Ashrafi, K., and Schoofs, L. (2007). Impaired processing of FLP and NLP peptides in carboxypeptidase E (EGL-21)-deficient *Caenorhabditis elegans* as analyzed by mass spectrometry. J. Neurochem. *102*, 246–260.

Iannacone, M.J., Beets, I., Lopes, L.E., Churgin, M.A., Fang-Yen, C., Nelson, M.D., Schoofs, L., and Raizen, D.M. (2017). The RFamide receptor DMSR-1 regulates stress-induced sleep in *C. elegans*. eLife *6*, e19837.

Im, S.H., Takle, K., Jo, J., Babcock, D.T., Ma, Z., Xiang, Y., and Galko, M.J. (2015). Tachykinin acts upstream of autocrine Hedgehog signaling during nociceptive sensitization in *Drosophila*. eLife *4*, e10735.

Jacob, T.C., and Kaplan, J.M. (2003). The EGL-21 carboxypeptidase E facilitates acetylcholine release at *Caenorhabditis elegans* neuromuscular junctions. J. Neurosci. *23*, 2122–2130.

Janssen, T., Husson, S.J., Meelkop, E., Temmerman, L., Lindemans, M., Verstraelen, K., Rademakers, S., Mertens, I., Nitabach, M., Jansen, G., et al. (2009). Discovery and characterization of a conserved pigment dispersing factor-like neuropeptide pathway in *Caenorhabditis elegans*. J. Neurochem. *111*, 228–241.

Jekely, G. (2013). Global view of the evolution and diversity of metazoan neuropeptide signaling. Proc. Natl. Acad. Sci. USA *110*, 8702–8707.

Kerr, R., Lev-Ram, V., Baird, G., Vincent, P., Tsien, R.Y., and Schafer, W.R. (2000). Optical imaging of calcium transients in neurons and pharyngeal muscle of *C. elegans*. Neuron *26*, 583–594.

Kim, K., and Li, C. (2004). Expression and regulation of an FMRFamide-related neuropeptide gene family in *Caenorhabditis elegans*. J. Comp. Neurol. *475*, 540–550.

Laurent, P., Soltesz, Z., Nelson, G.M., Chen, C., Arellano-Carbajal, F., Levy, E., and de Bono, M. (2015). Decoding a neural circuit controlling global animal state in *C. elegans*. eLife *4*, e04241.

Lebestky, T., Chang, J.S., Dankert, H., Zelnik, L., Kim, Y.C., Han, K.A., Wolf, F.W., Perona, P., and Anderson, D.J. (2009). Two different forms of arousal in *Drosophila* are oppositely regulated by the dopamine D1 receptor ortholog DopR via distinct neural circuits. Neuron *64*, 522–536.

Li, C., and Kim, K. (2014). Family of FLP Peptides in *Caenorhabditis elegans* and Related Nematodes. Front. Endocrinol. (Lausanne) *5*, 150.

Li, C., Timbers, T.A., Rose, J.K., Bozorgmehr, T., McEwan, A., and Rankin, C.H. (2013). The FMRFamide-related neuropeptide FLP-20 is required in the mechanosensory neurons during memory for massed training in *C. elegans*. Learn. Mem. *20*, 103–108.

Li, Z., Liu, J., Zheng, M., and Xu, X.Z. (2014). Encoding of both analog- and digital-like behavioral outputs by one *C. elegans* interneuron. Cell *159*, 751–765.

Lim, M.A., Chitturi, J., Laskova, V., Meng, J., Findeis, D., Wiekenberg, A., Mulcahy, B., Luo, L., Li, Y., Lu, Y., et al. (2016). Neuroendocrine modulation sustains the *C. elegans* forward motor state. eLife *5*, e19887.

Ma, S., Hangya, B., Leonard, C.S., Wisden, W., and Gundlach, A.L. (2017). Dual-transmitter systems regulating arousal, attention, learning and memory. Neurosci. Biobehav. Rev. *85*, 21–33.

Mahler, S.V., Moorman, D.E., Smith, R.J., James, M.H., and Aston-Jones, G. (2014). Motivational activation: a unifying hypothesis of orexin/hypocretin function. Nat. Neurosci. *17*, 1298–1303.

Marder, E. (2012). Neuromodulation of neuronal circuits: back to the future. Neuron *76*, 1–11.

Maricq, A.V., Peckol, E., Driscoll, M., and Bargmann, C.I. (1995). Mechanosensory signalling in *C. elegans* mediated by the GLR-1 glutamate receptor. Nature *378*, 78–81.

Mirabeau, O., and Joly, J.S. (2013). Molecular evolution of peptidergic signaling systems in bilaterians. Proc. Natl. Acad. Sci. USA *110*, E2028–E2037.

Pereira, L., Kratsios, P., Serrano-Saiz, E., Sheftel, H., Mayo, A.E., Hall, D.H., White, J.G., LeBoeuf, B., Garcia, L.R., Alon, U., et al. (2015). A cellular and regulatory map of the cholinergic nervous system of *C. elegans*. eLife *4*, e12432.

Pfaff, D., Ribeiro, A., Matthews, J., and Kow, L.M. (2008). Concepts and mechanisms of generalized central nervous system arousal. Ann. N Y Acad. Sci. *1129*, 11–25.

Pirri, J.K., McPherson, A.D., Donnelly, J.L., Francis, M.M., and Alkema, M.J. (2009). A tyramine-gated chloride channel coordinates distinct motor programs of a *Caenorhabditis elegans* escape response. Neuron *62*, 526–538.

Prober, D.A., Zimmerman, S., Myers, B.R., McDermott, B.M., Jr., Kim, S.H., Caron, S., Rihel, J., Solnica-Krezel, L., Julius, D., Hudspeth, A.J., et al. (2008). Zebrafish TRPA1 channels are required for chemosensation but not for thermosensation or mechanosensory hair cell function. J. Neurosci. *28*, 10102–10110.

Rabinowitch, I., Chatzigeorgiou, M., and Schafer, W.R. (2013). A gap junction circuit enhances processing of coincident mechanosensory inputs. Curr. Biol. *23*, 963–967.

Rabinowitch, I., Laurent, P., Zhao, B., Walker, D., Beets, I., Schoofs, L., Bai, J., Schafer, W.R., and Treinin, M. (2016a). Neuropeptide-Driven Cross-Modal Plasticity following Sensory Loss in *Caenorhabditis elegans*. PLoS Biol. *14*, e1002348.

Rabinowitch, I., Treinin, M., and Bai, J. (2016b). Artificial optogenetic TRN stimulation of *C. elegans*. Biol. Protoc. *6*, e1966.

Ramaekers, M.G., Verhoef, A., Gort, G., Luning, P.A., and Boesveldt, S. (2016). Metabolic and sensory influences on odor sensitivity in humans. Chem. Senses *41*, 163–168.

Ramot, D., Johnson, B.E., Berry, T.L., Jr., Carnell, L., and Goodman, M.B. (2008). The Parallel Worm Tracker: a platform for measuring average speed and drug-induced paralysis in nematodes. PLoS ONE *3*, e2208.

Rankin, C.H., and Wicks, S.R. (2000). Mutations of the *Caenorhabditis elegans* brain-specific inorganic phosphate transporter *eat-4* affect habituation of the tap-withdrawal response without affecting the response itself. J. Neurosci. *20*, 4337–4344.

Rankin, C.H., Beck, C.D., and Chiba, C.M. (1990). *Caenorhabditis elegans*: a new model system for the study of learning and memory. Behav. Brain Res. *37*, 89–92.

Rogers, C., Reale, V., Kim, K., Chatwin, H., Li, C., Evans, P., and de Bono, M. (2003). Inhibition of *Caenorhabditis elegans* social feeding by FMRFamide-related peptide activation of NPR-1. Nat. Neurosci. *6*, 1178–1185.

Rose, J.K., and Rankin, C.H. (2001). Analyses of habituation in *Caenorhabditis elegans*. Learn. Mem. *8*, 63–69.

Rose, J.K., Kaun, K.R., Chen, S.H., and Rankin, C.H. (2003). GLR-1, a non-NMDA glutamate receptor homolog, is critical for long-term memory in *Caenorhabditis elegans*. J. Neurosci. *23*, 9595–9599.

Schone, C., and Burdakov, D. (2017). Orexin/hypocretin and organizing principles for a diversity of wake-promoting neurons in the brain. Curr. Top. Behav. Neurosci. *33*, 51–74.

Serrano-Saiz, E., Poole, R.J., Felton, T., Zhang, F., De La Cruz, E.D., and Hobert, O. (2013). Modular control of glutamatergic neuronal identity in *C. elegans* by distinct homeodomain proteins. Cell *155*, 659–673.

Speese, S., Petrie, M., Schuske, K., Ailion, M., Ann, K., Iwasaki, K., Jorgensen, E.M., and Martin, T.F.J. (2007). UNC-31 (CAPS) is required for dense-core vesicle but not synaptic vesicle exocytosis in *Caenorhabditis elegans*. J. Neurosci. *27*, 6150–6162.

Stirman, J.N., Crane, M.M., Husson, S.J., Wabnig, S., Schultheis, C., Gottschalk, A., and Lu, H. (2011). Real-time multimodal optical control of neurons and muscles in freely behaving *Caenorhabditis elegans*. Nat. Methods *8*, 153–158.

Suzuki, H., Kerr, R., Bianchi, L., Frokjaer-Jensen, C., Slone, D., Xue, J., Gerstbrein, B., Driscoll, M., and Schafer, W.R. (2003). *In vivo* imaging of *C. elegans* mechanosensory neurons demonstrates a specific role for the MEC-4 channel in the process of gentle touch sensation. Neuron *39*, 1005–1017.

Taghert, P.H., and Nitabach, M.N. (2012). Peptide neuromodulation in invertebrate model systems. Neuron *76*, 82–97.

Turek, M., Besseling, J., Spies, J.P., Konig, S., and Bringmann, H. (2016). Sleep-active neuron specification and sleep induction require FLP-11 neuropeptides to systemically induce sleep. eLife *5*, e12499.

Van Bael, S., Watteyne, J., Boonen, K., De Haes, W., Menschaert, G., Ringstad, N., Horvitz, H.R., Schoofs, L., Husson, S.J., and Temmerman, L. (2018). Mass spectrometric evidence for neuropeptide-amidating enzymes in *Caenorhabditis elegans*. J. Biol. Chem. *293*, 6052–6063.

Van Buskirk, C., and Sternberg, P.W. (2010). Paired and LIM class homeodomain proteins coordinate differentiation of the *C. elegans* ALA neuron. Development *137*, 2065–2074.

van Swinderen, B., and Andretic, R. (2003). Arousal in *Drosophila*. Behav. Processes *64*, 133–144.

Walker, D.S., Chew, Y.L., and Schafer, W.R. (2017). Genetics of behavior in *C. elegans*. In The Oxford Handbook of Invertebrate Neurobiology, J.H. Byrne, ed. (Oxford University Press).

Wester, J.C., and McBain, C.J. (2014). Behavioral state-dependent modulation of distinct interneuron subtypes and consequences for circuit function. Curr. Opin. Neurobiol. *29*, 118–125.

White, J.G., Southgate, E., Thomson, J.N., and Brenner, S. (1986). The structure of the nervous system of the nematode *Caenorhabditis elegans*. Philos. Trans. R. Soc. Lond. B Biol. Sci. *314*, 1–340.

Wicks, S.R., and Rankin, C.H. (1995). Integration of mechanosensory stimuli in *Caenorhabditis elegans*. J. Neurosci. *15*, 2434–2444.

Woods, I.G., Schoppik, D., Shi, V.J., Zimmerman, S., Coleman, H.A., Greenwood, J., Soucy, E.R., and Schier, A.F. (2014). Neuropeptidergic signaling partitions arousal behaviors in zebrafish. J. Neurosci. *34*, 3142–3160.

Yokogawa, T., Hannan, M.C., and Burgess, H.A. (2012). The dorsal raphe modulates sensory responsiveness during arousal in zebrafish. J. Neurosci. *32*, 15205–15215.

Yoshida, K., Hirotsu, T., Tagawa, T., Oda, S., Wakabayashi, T., Iino, Y., and Ishihara, T. (2012). Odour concentration-dependent olfactory preference change in *C. elegans*. Nat. Commun. *3*, 739.

STAR★METHODS

KEY RESOURCES TABLE

REAGENT or RESOURCE	SOURCE	IDENTIFIER
Bacterial and Virus Strains		
E. coli: Strain OP50	*Caenorhabditis* Genetics Center (CGC)	WormBase: OP50
Chemicals, Peptides, and Recombinant Proteins		
AMMRFa, AVFRMa, SVFRLa	GL Biochem (Shangai)	N/A
Experimental Models: Cell Lines		
CHO-K1 cell line	PerkinElmer	ES-000-A2
Experimental Models: Organisms/Strains		
C. elegans: Strain AQ4045 *ljEx1107[Psra-6::GCaMP3::SL2-tagRFP(50);unc-122::RFP(50)]*	This study	N/A
C. elegans: Strain AQ4076 *flp-20(ok2964);ljEx1107*	This study	N/A
C. elegans: Strain AQ4077 *frpr-3(ok3302);ljEx1107*	This study	N/A
C. elegans: Strain AQ4168 *frpr-3(ok3302);ljEx1149[Pdes-2::frpr-3cDNA::gpd-2 3′ UTR(pYLC219)(25);unc-122::gfp(50)];ljEx1107*	This study	N/A
C. elegans: Strain AQ4169 *frpr-3(ok3302);ljEx1150[Pflp-2::frpr-3cDNA::gpd-2 3′ UTR(pYLC220)(25);unc-122::gfp(50)];ljEx1107*	This study	N/A
C. elegans: Strain AQ4173 *flp-20(ok2964);ljEx1094[Pmec-4::flp-20 gDNA + 3′ UTR::SL2-mKate2 (50);unc-122::gfp(50)];ljEx1107*	This study	N/A
C. elegans: Strain AQ2052 *lite-1(ce314);ljIs105[sra-6::ChR2::yfp, unc-122::gfp]*	This study	N/A
C. elegans: Strain AQ2235 *lite-1(ce314); ljIs114[Pgpa-13::FLPase, Psra-6::FTF::ChR2::YFP] X*	This study	N/A
C. elegans: Strain AQ2755 *lite-1(ce314); ljIs124[Pgpa-13::FLPase, Psra-6::FTF::ChR2::YFP]not X*	This study	N/A
C. elegans: Strain VG266 *frpr-3(gk240031) (backcrossed 3x); lite-1(ce314); ljIs114*	This study	N/A
C. elegans: Strain AQ2786 *flp-20 (ok2964) lite-1(ce314) X; ljIs124*	This study	N/A
C. elegans: Strain AQ3941 *lite-1(ce314) flp-20(ok2964)X; ljIs124; Ex[Pmec-4::flp-20 cDNA)(20);unc-122::gfp(20)]* line-1	This study	N/A
C. elegans: Strain AQ3940 *lite-1(ce314) flp-20(ok2964)X; ljIs124; Ex[Pflp-20::flp-20 cDNA (20), ccGFP (20)]* line-1	This study	N/A
C. elegans: Strain AQ4246 *lite-1(ce314) flp-20(ok2964)X; ljIs124; Ex[Pflp-20::flp-20 cDNA (20), ccGFP (20)]* line-2	This study	N/A
C. elegans: Strain AQ4247 *lite-1(ce314) flp-20(ok2964)X; ljIs124; Ex[Pflp-20::flp-20 cDNA (20), ccGFP (20)]* line-3	This study	N/A
C. elegans: Strain AQ4248 *lite-1(ce314) flp-20(ok2964)X; ljIs124; Ex[Pmec-4::flp-20 cDNA)(20);unc-122::gfp(20)]* line-2	This study	N/A
C. elegans: Strain AQ4249 *lite-1(ce314) flp-20(ok2964)X; ljIs124; Ex[Pmec-4::flp-20 cDNA)(20);unc-122::gfp(20)]* line-3	This study	N/A
C. elegans: Strain AQ4250 *lite-1(ce314) flp-20(ok2964)X; ljIs124; Ex[Pgcy-5::flp-20(10); Pgcy-7::flp-20(10);unc-122::gfp(20)]* line-1	This study	N/A
C. elegans: Strain AQ4251 *lite-1(ce314) flp-20(ok2964)X; ljIs124; Ex[Pgcy-5::flp-20(10); Pgcy-7::flp-20(10);unc-122::gfp(20)]* line-2	This study	N/A
C. elegans: Strain AQ4252 *lite-1(ce314) flp-20(ok2964)X; ljIs124; Ex[Pgcy-5::flp-20(10); Pgcy-7::flp-20(10);unc-122::gfp(20)]* line-3	This study	N/A
C. elegans: Strain AQ4260 *lite-1(ce314);ljIs124;ljEx1187[Psra-6::frpr-3 antisense RNAi(50);Psra-6::frpr-3 sense RNAi(50);ccGFP(30)]*	This study	N/A

(*Continued on next page*)

Continued

REAGENT or RESOURCE	SOURCE	IDENTIFIER
C. elegans: Strain AQ4262 *lite-1(ce314);ljIs124;ljEx1189[Psra-9::frpr-3 antisense RNAi(55);Psra-9::frpr-3 sense RNAi(55);ccGFP(30)]*	This study	N/A
C. elegans: Strain AQ4264 *lite-1(ce314);ljIs124;ljEx1191[Pflp-2::frpr-3 antisense RNAi(50);Pdes-2::frpr-3 sense RNAi(50);ccGFP(30)]*	This study	N/A
C. elegans: Strain AQ4320 *lite-1(ce314);ljIs124;ljEx1214[Pdes-2::egl-21 antisense RNAi(50);Pflp-2::egl-21 sense RNAi(50);ccGFP(40)]*	This study	N/A
C. elegans: Strain AQ4346 *lite-1(ce314);ljIs124;ljEx1228[Pflp-2::frpr-3 antisense RNAi(50);Pceh-10(3.6)::frpr-3 sense RNAi(50);ccGFP(50)]*	This study	N/A
C. elegans: Strain AQ4355 *lite-1(ce314);ljIs124;ljEx1229[Pdes-2::frpr-3 antisense RNAi(50);Pceh-10(3.6)::frpr-3 sense RNAi(50);ccGFP(50)]*	This study	N/A
C. elegans: Strain AQ4356 *lite-1(ce314);ljIs124;ljEx1230[Pins-17(2kb)::frpr-3 antisense RNAi(50);Pceh-10(3.6)::frpr-3 sense RNAi(50);ccGFP(50)]*	This study	N/A
C. elegans: Strain AQ4360 *lite-1(ce314);ljIs124;ljEx1234[Pflp-2::unc-31 antisense RNAi(50);Pdes-2::unc-31 sense RNAi(50);ccGFP(50))]*	This study	N/A
C. elegans: Strain AQ4364 *lite-1(ce314);ljIs124;ljEx1238[Pttx-3::frpr-3 antisense RNAi(50);Pttx-3::frpr-3 sense RNAi(50);unc-122::gfp(50)]*	This study	N/A
C. elegans: Strain AQ4365 *lite-1(ce314);ljIs124;ljEx1239[Pflp-1::frpr-3 antisense RNAi(50);Pflp-1::frpr-3 sense RNAi(50);unc-122::gfp(50)]*	This study	N/A
C. elegans: Strain AQ4390 *lite-1(ce314);ljIs124;hpIs626(Pceh10::Chrimson::GFP::ZF; Pttx-3::ZIF-1::SL2::RFP; Pgpa-14::ZIF-1::SL2::RFP);hpEx3808(Parr-1-ZIF-1::SL2::RFP;Pmyo-3::rfp)*	This study	N/A
C. elegans: Strain AQ4023 ljEx1093[Pflp-20::flp-20 gDNA + 3′ UTR::SL2-mKate2(50);unc-122::gfp(50)]	This study	N/A
C. elegans: Strain AQ4006 *ljEx1090[Pfrpr-3::frpr-3 gDNA::SL2-mKate(25); unc-122::gfp(50)]*	This study	N/A
C. elegans: Strain TU253 mec-4(u253) X	CGC	Wormbase: TU253
C. elegans: Strain AQ4019 *frpr-3(ok3302);ljEx1090*	This study	N/A
C. elegans: Strain AQ4035 *flp-20(ok2964);ljEx1094*	This study	N/A
C. elegans: Strain AQ4037 *flp-20(ok2964);ljEx1093*	This study	N/A
C. elegans: Strain AQ4054 *frpr-3(ok3302);flp-20(ok2964)*	This study	N/A
C. elegans: Strain AQ4072 *flp-20(ok2964);frpr-3(ok3302);ljEx1093*	This study	N/A
C. elegans: Strain AQ4078 *frpr-3(ok3302);ljEx1108[Pflp-1::frpr-3 gDNA + UTR(10);unc-122::GFP(50)]*	This study	N/A
C. elegans: Strain AQ4104 *frpr-3(ok3302);ljEx1135[Pflp-2::frpr-3::SL2-mKate2 (50);unc-122::gfp(50)]*	This study	N/A
C. elegans: Strain AQ4105 *frpr-3(ok3302);ljEx1136[Pdes-2::frpr-3::SL2-mKate2 (50);unc-122::gfp(50)]*	This study	N/A
C. elegans: Strain AQ4179 *frpr-3(ok3302);ljEx1167[Pceh-10(3.6)::frpr-3::SL2-mKate2(pYLC232)(50);unc-122::gfp(50)]*	This study	N/A
C. elegans: Strain AQ4087 *frpr-3(ok3302);ljEx1123[Psra-9::frpr-3 gDNA + 3′ UTR::SL2-mKate2(50);ccGFP(50)]*	This study	N/A
C. elegans: Strain AQ4103 *frpr-3(ok3302);ljEx1134[Pttx-3::frpr-3::SL2-mKate2 (50)(pYLC190);ccGFP(50)]*	This study	N/A
C. elegans: Strain AQ3832 *frpr-3(ok3302) V* backcrossed 6x	This study	N/A
C. elegans: Strain AQ4000 *flp-20(ok2964) X* backcrossed 6x	This study	N/A
C. elegans: Strain AQ4396 *ljEx1246[Pdes-2::mKate2::gpd-2 3′ UTR(25);ccGFP(50)];ljEx1247[Pflp-2:gfp::gpd-2 3′ UTR(25);ccRFP(50)]*	This study	N/A
C. elegans: Strain AQ4397 *ljEx1246[Pdes-2::mKate2::gpd-2 3′ UTR(25);ccGFP(50)];ljEx1165[Pceh-10(3.6)::YC3.60::gpd-2 3′ UTR(25);ccRFP(50)]*	This study	N/A
C. elegans: Strain AQ4398 *unc-119(ed3) III; wwEx73(ins-17p::gfp + unc-119(+));ljEx1167[Pceh-10(3.6)::frpr-3::SL2-mKate2(50);ccGFP(50)]*	This study	N/A

(*Continued on next page*)

Continued

REAGENT or RESOURCE	SOURCE	IDENTIFIER
C. elegans: Strain AQ4399 *ljEx1167[Pceh-10(3.6)::frpr-3::SL2-mKate2(50); ccGFP(50)];ljEx1247[Pflp-2:gfp::gpd-2 3′ UTR(50);ccRFP(50)]*	This study	N/A
C. elegans: Strain PT505 *flp-20(pk1596) X*	CGC	Wormbase: PT505
C. elegans: Strain VC2565 *frpr-3(ok3302) V*	CGC	Wormbase: VC2565
C. elegans: Strain BJH387 *flp-20(pk1596) X; frpr-3(ok3302) V*	This study	N/A
C. elegans: Strain AQ4144 *ljEx1165[Pceh-10(3.6)::YC3.60::gpd-2 3′ UTR (pYLC233)(50);unc-122::rfp(50)]*	This study	N/A
C. elegans: Strain AQ4187 *frpr-3(ok3302);ljEx1165*	This study	N/A
C. elegans: Strain AQ4188 *flp-20(ok2964);ljEx1165*	This study	N/A
C. elegans: Strain AQ4210 *flp-20(ok2964);ljEx1094;ljEx1165*	This study	N/A
C. elegans: Strain AQ4211 *frpr-3(ok3302);ljEx1167;ljEx1165*	This study	N/A
C. elegans: Strain AQ2766 *dop-2 (vs105)V;lite-1(ce314);ljIs114 X (ASH::ChR2)*	This study	N/A
C. elegans: Strain AQ2767 *npr-3(tm1583); lite-1(ce314);ljIs114 X (ASH::ChR2)*	This study	N/A
C. elegans: Strain AQ2768 *npr-5 (ok1583)V; lite-1(ce314);ljIs114 X (ASH::ChR2)*	This study	N/A
C. elegans: Strain AQ2769 *npr-13 (tm1504)V; lite-1(ce314);ljIs114 X (ASH::ChR2)*	This study	N/A
C. elegans: Strain AQ2770 *flp-6 (ok3056) V; lite-1(ce314);ljIs114 X (ASH::ChR2)*	This study	N/A
C. elegans: Strain AQ2772 *flp-21 (ok889)V; lite-1(ce314);ljIs114 X (ASH::ChR2)*	This study	N/A
C. elegans: Strain AQ2773 *mod-1 (ok103)V; lite-1(ce314);ljIs114 X (ASH::ChR2)*	This study	N/A
C. elegans: Strain AQ2779 *flp-4 II; lite-1(ce314);ljIs114 X (ASH::ChR2)*	This study	N/A
C. elegans: Strain AQ2781 *tyra-3 (ok325)X; lite-1(ce314);ljIs124 non-X (ASH::ChR2)*	This study	N/A
C. elegans: Strain AQ2782 *npr-1 (ad609)X; lite-1(ce314);ljIs124 non-X (ASH::ChR2)*	This study	N/A
C. elegans: Strain AQ2783 *flp-7 (ok2625)X; lite-1(ce314);ljIs124 non-X (ASH::ChR2)*	This study	N/A
C. elegans: Strain AQ2785 *flp-18 (dp99)X; lite-1(ce314);ljIs124 non-X (ASH::ChR2)*	This study	N/A
C. elegans: Strain AQ2786 *flp-20 (ok2964)X; lite-1(ce314);ljIs124 non-X (ASH::ChR2)*	This study	N/A
C. elegans: Strain AQ4405 *ser-7 (tm1325)X; ljIs105(Psra-6::ChR2)*	This study	N/A
C. elegans: Strain AQ4406 *tag-24 (ok371)X; ljIs105(Psra-6::ChR2)*	This study	N/A
C. elegans: Strain AQ4407 *ser-2 (pk1357)X; ljIs105(Psra-6::ChR2)*	This study	N/A
C. elegans: Strain AQ4408 *tyra-2 (tm1846)X; ljIs105(Psra-6::ChR2)*	This study	N/A
C. elegans: Strain AQ4409 *tyra-2 (tm1846)X; ljIs105(Psra-6::ChR2)*	This study	N/A
C. elegans: Strain AQ4410 *lgc-55 (tm2913)V; ljIs105(Psra-6::ChR2)*	This study	N/A
C. elegans: Strain AQ4411 *dop-6/C24A8.1 (ok2090)X; ljIs105(Psra-6::ChR2)*	This study	N/A
C. elegans: Strain AQ4412 *npr-1 (ad609)X; ljIs105(Psra-6::ChR2)*	This study	N/A
C. elegans: Strain AQ4413 *npr-2 (ok419)IV; ljIs105(Psra-6::ChR2)*	This study	N/A
C. elegans: Strain AQ4414 *npr-7 (ok527)X; ljIs105(Psra-6::ChR2)*	This study	N/A
C. elegans: Strain AQ4415 *npr-8 (tm1553)X; ljIs105(Psra-6::ChR2)*	This study	N/A
C. elegans: Strain AQ4416 *npr-10 (tm1568)X; ljIs105(Psra-6::ChR2)*	This study	N/A
C. elegans: Strain AQ4417 *npr-11 (ok594)X; ljIs105(Psra-6::ChR2)*	This study	N/A
C. elegans: Strain AQ4418 *npr-12 (tm1498)IV; ljIs105(Psra-6::ChR2)*	This study	N/A

(*Continued on next page*)

Continued		
REAGENT or RESOURCE	SOURCE	IDENTIFIER
C. elegans: Strain AQ4419 *flp-11 (tm2706)X; ljIs105(Psra-6::ChR2)*	This study	N/A
C. elegans: Strain AQ4420 *flp-17 (ok3587)IV; ljIs105(Psra-6::ChR2)*	This study	N/A
C. elegans: Strain AQ4421 *flp-18 (dp99)X; ljIs105(Psra-6::ChR2)*	This study	N/A
C. elegans: Strain AQ4422 *flp-19 (ok2460)X; ljIs105(Psra-6::ChR2)*	This study	N/A

CONTACT FOR REAGENT AND RESOURCE SHARING

Further information and requests for resources and reagents should be directed to and will be fulfilled by the Lead Contact, William Schafer (wschafer@mrc-lmb.cam.ac.uk).

EXPERIMENTAL MODEL AND SUBJECT DETAILS

Animals

Strains were maintained on NGM (nematode growth medium) plates seeded with E. coli strain OP50 according to standard experimental procedures. Young adult hermaphrodite animals were used for all experiments. Where indicated, mutant alleles were backcrossed 3-6 x to our laboratory stock of N2 (wild-type). For a list of strains and transgene details, see Table S1 and the Key Resources Table.

Microbe strains

The *Escherichia coli* OP50 strain was used as a food source for *C. elegans*.

METHOD DETAILS

Molecular Biology

Transgenes were cloned using the Multisite Gateway Three-Fragment cloning system (12537-023, Invitrogen) into pDESTR4R3 II. For transgenic reporter lines reported here, the length of the promoter (number of bases before ATG) for each gene is as follows: *E01H11.3/flp-20* 3081 bases; *C26F1.6/frpr-3* 2573 bases. For the list of reporter transgenic lines used to confirm the expression patterns of the above genes, see **Table S2**. For *frpr-3* RNAi, 700 bases of cDNA starting from the start codon was cloned in both the antisense or sense orientation. For *unc-31* RNAi, 703 bases of cDNA starting from the sequence GTTGTCGTGATGGAAGTGC was cloned in both the antisense or sense orientation. For *egl-21* RNAi, 652 bases of cDNA starting from the sequence GTGCTTTTGGTTGC was cloned in both the antisense or sense orientation. For cell-specific transgenic lines, the promoter lengths (bp upstream of ATG) used were: *Pmec-4* 1021 bp (TRNs), *Pgcy-5* 2012 bp (ASER), *Pgcy-7* 1198 bp (ASEL), *Pceh-10* 3583 bp (RID + others), *Pflp-2* 2000 bp (RID + others), *Pdes-2* 2581 bp (RID + others), P*ins-17* 2000 bp (RID + others), *Pflp-1* 1571 bp (AVK), *Psra-6* 2963 bp (ASH), *Psra-9* 3047 bp (ASK), and P*ttx-3* 243 bp sequence of the second intron (AIY). For cell-specific RNAi experiments, we knocked down expression in the following cells using the promoters in brackets: AIY (P*ttx-3*), ASH (P*sra-6*), ASK (P*sra-9*), AVK (P*flp-1*) and RID (four combinations: P*flp-2*/P*des-2*, P*flp-2*/P*ceh-10*, P*des-2*/P*ceh-10*, and P*ceh-10*/P*ins-17*).

Behavioral assays

For all experiments, young adult hermaphrodite animals were used, therefore sample stratification was not required within each genotype/condition. For most experiments, measurements were scored by automated algorithm so blind scoring was not undertaken: see each subsection for details. For details of statistical tests, see the relevant Figure legend for each experiment and also the subsection "Quantification and Statistical Analysis." All recordings that passed the automated analysis pipeline were included in the final dataset.

Locomotion (tap) arousal experiments: 1-2 day old well-fed adult animals were used for all experiments. 16 hours before the experiment, late L4 animals were picked onto NGM plates seeded with 90 μL of OP50 bacteria left to grow at room temperature for 14-16 hours. 3-4 plates of > 50-100 animals per plate were tested per trial, with each genotype tested in at least 3 trials. Multi worm tracking was performed using a Dinolite camera positioned above the plates, which recorded videos at 5 frames per second. An additional white LED backlight was used to improve contrast. Video recording was started 2 min after the plate was placed on the stage. Animals were recorded for 20 s before taps were applied and then for a further 5 min. Taps were applied manually to the bottom of the assay plate using the blunt end of a pencil (5 taps applied in < 3 s). Tracks were analyzed using MATLAB (MathWorks) (Ramot et al., 2008). Speed plots show the absolute speed (including forward and backward locomotion). Arousal speed was determined by quantifying the average speed 10 s after tap, as the initial (acute) response is thought to largely include reversals (Rankin et al., 1990). The arousal integral (area under the curve) measurements obtained in the 5-20 s following tap stimulus (for 5x tap) or 2-10 s (for 1x tap) were performed using GraphPad Prism, with the baseline values used for each genotype being the speed measurements recorded

before stimulus was applied. Note that arousal integrals for 1x taps are smaller than for 5x taps (Figure 1A); in addition, the presence of a *lite-1* mutation in the background affects locomotion and is likely to impact the absolute arousal measurements shown in Figure 7F and Figure S7C. As speed measurements were conducted using an automated algorithm, genotypes were not blinded prior to analysis.

For other stimulations: odorants (1% in ethanol) benzaldehyde, (undiluted) 2-nonanone and (undiluted) diacetyl were dropped in front of the nose of the worm by mouth pipetting. Heat was applied by placing a heated platinum wire pick in front of the nose of the worm. "Pick" refers to animals being picked up using a platinum wire pick and immediately placed back onto the bacterial lawn. Taps were applied manually to the bottom of the assay plate using the blunt end of a pencil. Harsh touch refers to prodding of the worm body using a platinum wire pick. At least 5-10 animals were tested per condition, at least twice. For glycerol drop tests, 1 M glycerol was mouth pipetted close to the head as previously described (Ezcurra et al., 2011), with 5 minutes of acclimatization on the plate prior to application of the first stimulus. For this assay, reversal distance was counted by eye as the number of body bends observed during the reversal response.

Channelrhodopsin experiments: 1-2 day old well-fed adult animals were used for all experiments. For LED stimulation: for experiments with *flp-20* mutant animals, a custom setup was used as described in (Ezcurra et al., 2011); for experiments on *frpr-3* mutant animals and RNAi transgenic lines, a custom setup was used as in (Ardiel et al., 2016; Ardiel et al., 2017) using 480 nm Luxeon star LEDs with 450 uw/mm^2 power. In contrast to locomotion arousal experiments described above, these experiments used an automated tapper, which produces equivalent speed responses (Figure S1C). *C. elegans* does not produce the co-factor all-trans retinal (ATR) required for ChR2 function. ATR (R2500 Sigma-Aldrich) was provided to animals by feeding as described in (Rabinowitch et al., 2016b). > 20 animals were placed onto the seeded plate and at least 4 plates were assayed per condition and genotype on multiple days for all cross-modal sensitization behavioral experiments. Video recording was started 5 min after the plate was placed on the stage. For animals exposed to a pre-arousing tap, 1 tap was used, followed by a 20 s interval and then a 2 s blue light stimulation. For Figure 1B, the integrated transgene *Psra-6::ChR2* was used (*Psra-6::ChR2::YFP*). For all other experiments, the integrated transgene *ASH::ChR2* was used (*Pgpa-13::FLPase*, *Psra-6::FTF::ChR2::YFP*) (Ezcurra et al., 2011). "Wild-type" controls for these experiments were animals containing the *lite-1(ce314)* allele and the *ASH::ChR2* transgene. See Table S1 for full genotype information. As reversal distance measurements were conducted using an automated algorithm, genotypes were not blinded prior to analysis.

To test if 2-nonanone exposure could sensitize ASH-dependent reversals, 200 μL undiluted nonanone was spread with a pipette around the rim inside the lid of Petri dish. After 300 s acclimatization, the lid was changed from a control (no odorant) lid to the 2-nonanone-lid and blue light provided 40 s later. To test if tap in the presence of benzaldehyde could affect sensitization responses, 1% benzaldehyde in ethanol was spread evenly across the lid of the Petri dish and dried for 5 minutes in a fume cupboard. Control lids were prepared in the same way with ethanol alone. The protocol is as follows: 300 s acclimatization > change lid from control to benzaldehyde-lid > 20 s wait > tap > 20 s wait > blue light (2 s). Two control groups were tested, in both groups the lid was changed to another control lid: one group was provided with an arousing tap stimulus and the other was not pre-aroused. For the *RID::Chrimson/ASH::ChR2* dual stimulation experiment, the protocol is 300 s acclimatization > red light for 180 s (or no stimulus) > tap (or no stimulus) > 20 s wait > blue light. Three groups were tested: prior to ASH activation with blue light, one group was stimulated with red light, one received a tap, and the naive group received neither stimuli. To provide dual-color light stimulation, a ring-shaped apparatus was constructed to contain 12 LEDs (6 red [630 nm, 600 uw/mm^2 Multicomp star] and 6 blue [480 nm, 350 uw/mm^2, Luxeon star]). The diameter of this ring is larger than the diameter of the 6-LED ring used for all other experiments, meaning that the blue light intensity is weaker than that in other assays (resulting in a reduced reversal response to optogenetic activation of ASH). LED stimulation was controlled by the Multi-Worm tracker software. For all experiments, reversal length was analyzed using MATLAB (Ardiel et al., 2017; Ezcurra et al., 2011; Ramot et al., 2008).

Touch assays: Gentle body touch assays were performed on day 1 adults by stroking with an eyelash hair, as described (Chalfie et al., 2014). Assays were conducted blind to the genotype of the strains.

Off-food reversal rate: The reversing assay was performed as previously described (Rabinowitch et al., 2016a). A single adult worm was removed from food, allowed to crawl for a few seconds until no traces of food were visible in its track, and then transferred to an empty 6 cm NGM plate. After 1 min, reversing events consisting of at least one body bend were counted over a 3 min period.

Calcium imaging

In microfluidics chip: Calcium imaging on 1-2 day old adult animals was performed in custom-designed microfluidic devices as described (using mechanical stimulus module from Cho et al., 2018; Cho et al., 2017). These experiments were performed on a Leica DMIRB inverted microscope using a 40x air objective (N.A. 0.75). Video sequences were captured using a Hamamatsu EM-CCD camera with 100 ms exposure time. Simultaneous dual color imaging was performed using a DV2 beamsplitter (Photometrics) containing a GFP(520 nm)/RFP(605 nm) filter set. Excitation light for fluorescent imaging was delivered through a projector system (Stirman et al., 2011). Stimuli were delivered as follows: for experiments where only a single chemical stimulus was provided, a 10 s pulse of 0.5 M glycerol in S-basal was delivered at t = 10 s after recordings were started, whereas for experiments where both mechanical and chemical stimuli were provided, a single (25 psi) mechanical stimulus to the anterior body was delivered at t = 30 s after the start of the recording, followed 20 s later by a 10 s pulse of 0.5 M glycerol (from t = 50-60 s). Note that based on genetic criteria (dependence on MEC-4/TRNs) the 25 psi stimulation is thought to be most equivalent to the tap stimulations applied in behavioral experiments (Cho et al., 2018; Wicks and Rankin, 1995). Videos were recorded for 40-90 s following stimulus delivery. For analysis of

calcium transients, fluorescence intensities for each frame were extracted using a custom MATLAB script (Cho et al., 2018; Cho et al., 2017). The GCaMP3/tagRFP ratio (R) between intensity values was computed $(R = I_{Green\_ROI}/I_{Red\_ROI})$ to minimize movement artifacts. GCaMP3 and RFP intensities were measured as the mean pixel intensity of the 100 brightest pixels in a circular region of interest (ROI) with a 10 pixel radius. Calcium traces were computed as the change in R from the baseline value $(\Delta R/R_o = (R - R_o)/R_o)$. Baseline values were computed as the mean R prior to stimulus delivery. Imaging was carried out in S-basal buffer (100mM NaCl, 0.05M phosphate buffer pH6.0, 5 μg/mL cholesterol). For two chemical stimulations (nonanone and glycerol), a Y-shaped connector (Norodson Medical, Y210-6005) was used to connect two chemical reservoirs and two off-chip solenoid valves for each chemical streamline, allowing for a rapid switch from the first stimulus to buffer and then to the second chemical stimulus. All quantification is provided as the ratio of GCaMP3 to tagRFP fluorescence intensity. As measurements were conducted using an automated algorithm, genotypes were not blinded prior to analysis.

Glued procedure: Calcium imaging of body touch stimulation of glued animals was performed essentially as described (Kerr et al., 2000; Suzuki et al., 2003), using a 1 s stimulus in the posterior end of the worm equidistant between the tail and the vulva. Stimulus was provided at t = 10 s after the start of the recording and videos were recorded for a total of 55 s. Images were recorded at 10 Hz using an iXon EM camera (Andor Technology), captured using IQ1.9 software (Andor Technology) and analyzed using a custom MATLAB (MathWorks) program (Rabinowitch et al., 2013). Fluorescence intensity, F, was computed as the difference between the sum of pixel intensities and the faintest 10% pixels (background) within the ROI. As the calcium sensor Cameleon (YC3.60) was used in this experiment, fluorescence ratio R = F_{YFP}/F_{CFP} (after correcting for bleed through) was used for computing ratio change, expressed as a percentage of R0 (the average R within the first 3 s of recording). Mechanical stimulation was carried out in Neuronal Buffer (145mM NaCl, 5mM KCl, 5mM $CaCl_2$, 5mM $MgCl_2$, 20mM glucose, 10mM HEPES, pH7.2). For comparison between mutant genotypes, calcium imaging was conducted blind to the genotype of the strains.

In vitro GPCR activation assays

Cell-based activation assays were performed as described (Beets et al., 2012). FRPR-3/C26F1.6 cDNA was cloned into the pcDNA3.1(+) TOPO expression vector (Thermo Fisher Scientific). Receptor activation was studied in Chinese hamster ovary cells (CHO) stably expressing apo-aequorin (mtAEQ) targeted to the mitochondria as well as the human $G\alpha_{16}$ subunit. The CHO-K1 cell line (PerkinElmer, ES-000-A2) was used for receptor activation assays. CHO/mtAEQ/$G\alpha_{16}$ cells were transiently transfected with the FRPR-3 cDNA construct or the empty pcDNA3.1(+) vector using the Lipofectamine transfection reagent (Thermo Fisher Scientific). Cells expressing the receptor were shifted to 28°C 1 day later, and collected 2 days post-transfection in BSA medium (DMEM/HAM's F12 with 15 mM HEPES, without phenol red, 0.1% BSA) loaded with 5 μM coelenterazine h (Thermo Fisher Scientific) for 4 h to reconstitute the holo-enzyme aequorin. Cells (25,000 cells/well) were exposed to synthetic peptides in BSA medium, and aequorin bioluminescence was recorded for 30 s on a MicroBeta LumiJet luminometer (PerkinElmer, Waltham Massachusetts) in quadruplicate. For dose-response evaluations, after 30 s of ligand-stimulated calcium measurements, Triton X-100 (0.1%) was added to the well to obtain a measure of the maximum cell Ca^{2+} response. BSA medium without peptides was used as a negative control and 1 μM ATP was used to check the functional response of the cells. Cells transfected with the empty vector were used as a negative control (not shown). EC_{50} values were calculated from dose-response curves, constructed using a nonlinear regression analysis, with sigmoidal dose-response equation (Prism 6.0).

Confocal microscopy

Images were acquired using a Zeiss LSM 710 or 780 and z stacks generated using Fiji (ImageJ).

QUANTIFICATION AND STATISTICAL ANALYSIS

The number of animals and replicates used per experiment is described in detail in the "Methods Details" subsection for each assay and in the relevant Figure legends. Specifically, for the main behavior tests: locomotor (tap) assays were conducted > 3 times with at least 3 plates of 50-100 animals each; cross-modal sensitization experiments were conducted with > 4 trials per condition of > 20 animals each.

Statistical analysis for all experiments was performed using GraphPad Prism 6.0. In general, where two groups were compared, an unpaired t test was used. Where multiple groups tested with a single condition were compared, a one-way ANOVA with Sidak's multiple comparisons post-test was used. Where multiple groups tested with multiple conditions were compared, a two-way ANOVA with Fisher's multiple comparisons post-test was used. Where appropriate, a D'Agostino & Pearson normality test was conducted to assess if the data fit a normal distribution.

DATA AND SOFTWARE AVAILABILITY

All data are available in electronic files.

Neuron, Volume *99*

Supplemental Information

An Afferent Neuropeptide System Transmits Mechanosensory Signals Triggering Sensitization and Arousal in *C. elegans*

Yee Lian Chew, Yoshinori Tanizawa, Yongmin Cho, Buyun Zhao, Alex J. Yu, Evan L. Ardiel, Ithai Rabinowitch, Jihong Bai, Catharine H. Rankin, Hang Lu, Isabel Beets, and William R. Schafer

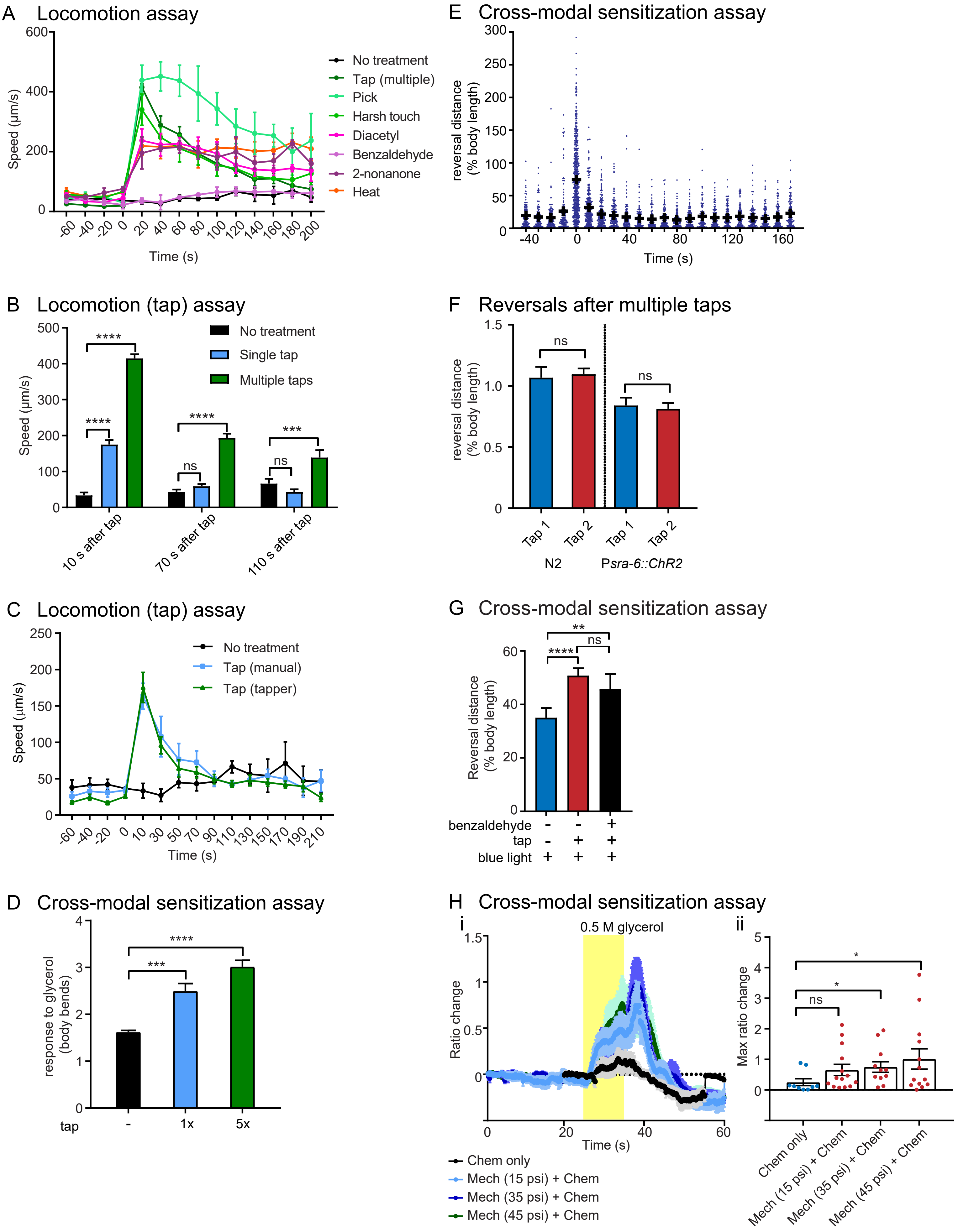
A Locomotion assay
Speed (µm/s)
Time (s)
No treatment
Tap (multiple)
Pick
Harsh touch
Diacetyl
Benzaldehyde
2-nonanone
Heat
B Locomotion (tap) assay
Speed (µm/s)
No treatment
Single tap
Multiple taps
****
****
****
ns
***
ns
10 s after tap
70 s after tap
110 s after tap
C Locomotion (tap) assay
Speed (µm/s)
No treatment
Tap (manual)
Tap (tapper)
Time (s)
D Cross-modal sensitization assay
response to glycerol (body bends)
****
***
tap
-
1x
5x
E Cross-modal sensitization assay
reversal distance (% body length)
Time (s)
F Reversals after multiple taps
reversal distance (% body length)
ns
ns
Tap 1
Tap 2
Tap 1
Tap 2
N2
Psra-6::ChR2
G Cross-modal sensitization assay
Reversal distance (% body length)
**
ns
****
benzaldehyde - - +
tap - + +
blue light + + +
H Cross-modal sensitization assay
i
0.5 M glycerol
Ratio change
Time (s)
Chem only
Mech (15 psi) + Chem
Mech (35 psi) + Chem
Mech (45 psi) + Chem
ii
Max ratio change
*
*
ns
Chem only
Mech (15 psi) + Chem
Mech (35 psi) + Chem
Mech (45 psi) + Chem

Figure S1, related to Figure 1: *C. elegans* displays locomotor and sensory arousal in response to aversive stimuli. A) Wild-type animals show an increased locomotion speed following the onset of various stimuli excluding benzaldehyde. Here, diacetyl is delivered at an aversive concentration (Yoshida et al., 2012). B) The locomotor response to tap stimulation in wild-type animals is dose dependent as animals exposed to multiple taps show a larger response that lasts longer than animals given a single tap. One-way ANOVA, Sidak's post-test: ns = not significant, ***<0.001, ****<0.0001. C) Speed trace for wild-type animals exposed to mechanical stimuli from manual tapping or automated tapper (see Experimental Procedures). For panels A-C, error bars indicate mean ± SEM, 5–10 animals were assayed in at least 5 trials for each condition. D-H: Cross-modal sensitization assay: D) Wild-type animals show increased reversal responses (expressed as number of body bends) to glycerol following a pre-arousing tap stimulus. One-way ANOVA, Sidak's post-test: ***<0.001, ****<0.0001. n=20 per condition, 4 trials. E) Controls exposed to tap (t=0) alone do not show increased reversal distance 20 s after tap. Animals received a 1x tap stimulus, following the protocol used for ASH sensitization experiments. "+" indicates the mean values for reversal distance. n>4 trials, each point represents an individual animal. F) Multiple taps do not appear to sensitize the reversal response in N2 and P*sra-6::ChR2* transgenic animals. n=5 trials. G) An attractive cue (benzaldehyde) does not affect sensitization in response to optogenetic activation of ASH after pre-exposure to a tap stimulus. Two-way ANOVA, Fisher's post-test: ns = not significant, **<0.01, ****<0.0001. n>15. For all panels, error bars indicate mean ± SEM. H) ASH neurons show a sensitized response to glycerol after exposure to a range of mechanosensory stimuli modulated by changing the pressure of the pneumatic valves (see Experimental procedures). Only the 15 psi condition did not show a statistically significant difference compared with controls exposed to glycerol alone. Note that previous work indicates that stimuli at pressures less than 40 psi are approximately equivalent to a tap stimulus, and pressures higher than 40 psi to harsh touch (Cho et al., 2018). Mean traces are shown in (i) and quantification of maximal responses is shown in (ii). For ease of comparison, the average trace during glycerol perfusion for non-pre-aroused controls is temporally aligned with that of other groups. For F-H, error bars indicate mean ± SEM.

A Nonanone sensitization assay

i. Glycerol stimulus only

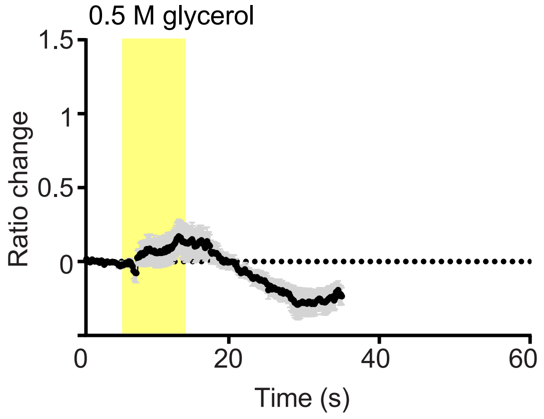

ii. Nonanone then glycerol stimulus

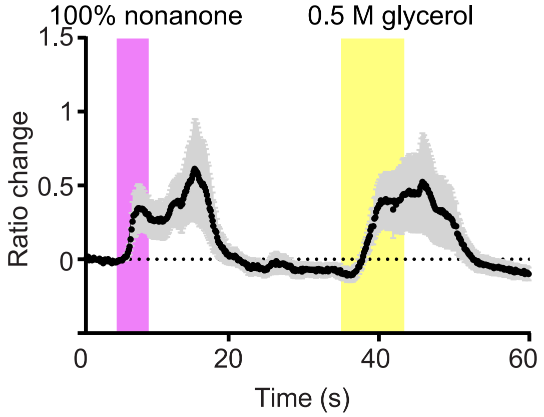

B Nonanone sensitization assay

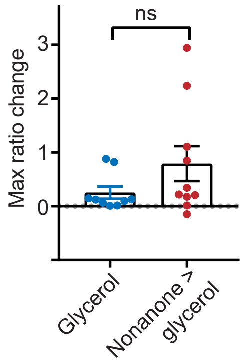

C Nonanone sensitization assay

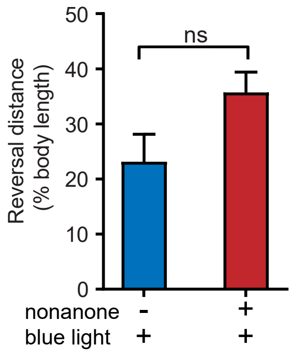

D Gentle touch assay

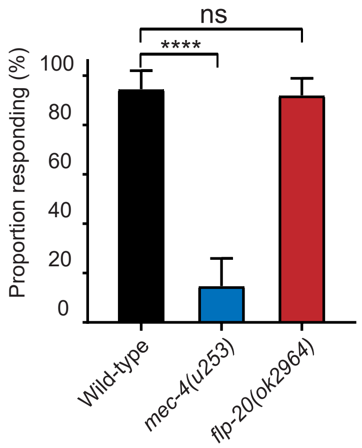

Figure S2, related to Figure 1 and 2: Aversive odorants do not sensitize ASH responses and FLP-20 is dispensable for response to gentle touch. A-C) Nonanone sensitization assay: Panel A shows the average traces of ASH calcium activity after exposure to glycerol either (i) alone or (ii) following a 5 s nonanone pulse ($n = 9$-10), panel B shows the quantification of maximal responses (p-value = 0.1465), and panel C shows the reversal distance for *ASH::ChR2* animals in response to optogenetic activation of ASH with or without pre-exposure to nonanone. Unpaired t-test: ns = not significant. $n > 7$. D) Touch assay for *flp-20(ok2964)* mutants compared with wild-type and *mec-4(u253)* controls showing the proportion of animals responding to alternating anterior and posterior body touch with an eyelash hair. One-way ANOVA, Sidak's post-test: ns = not significant, ****<0.0001. n=20. For all panels, error bars indicate mean ± SEM

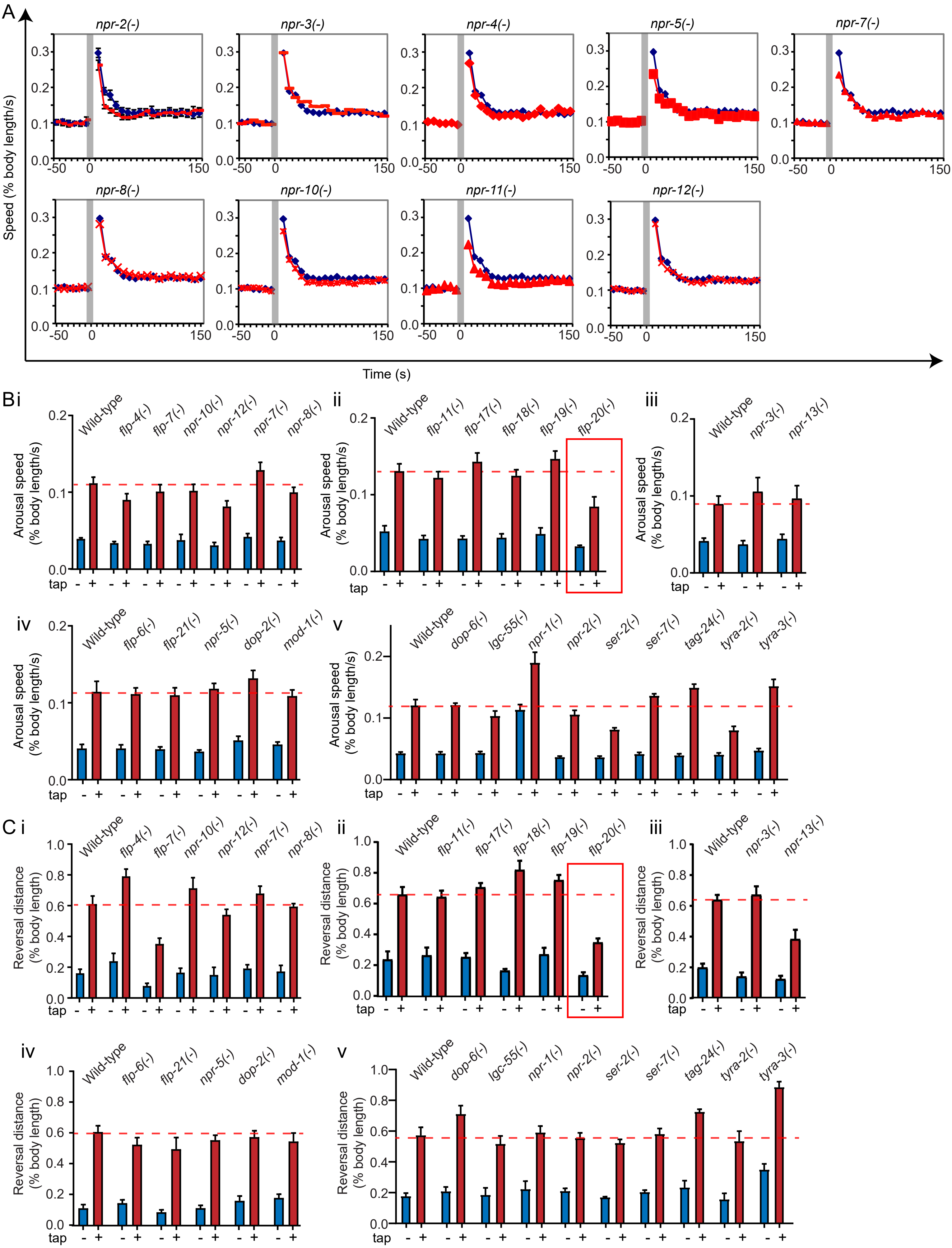
A
npr-2(-)
npr-3(-)
npr-4(-)
npr-5(-)
npr-7(-)
npr-8(-)
npr-10(-)
npr-11(-)
npr-12(-)
Speed (% body length/s)
Time (s)
B i
ii
iii
iv
v
Arousal speed (% body length/s)
Wild-type
flp-4(-)
flp-7(-)
npr-10(-)
npr-12(-)
npr-7(-)
npr-8(-)
flp-11(-)
flp-17(-)
flp-18(-)
flp-19(-)
flp-20(-)
npr-3(-)
npr-13(-)
flp-6(-)
flp-21(-)
npr-5(-)
dop-2(-)
mod-1(-)
dop-6(-)
lgc-55(-)
npr-1(-)
npr-2(-)
ser-2(-)
ser-7(-)
tag-24(-)
tyra-2(-)
tyra-3(-)
tap
C i
Reversal distance (% body length)

Figure S3, related to Figure 2: Candidate screen for mutant strains that show defects in arousal. A) Speed traces from a subset of the candidate mutant strains (red line) tested for locomotion speed in response to tap (onset of tap is shown by the vertical grey bar) compared to controls (blue line). Due to high variation in the baseline locomotion speed of these mutants, speed is normalized to the baseline speed before tap in all traces shown. n> 4 trials. B) Locomotion (tap) assay: quantified speed (%body length/s) before and after mechanical (tap) stimulation for the candidate mutant strains tested. C) Cross-modal sensitization assay: reversal distance (%body length) in response to optogenetic stimulation of ASH with blue light with or without a pre-arousing tap stimulus. All mutant strains were crossed into transgenic lines containing the *sra-6::ChR2* or *ASH::ChR2* transgene (see **Table S1** strain list). Experiments performed with different controls are shown in separate graphs. Dashed line indicates the values for controls in each experiment. *flp-20* mutants (red boxes) showed a reliable and clear difference in both locomotor and sensory sensitization compared with controls. n> 5 trials.

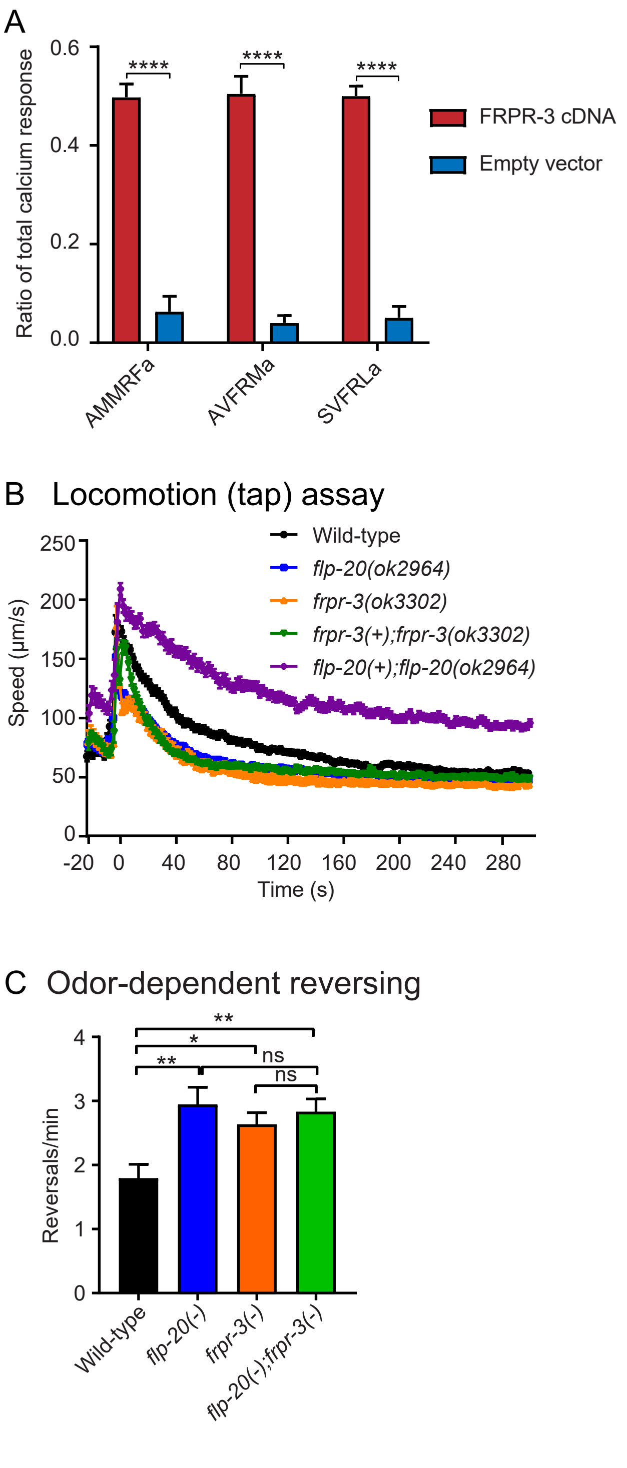
A
Ratio of total calcium response
0.6
0.4
0.2
0.0
****
****
****
FRPR-3 cDNA
Empty vector
AMMRFa
AVFRMa
SVFRLa
B Locomotion (tap) assay
Speed (µm/s)
250
200
150
100
50
0
-20 0 40 80 120 160 200 240 280
Time (s)
Wild-type
flp-20(ok2964)
frpr-3(ok3302)
frpr-3(+);frpr-3(ok3302)
flp-20(+);flp-20(ok2964)
C Odor-dependent reversing
Reversals/min
4
3
2
1
0
**
*
**
ns
ns
Wild-type
flp-20(-)
frpr-3(-)
flp-20(-);frpr-3(-)

Figure S4, related to Figure 4 and 5: FRPR-3 is required for locomotor arousal and for FLP-20-dependent responses to attractive chemosensory cues. A) Activation of FRPR-3 by FLP-20 peptides does not require $G\alpha_{16}$. Expression of *frpr-3* cDNA in cells expressing aequorin but lacking $G\alpha_{16}$ showed robust calcium transients in response to FLP-20 peptides compared to controls (empty pcDNA3.1(+) vector). Calcium responses are displayed as a ratio of total responses. n = 6. Unpaired t-test: ****<0.0001. B) Speed traces for wild-type, *flp-20* and *frpr-3* mutant animals together with transgenic animals re-expressing *flp-20* or *frpr-3* using the endogenous promoter for these genes. The tap stimulus is applied at time = 0. n>200 for at least three trials. C) Off-food reversals for *flp-20* and *frpr-3* single mutants and *flp-20;frpr-3* double mutant animals. One-way ANOVA, Sidak's post-test: ns = not significant, *<0.05, **<0.01. For all panels, error bars indicate mean ± SEM

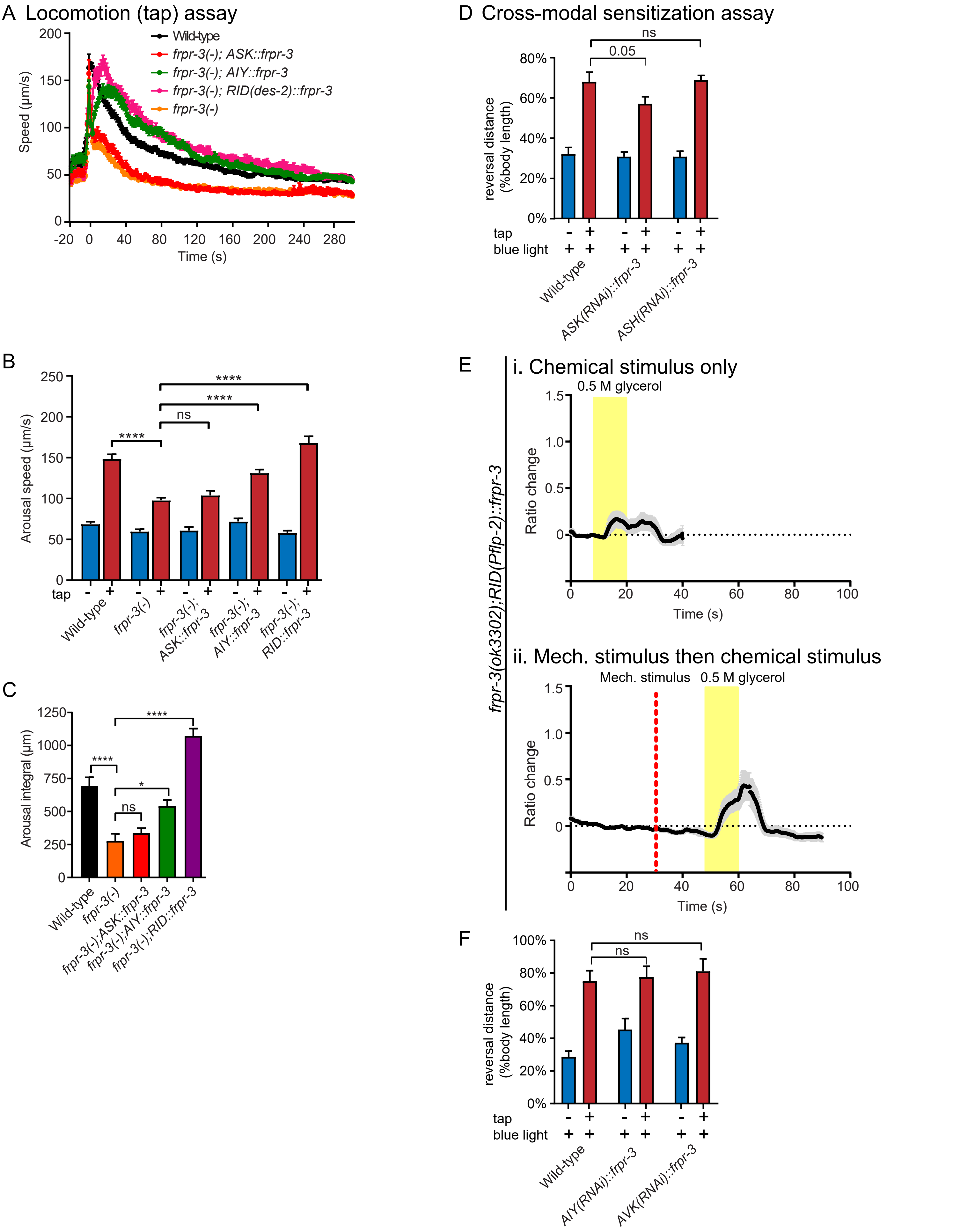
A Locomotion (tap) assay
Wild-type
frpr-3(-); ASK::frpr-3
frpr-3(-); AIY::frpr-3
frpr-3(-); RID(des-2)::frpr-3
frpr-3(-)
Speed (µm/s)
Time (s)
D Cross-modal sensitization assay
ns
0.05
reversal distance (%body length)
tap
blue light
Wild-type
ASK(RNAi)::frpr-3
ASH(RNAi)::frpr-3
B
Arousal speed (µm/s)
****
ns
tap
Wild-type
frpr-3(-)
frpr-3(-); ASK::frpr-3
frpr-3(-); AIY::frpr-3
frpr-3(-); RID::frpr-3
C
Arousal integral (µm)
****
ns
*
Wild-type
frpr-3(-)
frpr-3(-);ASK::frpr-3
frpr-3(-);AIY::frpr-3
frpr-3(-);RID::frpr-3
E
frpr-3(ok3302);RID(Pflp-2)::frpr-3
i. Chemical stimulus only
0.5 M glycerol
Ratio change
Time (s)
ii. Mech. stimulus then chemical stimulus
Mech. stimulus
0.5 M glycerol
Ratio change
Time (s)
F
ns
ns
reversal distance (%body length)
tap
blue light
Wild-type
AIY(RNAi)::frpr-3
AVK(RNAi)::frpr-3

Figure S5, related to Figure 6: FRPR-3 is required in RID and AIY for locomotor arousal and in RID for sensory sensitization. A-C) Locomotion arousal assays for wild-type and *frpr-3* mutant animals together with transgenic animals re-expressing *frpr-3* using the promoters driving expression in AIY (P*ttx-3*), ASK (P*sra-9*) or RID (P*des-2*). Panel A shows the speed traces for genotypes tested, with the tap stimulus applied at time = 0. Quantification of B) arousal speed and C) arousal integral after tap. For panels A-C, n> 200 for at least 2 trials. Error bars indicate mean ± SEM. One-way ANOVA, Sidak's post-test: ns = not significant, *<0.05, ****<0.0001. D-F) Cross-modal sensitization assays: D) Reversal responses for wild-type, *ASK(RNAi)::frpr-3* and *ASH(RNAi)::frpr-3* animals following blue light stimulation, with or without a pre-arousing tap. E) Mean traces of ASH calcium activity in *frpr-3(ok3302);RID(Pflp-2)::frpr-3* transgenic animals measured with GCaMP3 after exposure to glycerol either (i) alone or (ii) following a mechanical stimulus applied to the body of the animal. n=14-19. F) Reversal responses for wild-type, *AIY(RNAi)::frpr-3* and *AVK(RNAi)::frpr-3* animals following blue light stimulation, with or without a pre-arousing tap. For panels D,F: Two-way ANOVA, Fisher's post-test: ns = not significant. n>6 trials. Error bars indicate mean ± SEM.

A Micrographs for intersectional promoter strategy

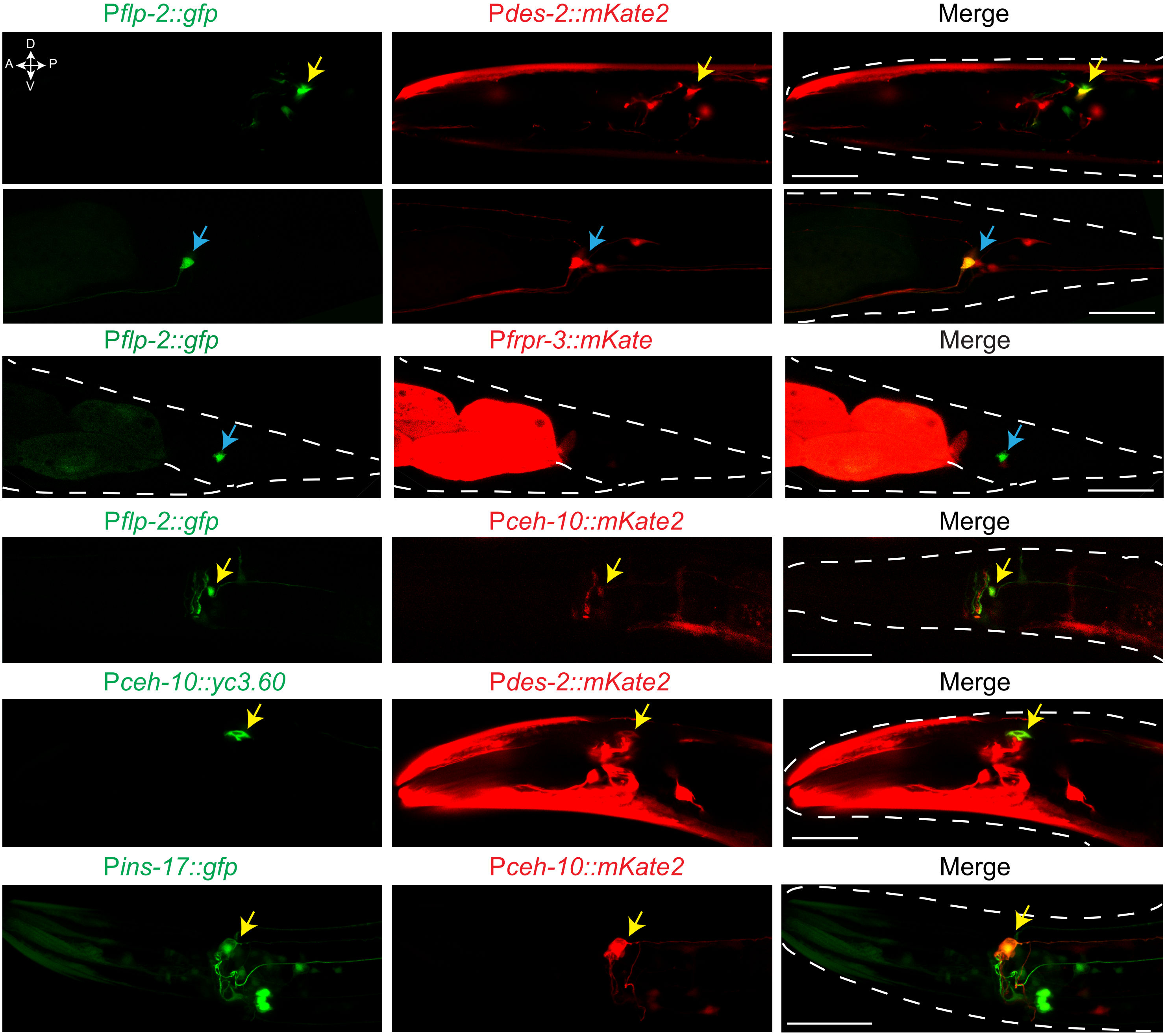

B Cross-modal sensitization assay

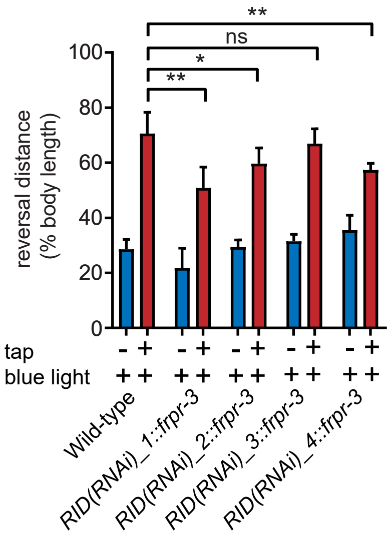

Figure S6, related to Figure 6: FRPR-3 is required in RID for ASH sensory sensitization. A) Micrographs showing the overlap between promoters used for the intersectional strategy for expression in RID. RID is indicated by the yellow arrow. P*flp-2::gfp*/P*des-2::mKate2* also overlap in an unidentified tail neuron (blue arrow) that does not colocalise with P*frpr-3::mKate2*. Scale bar = 10 µm. B) Cross-modal sensitization assay: Reversal responses following blue light stimulation for transgenic lines expressing antisense/sense RNAi constructs to knockdown *frpr-3* using four intersectional promoter combinations overlapping in RID, with or without a pre-arousing tap. The promoter combinations are: #1: P*flp-2/*P*des-2*; #2: P*flp-2/*P*ceh-10*; #3: P*des-2*/P*ceh-10*; #4: P*ins-17/*P*ceh-10*. See strain list **Table S1** for details. Error bars indicate mean ± SEM. Two-way ANOVA, Fisher's post-test: ns = not significant, *<0.05, **<0.01. n>4 trials.

A

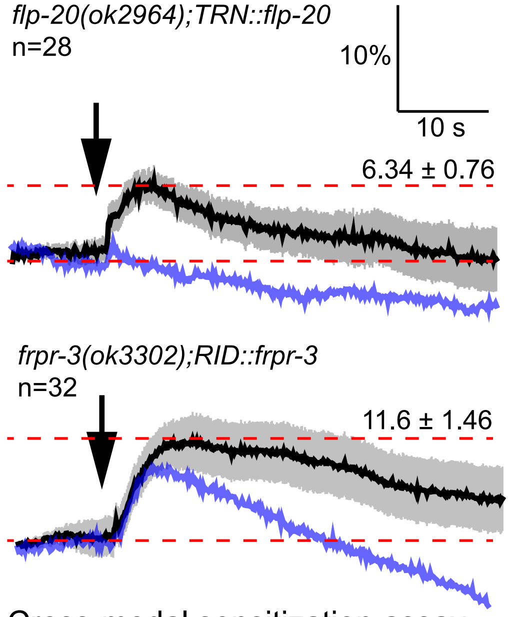

B Cross-modal sensitization assay

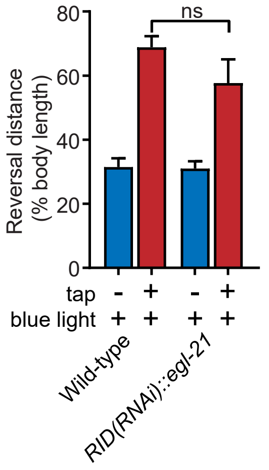

C Locomotor arousal (tap) assay

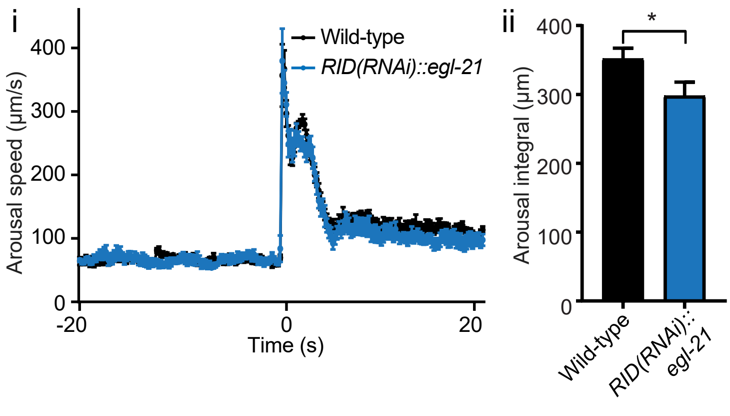

Figure S7, related to Figure 6: RID modulates arousal in response to mechanosensory stimulation via FLP-20/FRPR-3 and by signaling to downstream sensory targets partially via neuropeptides. A) Mean traces for transgenic lines re-expressing *flp-20* in the TRNs (using P*mec-4*) (*flp-20(ok2964);TRN::flp-20*) and *frpr-3* in RID (using P*ceh-10*) (*frpr-3(ok3302);RID::frpr-3*). For comparison, the mean traces (in blue) for the respective mutant strains are overlaid. The maximum ratio change for YFP/CFP comparing post-stimulus intensities with baseline intensities is shown as mean ± SEM for each trace. The number of worms tested is shown, with each worm stimulated 1-3 times. Scale shows % ratio change on the y-axis and time (s) on the x-axis. B) Cross-modal sensitization assay: Reversal responses for wild-type and *RID(RNAi)::egl-21* transgenic animals following blue light stimulation, with or without a pre-arousing tap. Two-way ANOVA, Fisher's post-test: ns = not significant. n = 6. C) i) Speed traces and ii) arousal integral for *RID(RNAi)::egl-21* and control animals after a 1x tap stimulus at t=0. Unpaired t-test: *<0.05. n = 6.

Table S1: Strain list, related to STAR Methods. For transgenic lines, the number following the transgene refers to the concentration injected in ng/μL.

Strain number	Genotype information	Notes	Experiment
AQ4045	ljEx1107[Psra-6::GCaMP3::SL2-tagRFP(50);unc-122::RFP(50)]		ASH calcium imaging (microfluidics)
AQ4076	flp-20(ok2964);ljEx1107		ASH calcium imaging (microfluidics)
AQ4077	frpr-3(ok3302);ljEx1107		ASH calcium imaging (microfluidics)
AQ4168	frpr-3(ok3302);ljEx1149[Pdes-2::frpr-3cDNA::gpd-2 3' UTR(pYLC219)(25);unc-122::gfp(50)];ljEx1107	RID(+)::frpr-3	ASH calcium imaging (microfluidics)
AQ4169	frpr-3(ok3302);ljEx1150[Pflp-2::frpr-3cDNA::gpd-2 3' UTR(pYLC220)(25);unc-122::gfp(50)];ljEx1107	RID(+)::frpr-3	ASH calcium imaging (microfluidics)
AQ4173	flp-20(ok2964);ljEx1094[Pmec-4::flp-20 gDNA + 3' UTR::SL2-mKate2 (50);unc-122::gfp(50)];ljEx1107	TRN::flp-20	ASH calcium imaging (microfluidics)
AQ2052	lite-1(ce314);ljIs105[sra-6::ChR2::yfp, unc-122::gfp]		Cross-modal sensitization assay
AQ2235	lite-1(ce314); ljIs114[Pgpa-13::FLPase, Psra-6::FTF::ChR2::YFP] X		Cross-modal sensitization assay
AQ2755	lite-1(ce314); ljIs124[Pgpa-13::FLPase, Psra-6::FTF::ChR2::YFP]not X		Cross-modal sensitization assay
VG266	frpr-3(gk240031) (backcrossed 3x); lite-1(ce314); ljIs114		Cross-modal sensitization assay
AQ2786	flp-20 (ok2964) lite-1(ce314) X; ljIs124		Cross-modal sensitization assay
AQ3941	lite-1(ce314) flp-20(ok2964)X; ljIs124; Ex[Pmec-4::flp-20 cDNA)(20);unc-122::gfp(20)] line-1	TRN::flp-20	Cross-modal sensitization assay
AQ3940	lite-1(ce314) flp-20(ok2964)X; ljIs124; Ex[Pflp-20::flp-20 cDNA (20), ccGFP (20)] line-1	flp-20::flp-20	Cross-modal sensitization assay
AQ4246	lite-1(ce314) flp-20(ok2964)X; ljIs124; Ex[Pflp-20::flp-20 cDNA (20), ccGFP (20)] line-2	flp-20::flp-20	Cross-modal sensitization assay
AQ4247	lite-1(ce314) flp-20(ok2964)X; ljIs124; Ex[Pflp-20::flp-20 cDNA (20), ccGFP (20)] line-3	flp-20::flp-20	Cross-modal sensitization assay
AQ4248	lite-1(ce314) flp-20(ok2964)X; ljIs124; Ex[Pmec-4::flp-20 cDNA)(20);unc-122::gfp(20)] line-2	TRN::flp-20	Cross-modal sensitization assay
AQ4249	lite-1(ce314) flp-20(ok2964)X; ljIs124; Ex[Pmec-4::flp-20 cDNA)(20);unc-122::gfp(20)] line-3	TRN::flp-20	Cross-modal sensitization assay
AQ4250	lite-1(ce314) flp-20(ok2964)X; ljIs124; Ex[Pgcy-5::flp-20(10); Pgcy-7::flp-20(10);unc-122::gfp(20)] line-1	ASE::flp-20	Cross-modal sensitization assay
AQ4251	lite-1(ce314) flp-20(ok2964)X; ljIs124; Ex[Pgcy-5::flp-20(10); Pgcy-7::flp-20(10);unc-122::gfp(20)] line-2	ASE::flp-20	Cross-modal sensitization assay
AQ4252	lite-1(ce314) flp-20(ok2964)X; ljIs124; Ex[Pgcy-5::flp-20(10); Pgcy-7::flp-20(10);unc-122::gfp(20)] line-3	ASE::flp-20	Cross-modal sensitization assay
AQ4260	lite-1(ce314);ljIs124;ljEx1187[Psra-6::frpr-3 antisense RNAi(50);Psra-6::frpr-3 sense RNAi(50);ccGFP(30)]	ASH(RNAi)::frpr-3	Cross-modal sensitization assay
AQ4262	lite-1(ce314);ljIs124;ljEx1189[Psra-9::frpr-3 antisense RNAi(55);Psra-9::frpr-3 sense RNAi(55);ccGFP(30)]	ASK(RNAi)::frpr-3	Cross-modal sensitization assay
AQ4264	lite-1(ce314);ljIs124;ljEx1191[Pflp-2::frpr-3 antisense RNAi(50);Pdes-2::frpr-3 sense RNAi(50);ccGFP(30)]	RID(RNAi)_1::frpr-3	Cross-modal sensitization assay
AQ4320	lite-1(ce314);ljIs124;ljEx1214[Pdes-2::egl-21 antisense RNAi(50);Pflp-2::egl-21 sense RNAi(50);ccGFP(40)]	RID(RNAi)::egl-21	Cross-modal sensitization assay
AQ4346	lite-1(ce314);ljIs124;ljEx1228[Pflp-2::frpr-3 antisense RNAi(50);Pceh-10(3.6)::frpr-3 sense RNAi(50);ccGFP(50)]	RID(RNAi)_2::frpr-3	Cross-modal sensitization assay
AQ4355	lite-1(ce314);ljIs124;ljEx1229[Pdes-2::frpr-3 antisense RNAi(50);Pceh-10(3.6)::frpr-3 sense RNAi(50);ccGFP(50)]	RID(RNAi)_3::frpr-3	Cross-modal sensitization assay
AQ4356	lite-1(ce314);ljIs124;ljEx1230[Pins-17(2kb)::frpr-3 antisense RNAi(50);Pceh-10(3.6)::frpr-3 sense RNAi(50);ccGFP(50)]	RID(RNAi)_4::frpr-3	Cross-modal sensitization assay
AQ4360	lite-1(ce314);ljIs124;ljEx1234[Pflp-2::unc-31 antisense RNAi(50);Pdes-2::unc-31 sense RNAi(50);ccGFP(50))]	RID(RNAi)::unc-31	Cross-modal sensitization assay
AQ4364	lite-1(ce314);ljIs124;ljEx1238[Pttx-3::frpr-3 antisense RNAi(50);Pttx-3::frpr-3 sense RNAi(50);unc-122::gfp(50)]	AIY(RNAi)::frpr-3	Cross-modal sensitization assay
AQ4365	lite-1(ce314);ljIs124;ljEx1239[Pflp-1::frpr-3 antisense RNAi(50);Pflp-1::frpr-3 sense RNAi(50);unc-122::gfp(50)]	AVK(RNAi)::frpr-3	Cross-modal sensitization assay
AQ4390	lite-1(ce314);ljIs124;hpIs626(Pceh10::Chrimson::GFP::ZF; Pttx-3::ZIF-1::SL2::RFP; Pgpa-14::ZIF-1::SL2::RFP);hpEx3808(Parr-1-ZIF-1::SL2::RFP;Pmyo-3::rfp)	AQ2755 x ZM9315	Cross-modal sensitization assay
AQ4023	ljEx1093[Pflp-20::flp-20 gDNA + 3' UTR::SL2-mKate2(50);unc-122::gfp(50)]		Expression pattern for flp-20
AQ4006	ljEx1090[Pfrpr-3::frpr-3 gDNA::SL2-mKate(25); unc-122::gfp(50)]		Expression pattern for frpr-3
TU253	mec-4(u253) X		Gentle touch
AQ4019	frpr-3(ok3302);ljEx1090	frpr-3::frpr-3	Locomotion (tap) arousal assay
AQ4035	flp-20(ok2964);ljEx1094	TRN::flp-20	Locomotion (tap) arousal assay
AQ4037	flp-20(ok2964);ljEx1093	flp-20::flp-20	Locomotion (tap) arousal assay
AQ4054	frpr-3(ok3302);flp-20(ok2964)		Locomotion (tap) arousal assay
AQ4072	flp-20(ok2964);frpr-3(ok3302);ljEx1093		Locomotion (tap) arousal assay
AQ4078	frpr-3(ok3302);ljEx1108[Pflp-1::frpr-3 gDNA + UTR(10);unc-122::GFP(50)]	AVK::frpr-3	Locomotion (tap) arousal assay
AQ4104	frpr-3(ok3302);ljEx1135[Pflp-2::frpr-3::SL2-mKate2 (50);unc-122::gfp(50)]	RID(+)::frpr-3	Locomotion (tap) arousal assay
AQ4105	frpr-3(ok3302);ljEx1136[Pdes-2::frpr-3::SL2-mKate2 (50);unc-122::gfp(50)]	RID(+)::frpr-3	Locomotion (tap) arousal assay
AQ4179	frpr-3(ok3302);ljEx1167[Pceh-10(3.6)::frpr-3::SL2-mKate2(pYLC232)(50);unc-122::gfp(50)]	RID(+)::frpr-3	Locomotion (tap) arousal assay
AQ4087	frpr-3(ok3302);ljEx1123[Psra-9::frpr-3 gDNA + 3' UTR::SL2-mKate2(50);ccGFP(50)]	ASK::frpr-3	Locomotion (tap) arousal assay
AQ4103	frpr-3(ok3302);ljEx1134[Pttx-3::frpr-3::SL2-mKate2 (50)(pYLC190);ccGFP(50)]	AIY::frpr-3	Locomotion (tap) arousal assay
AQ3832	frpr-3(ok3302) V backcrossed 6x		Locomotion (tap)
AQ4000	flp-20(ok2964) X backcrossed 6x		Locomotion (tap), gentle touch
AQ4396	ljEx1246[Pdes-2::mKate2::gpd-2 3' UTR(25);ccGFP(50)];ljEx1247[Pflp-2:gfp::gpd-2 3' UTR(25);ccRFP(50)]		Micrographs for RID intersectional promoter
AQ4397	ljEx1246[Pdes-2::mKate2::gpd-2 3' UTR(25);ccGFP(50)];ljEx1165[Pceh-10(3.6)::YC3.60::gpd-2 3' UTR(25);ccRFP(50)]		Micrographs for RID intersectional promoter
AQ4398	unc-119(ed3) III; wwEx73(ins-17p::gfp + unc-119(+));ljEx1167[Pceh-10(3.6)::frpr-3::SL2-mKate2(50);ccGFP(50)]	ins-17::gfp from HT1734	Micrographs for RID intersectional promoter
AQ4399	ljEx1167[Pceh-10(3.6)::frpr-3::SL2-mKate2(50);ccGFP(50)];ljEx1247[Pflp-2:gfp::gpd-2 3' UTR(50);ccRFP(50)]		Micrographs for RID intersectional promoter
PT505	flp-20(pk1596) X		Off food reversal assay
VC2565	frpr-3(ok3302) V		Off food reversal assay
BJH387	flp-20(pk1596) X; frpr-3(ok3302) V		Off food reversal assay
AQ4144	ljEx1165[Pceh-10(3.6)::YC3.60::gpd-2 3' UTR(50);unc-122::rfp(50)]		RID calcium imaging (glued protocol)
AQ4187	frpr-3(ok3302);ljEx1165		RID calcium imaging (glued protocol)
**Strain number**	**Genotype information**	**Notes**	**Experiment**

AQ4188	*flp-20(ok2964);ljEx1165*		RID calcium imaging (glued protocol)
AQ4210	*flp-20(ok2964);ljEx1094;ljEx1165*	*TRN::flp-20*	RID calcium imaging (glued protocol)
AQ4211	*frpr-3(ok3302);ljEx1167;ljEx1165*	*RID(+)::frpr-3*	RID calcium imaging (glued protocol)
AQ2766	*lite-1(ce314);ljIs114 X (ASH::ChR2);dop-2 (vs105)V*		Candidate screen
AQ2767	*lite-1(ce314);ljIs114 X (ASH::ChR2);npr-3(tm1583)*		Candidate screen
AQ2768	*lite-1(ce314);ljIs114 X (ASH::ChR2);npr-5 (ok1583)V*		Candidate screen
AQ2769	*lite-1(ce314);ljIs114 X (ASH::ChR2);npr-13 (tm1504)V*		Candidate screen
AQ2770	*lite-1(ce314);ljIs114 X (ASH::ChR2);flp-6 (ok3056) V*		Candidate screen
AQ2772	*lite-1(ce314);ljIs114 X (ASH::ChR2);flp-21 (ok889)V*		Candidate screen
AQ2773	*lite-1(ce314);ljIs114 X (ASH::ChR2);mod-1 (ok103)V*		Candidate screen
AQ2779	*lite-1(ce314);ljIs114 X (ASH::ChR2);flp-4 II*		Candidate screen
AQ2781	*lite-1(ce314);ljIs124 non-X (ASH::ChR2); tyra-3 (ok325)X*		Candidate screen
AQ2782	*lite-1(ce314);ljIs124 non-X (ASH::ChR2); npr-1 (ad609)X*		Candidate screen
AQ2783	*lite-1(ce314);ljIs124 non-X (ASH::ChR2); flp-7 (ok2625)X*		Candidate screen
AQ2785	*lite-1(ce314);ljIs124 non-X (ASH::ChR2); flp-18 (dp99)X*		Candidate screen
AQ2786	*lite-1(ce314);ljIs124 non-X (ASH::ChR2); flp-20 (ok2964)X*		Candidate screen
AQ4405	*ljIs105(Psra-6::ChR2); ser-7 (tm1325)X*		Candidate screen
AQ4406	*ljIs105(Psra-6::ChR2);tag-24 (ok371)X*		Candidate screen
AQ4407	*ljIs105(Psra-6::ChR2);ser-2 (pk1357)X*		Candidate screen
AQ4408	*ljIs105(Psra-6::ChR2);tyra-2 (tm1846)X*		Candidate screen
AQ4409	*ljIs105(Psra-6::ChR2);tyra-3 (ok325)X*		Candidate screen
AQ4410	*ljIs105(Psra-6::ChR2);lgc-55 (tm2913)V*		Candidate screen
AQ4411	*ljIs105(Psra-6::ChR2);dop-6/C24A8.1 (ok2090)X*		Candidate screen
AQ4412	*ljIs105(Psra-6::ChR2);npr-1 (ad609)X*		Candidate screen
AQ4413	*ljIs105(Psra-6::ChR2);npr-2 (ok419)IV*		Candidate screen
AQ4414	*ljIs105(Psra-6::ChR2);npr-7 (ok527)X*		Candidate screen
AQ4415	*ljIs105(Psra-6::ChR2);npr-8 (tm1553)X*		Candidate screen
AQ4416	*ljIs105(Psra-6::ChR2);npr-10 (tm1568)X*		Candidate screen
AQ4417	*ljIs105(Psra-6::ChR2);npr-11 (ok594)X*		Candidate screen
AQ4418	*ljIs105(Psra-6::ChR2);npr-12 (tm1498)IV*		Candidate screen
AQ4419	*ljIs105(Psra-6::ChR2);flp-11 (tm2706)X*		Candidate screen
AQ4420	*ljIs105(Psra-6::ChR2);flp-17 (ok3587)IV*		Candidate screen
AQ4421	*ljIs105(Psra-6::ChR2);flp-18 (dp99)X*		Candidate screen
AQ4422	*ljIs105(Psra-6::ChR2);flp-19 (ok2460)X*		Candidate screen

Table S2: Reporter lines used for identification of *flp-20* and *frpr-3*- expressing cells, related to STAR Methods.

Gene	Cell	Promoter	Reporter line
flp-20	TRNs	*mec-4*	AQ906 *bzIs17[Pmec-4, yc2.12+lin-15]*
	PVC	*nmr-1*	AQ2307 *ljEx226[Pnmr-1::YC3.60]*
	LUA	*inx-11*	ZW291 *zwEx111[Pinx-11:GFP + lin-15(+)]*
	ASE	*gcy-7*	AQ4095 *ljEx1129[Pgcy-7::gfp]*
frpr-3	AVK	*flp-1*	NY2097 *ynIs97 [Pflp-1::GFP]*
	AIY	*ttx-3*	AQ3040 *ljEx521[Pttx-3::YC3.60]*
	RID	*ceh-10*	LE332 *lqIs10[Pceh-10::GFP + lin-15(+)]*
	ASK	*sra-9*	AQ3093 *ljEx543[Psra-9::YC3.60]*
